# Structure and thermal stability of phosphorus-iodonium ylids

**DOI:** 10.3762/bjoc.20.245

**Published:** 2024-11-14

**Authors:** Andrew Greener, Stephen P Argent, Coby J Clarke, Miriam L O’Duill

**Affiliations:** 1 School of Chemistry, University of Nottingham, University Park, Nottingham NG7 2RD, UK

**Keywords:** hypervalent iodine, reagent development, structural analysis, thermal stability, thermogravimetric analysis

## Abstract

Hypervalent iodine(III) reagents have become indispensable tools in organic synthesis, but gaps remain in the functionalities they can transfer. In this study, a fundamental understanding of the thermal stability of phosphorus-iodonium ylids is obtained through X-ray diffraction, differential scanning calorimetry (DSC) and thermogravimetric analysis (TGA). Insights into the structural factors affecting thermal stability and potential decomposition pathways will enable the future design and development of new reagents.

## Introduction

Hypervalent iodine(III) reagents have experienced a renaissance in synthetic organic chemistry, becoming indispensable tools in total synthesis, late-stage functionalisation and radiolabelling [1–9]. Due to their great mechanistic flexibility, including reactivity as oxidants, electrophiles, radical precursors and transmetalating agents, they often enable access to chemical motifs that are difficult to synthesise using traditional approaches. However, gaps remain in the functionality they can transfer. Specifically, unstabilised alkyl groups are still underrepresented. For the development of new hypervalent iodine reagents to bridge this gap, it is vital to gain a fundamental understanding of the structural factors affecting their stability and reactivity.

**Figure 1:**
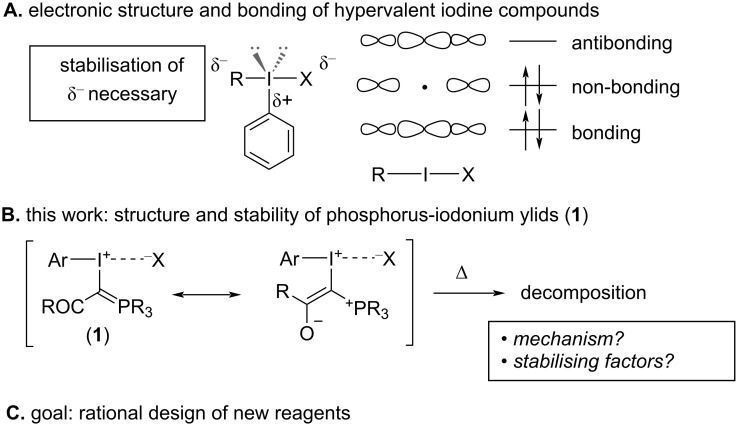
Structure and stability of hypervalent iodine compounds.

Previous reports have suggested a link between structural factors and thermal stability of hypervalent iodine compounds [10–16]. Iodine(III) compounds are generally trigonal bipyramidal (T-shaped) with the least electronegative group and the two nonbonding electron pairs occupying the equatorial positions, and the most electronegative substituents forming a hypervalent 3-centre-4-electron (3c-4e) bond in the axial position (Figure 1A) [17–18]. The LUMO of this bond is concentrated on iodine [19], making it highly electrophilic, while a nonbonding pair of electrons is mainly centred on the axial substituents, causing a build-up of electron density on these positions (Figure 1A) [1,20]. Stabilisation of this charge on the axial substituents by strong electron-withdrawing groups or delocalisation into a π-system results in crystalline, bench-stable reagents. In the absence of stabilising factors, rapid decomposition occurs [21–23].

In this study, we aim to gain a fundamental understanding of the factors that stabilise phosphorus-iodonium ylids **1** (Figure 1B) [24–27] and the mechanisms by which they decompose when heated through a systematic investigation of structural data from X-ray diffraction (XRD) and thermal stability data from differential scanning calorimetry (DSC) and thermogravimetric analysis (TGA). The insights from this study will galvanise the rational design and synthesis of novel, unstabilised hypervalent iodine(III) compounds and expand the application of these powerful reagents in organic synthesis.

## Results and Discussion

### Structural data

**Figure 2:**
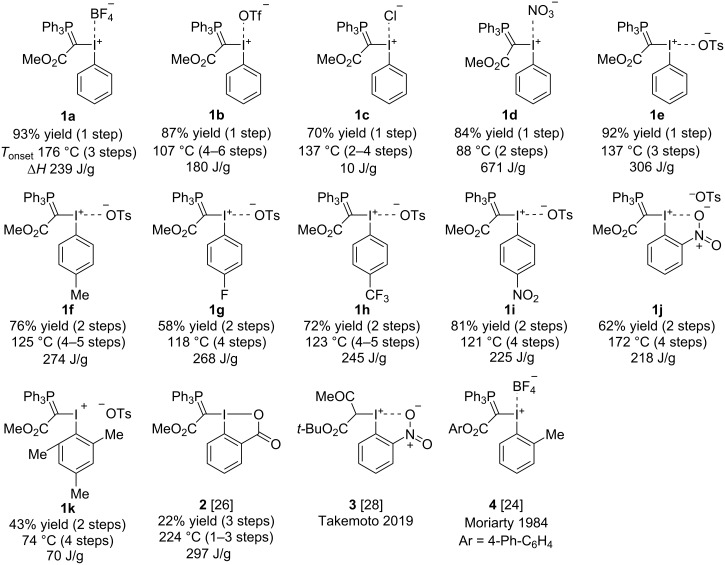
Phosphorus-iodonium ylids investigated in this study: Yields (for synthetic procedures, see Supporting Information File 2), structure and thermal data. The decomposition onset temperature, *T*_onset_, and the number of decomposition steps (in brackets) were determined by TGA measurements, Δ*H* was obtained by DSC.

Twelve phosphorus-iodonium ylids were synthesised (Figure 2). X-ray diffraction data (XRD) of compounds **1a–f** and **1i** were measured (see Supporting Information File 1), and data for compounds **2**–**4** were sourced from the literature [24,26,28]. A representative set of structural parameters obtained from XRD is presented in Table 1.

**Table 1:** XRD data.

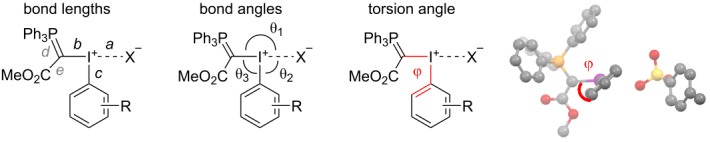									

	Bond lengths [Å]^a^					Bond angles [°]			Torsion angle [°]
	a	b	c	d	e	θ_1_	θ_2_	θ_3_	φ

**1a**	4.165(2)	2.026(7)	2.108(7)	1.742(7)	1.421(9)	98.2(4)	158.85(5)	99.2(3)	29.5(7)
**1b**	3.069(3)	2.026(4)	2.129(4)	1.740(5)	1.454(7)	90.6(1)	160.6(2)	100.0(2)	38.3(5)
**1c**	3.1411(5)	2.051(2)	2.134(2)	1.736(2)	1.422(3)	89.40(6)	174.24(5)	95.92(8)	40.8(2)
**1d** ^b^	3.16(1)^b^	2.07(2)	2.12(1)	1.72(1)	1.39(2)	101.6(5)^b^	157.7(5)^b^	97.0(6)	50.0(1)
**1e**	2.910(4)	2.048(5)	2.123(6)	1.736(6)	1.430(7)	169.1(2)	77.5(2)	97.2(2)	41.5(5)
**1f-h1** ^c^	2.839(6)	2.050(7)	2.133(8)	1.736(7)	1.417(13)	170.0 (3)	84.25(3)	96.6(3)	46.2(7)
**1f-h2** ^c^	2.832(7)	2.042(7)	2.107(7)	1.726(7)	1.442(10)	171.6(3)	82.95(3)	96.6(3)	47.2(6)
**1i** ^d^	2.803(4)	2.046(5)	2.112(3)	1.727(6)	1.429(8)	169.5(2)	76.6(2)	96.0(2)	58.7(5)
	2.758(3)	2.057(5)	2.119(3)	1.738(6)	1.432(8)	84.5(2)	177.8(2)	97.0(2)	44.0(5)
**2** [26]	2.484(3)	2.056(3)	2.134(3)	1.736(3)	1.439(4)	169.2(1)	72.5(1)	97.0(1)	2.9(3)
**3** [28]	2.695	2.050	2.119	N/A	1.449	168.0	68.8	99.9	4.8
**4** [24]	4.062	2.056	2.094	1.709	1.457	92.6	168.4	96.8	42.9

^a^Standard bond lengths: P=C 1.66 Å, P–C 1.87 Å, C=C 1.34 Å, C–C 1.46 Å, C_Ar_–I(I) 2.095 Å, C_Ar_–I(III) 2.0–2.1 Å (diaryliodonium salts), C(sp^3^)–I(I) 2.162 Å, C(sp^3^)–I(III) 2.21–2.22 Å [22,29–30]. ^b^The I–X bond length *a* is measured from I to the closest O in the nitrate anion. θ_1_ and θ_2_ are reported as the C–I–N bond angles. ^c^Two different solvatomorphs were obtained (**1f-hydrate1** and **1f-hydrate2**, see Supporting Information File 2); bond length and angle data for both solvatomorphs are given in the table. ^d^Two isomers exist in the unit cell of **1i**, with X^–^ axial (θ_1_ = 169.5) in one of them and equatorial (θ_2_ = 177.8) in the other; bond length and angle data for both isomers are given in Table 1.

All compounds show a trigonal bipyramidal structure, in which the 3-centre-4-electron bond is slightly distorted from linear geometry by 5–20° (Table 1). The short C–P and C–C bonds (*d* and *e*) in the phosphorus ylid moiety confirm Moriarty and Zhdankin’s observation that the ylid exists mainly in its enolate form (Figure 1B) [24–25] to stabilise the build-up of negative charge on this substituent in the hypervalent bond. The long I–X distances (*a* = 2.758–4.165 Å) are indicative of ionic compounds, with the exception of the cyclic benziodoxolone **2**, in which a covalent I–O bond is observed (*a* = 2.484 Å). We were unable to obtain a crystal structure of the *ortho*-nitro compound **1j**. However, a previously reported crystal structure of ylid **3**, which also contains an *ortho*-nitrobenzene substituent, suggests a pseudocyclic structure where the nitro group is coordinating to the iodine centre (*a*_(I–ON)_ = 2.695 Å) [28], which we propose is likely to be the case in **1j** as well.

In the acyclic tetrafluoroborate (**1a**), triflate (**1b**), chloride (**1c**) and nitrate (**1d**), the anion X^–^ occupies the position *trans* to the arene substituent, with the ylid in the equatorial position. In tosylates **1e** and **1f**, as well as the cyclic and pseudocyclic structures **2** and **3**, the anion or coordinating ligand occupies the position *trans* to the ylid substituent, with the aryl substituent in the equatorial position. Substituents on hypervalent iodine compounds can interconvert via Barry pseudorotation [31] and, interestingly, the crystal structure for compound **1i** contains two isomers in its unit cell, with the tosylate *trans* to the arene in one (θ_2_ = 177.8) and *trans* to the ylid in the other (θ_1_ = 169.5), which suggests that this isomerisation is fast at room temperature and the position of the anion has no significant effect on the stability of these compounds. This hypothesis is further supported by the absence of a *trans* effect. While studies by Ochiai and Suresh found that strong sigma donors X cause a lengthening and weakening of the *trans* I–R bond in R–I(Ar)–X iodanes [32–33], little variation is observed in the I–C(ylid) (*b*) and I–C(arene) (*c*) bonds across the range of compounds investigated in our study (Table 1).

### Thermal stability data

**Figure 3:**
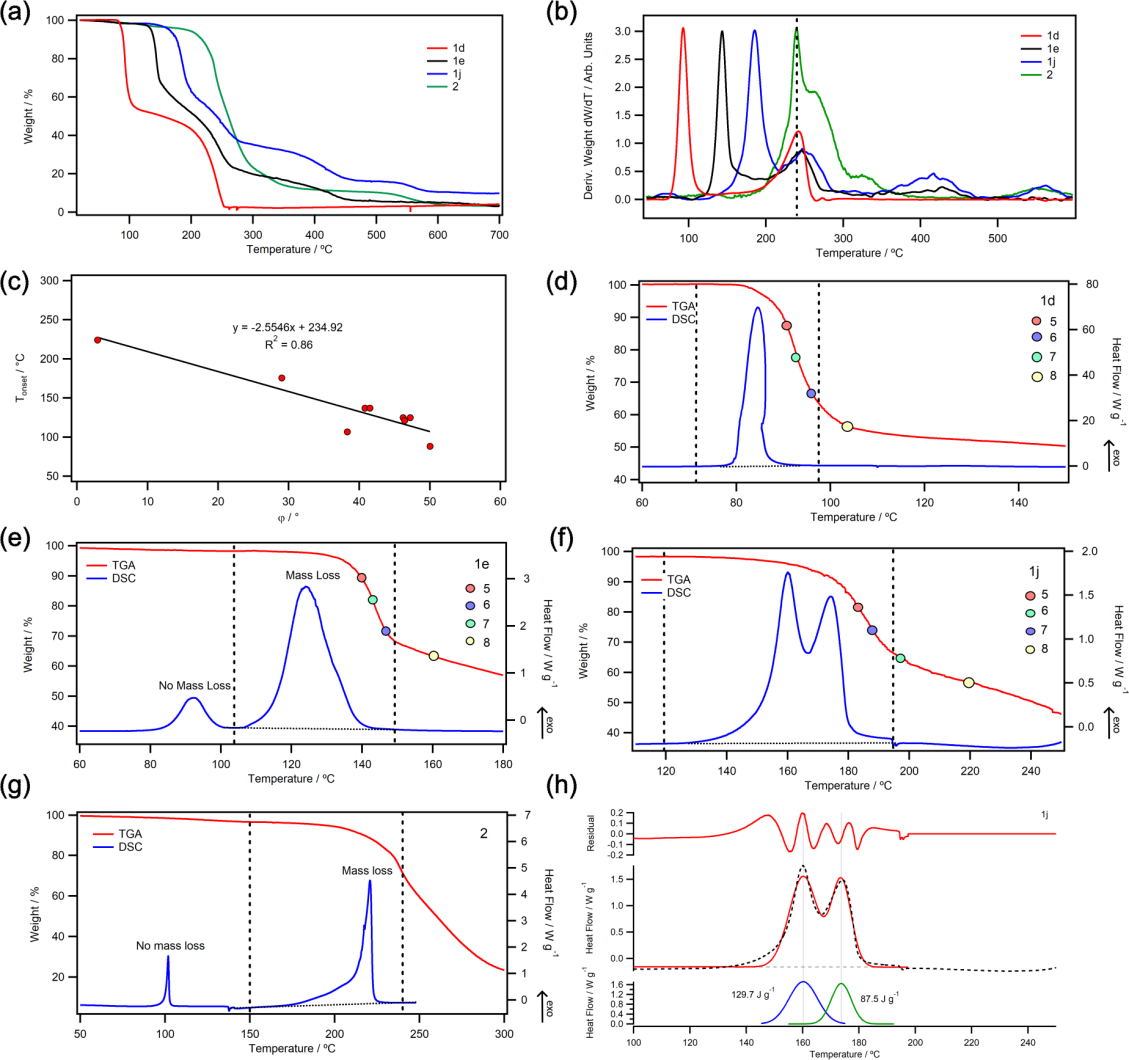
(a) Selected TGA thermograms of phosphorus-iodonium ylids at 10 °C min^−1^ in N_2_ (full dataset in Supporting Information File 2). (b) First derivatives of TGA thermograms normalised to the intensity of the first peak. (c) Correlation of *T*_onset_ with the dihedral angle φ (between the R–I–X bond and the plane of the arene substituent). (d–g) DSC thermograms overlaid with TGA thermograms with integration windows (dashed lines). Points 5–8 on the TGA thermogram denote calculated weight % values for the decomposition products **5**–**8** shown in Figure 4a. (h) Enthalpy deconvolution in DSC data of **1j** by Gaussian fit.

The phosphorus-iodonium ylids were analysed by differential scanning calorimetry (DSC) and thermogravimetric analysis (TGA) [34–35], and results have been summarised in Table 2 and Figure 3. (The full dataset is available in Supporting Information File 2.) All compounds show a multi-step mass loss behaviour with a range of TGA decomposition onset temperatures (*T*_onset_) between 107–137 °C, with the exception of three compounds that are stable to higher temperatures (**1a**: *T*_onset_ = 176 °C, Δ*H* = 134 J/g; **1j**: 172 °C, 130 J/g; **2**: 225 °C, 274 J/g; Table 2) and two highly unstable compounds (**1d**: 88 °C, 671 J/g; **1k**: 74 °C, 70.2 J/g; Table 2).

**Table 2:** DSC and TGA data^a^.

	DSC					TGA	

	Peaks^b^	Mass loss?^c^	*T*_onset_	*T*_peak_	Enthalpy	*T*_onset_	Steps
			[°C]	[°C]	Δ*H* [J/g]	[°C]	

**1a**	1	N	151.39	159.42	104.61	175.68	3
	2	Y	182.30	186.56	134.15		
**1b**	u/r	Y	59.53	88.36	179.81	106.65	4–6
**1c**	1^d^		129.31	104.35	9.89	137.17	2–4
**1d**	1		79.51	84.66	671.31	88.23	2
**1e**	1	N	85.64	91.84	39.95	137.01	3
	2	Y	114.48	124.06	267.18		
**1f**	1	Y	115.16	121.31	273.83	124.93	4–5
**1g**	1	Y	101.98	110.95	268.36	118.07	4
**1h**	1	Y	106.21	118.93	167.70	122.81	4–5
	2	Y	121.95	131.75	78.30		
**1i**	1	Y	91.13	109.50	184.10	121.14	4
	2	Y	115.65	127.01	41.10		
**1j** ^e^	1	Y	152.52	160.16	129.70	172.10	4
	2	Y	165.89	173.80	87.50		
**1k** ^f^	1		52.68	71.92	70.18	73.62	4
**2**	1	N	100.41	101.88	22.88	224.08	1–3
	2	Y	214.56	220.84	273.90		

^a^Heating rates (DSC and TGA): 10 °C min^−1^. ^b^u/r = unresolved. ^c^Mass loss is taken as <99% mass in the TGA at *T*_peak_ in the DSC. ^d^The thermogram shows a range of complex peaks after this first peak. ^e^A glass transition temperature of *T*_g_ = 32.24 °C was observed. ^f^Heating rate (DSC and TGA): 5 °C min^−1^.

We were interested to investigate whether we could identify structural features that could explain these outliers. We observed a quantifiable anion (X) effect, with acyclic tetrafluoroborate **1a** showing greater stability than the other acyclic phosphorus-iodonium ylids, while nitrate **1d** was highly thermally labile [36]. However, we were unable to rationalise or predict the anion effect with parameters such as the anion’s donor ability σ_m_, which has previously been used as a measure of its *trans* effect (Supporting Information File 2, Figure S6), the Kamlet–Taft hydrogen bond acceptor ability (β) (Supporting Information File 2, Figure S7) [37], the p*K*_a_ of the conjugate acid HX (Figure S8), or the anion’s position (axial vs equatorial).

The main stabilising factor we identified was the torsion angle φ between the hypervalent R–I–X bond and the plane of the arene substituent (Figure 3c): When the plane of the arene ring was parallel to the R–I–X bond (φ < 5°), relatively stable compounds ensued, while a large twist away from planarity resulted in compounds that were destabilised towards thermal decomposition. For example, compound **1d**, which was extremely thermally labile, had the largest dihedral angle (φ = 50°), i.e., the strongest twist away from planarity, while **1a** had the smallest dihedral angle (φ = 29°) of the acyclic compounds and showed the highest stability. In benziodoxolone **2** (φ = 3°) and pseudocyclic **1j** (cf. φ (**3**) = 5°) [28]**,** the arene ring is virtually in the same plane as the 3-centre-4-electron bond, giving rise to the most stable compounds in our series (*T*_onset_ (**2**) = 225 °C; *T*_onset_ (**1j**) = 172 °C). By contrast, mesityl phosphonium ylid **1k** showed very low thermal stability (*T*_onset_ = 74 °C, Δ*H* = 70 J/g). *Ortho*-methyl groups have been shown to destabilise iodonium species by inducing a larger hypervalent twist [31,38–39], and while we were unable to obtain a crystal structure of **1k**, a large dihedral angle (φ = 43°) was observed in Moriarty’s *ortho*-methylbenzene phosphonium ylid **4** [24], which is structurally similar.

While most DSC thermograms of our phosphorus-iodonium ylids showed single exothermic peaks during the first step of thermal decomposition (Figure 3d), some samples (**1a**, **1e**, **2**) had exothermic peaks without mass loss (Figure 3e, 3g). It is possible that these peaks correspond to a geometric rearrangement, e.g., Berry pseudorotation, which occurs prior to decomposition [31]. A large dihedral angle φ is thought to facilitate this rearrangement, thus accelerating decomposition [38–39].

### Decomposition mechanism

Further analysis was carried out to gain a better understanding of the decomposition mechanism.

**Figure 4:**
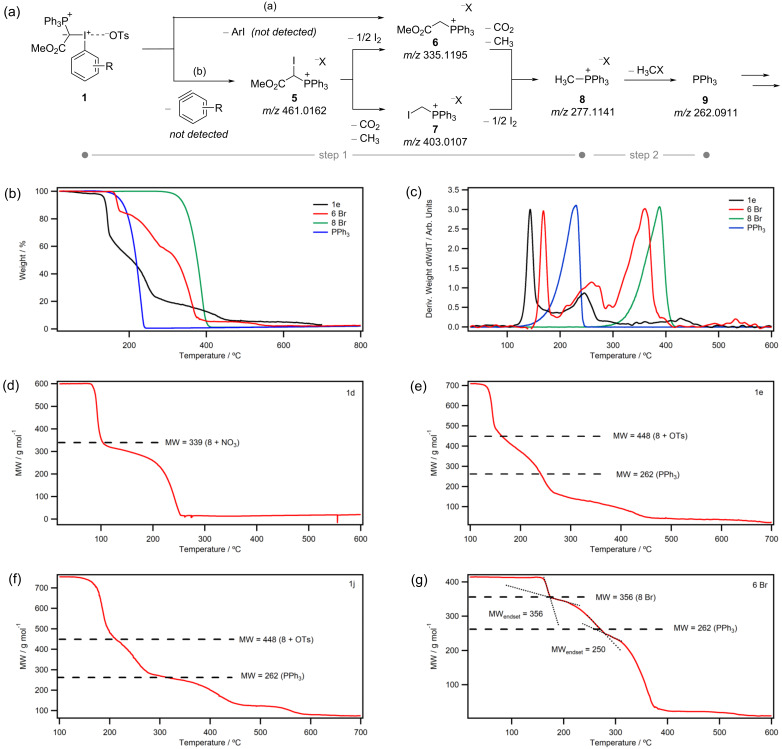
(a) Proposed decomposition mechanism based on ex situ analysis (^1^H, ^31^P NMR, ESI-MS) following aborted TGA experiments. (b–c) TGA and derivative plots for **1e** and commercial samples of decomposition products **6** (Br^−^ salt), **8** (Br^−^ salt) and PPh_3_. (d–g) TGA thermograms replotted to show the molecular weight as a function of temperature assuming 100% conversion (i.e., constant moles) with decomposition product marked.

Despite large differences in *T*_onset_, most samples showed relatively consistent second decomposition steps at ca. 225 °C (Figure 3b), which is indicative of a common decomposition intermediate for all compounds. To investigate this common intermediate, ex-situ mass spectrometry (MS) and NMR analysis were carried out on aborted TGA runs of compounds **1e**, **1i**, **1j** and **1k** that had been held at a constant temperature *T*_1_ (50–140 °C, see Supporting Information File 2) under an N_2_ atmosphere in open pans for 30 min or until a mass loss >5% of the original mass was observed, then heated to temperature *T*_2_ (136–205 °C, see Supporting Information File 2) until 20–40% mass loss of original weight. Based on this data, the following decomposition mechanism is proposed (Figure 4a):

MS, ^1^H and ^31^P NMR analysis after heating to *T*_1_ showed the presence of (methyloxycarbonylmethyl)triphenylphosphonium salt **6** and (iodomethyl)triphenyl-phosphonium salt **7**. Homolytic or heterolytic scission of the I–C(ylid) bond with loss of ArI (path a) would result in a carbene, though it is unclear how this would transform to compound **6**. Alternatively, scission of the I–C(Ar) bond with loss of benzyne (path b) results in (methoxycarbonyl(iodo)methyl)triphenylphosphonium salt **5** (observed by MS). Deiodination or decarboxylation from this intermediate afford **6** and **7**, respectively. After heating to *T*_2_, (methyl)triphenylphosphonium salt **8** is observed, which may be formed from **6** and **7** by decarboxylation and loss of iodine, respectively. Decomposition products **5**–**8** have been marked on the TGA thermograms in Figure 3d–f. All points fall within the first mass loss step, suggesting that decomposition to compound **8** occurs in a single TGA step (labelled ‘step 1’ in Figure 4b–c). (Note that a single TGA step does not necessarily correspond to one elementary reaction [40].) This is further confirmed by Figure 4d–g, which show that calculated molecular weights of the (methyl)triphenylphosphonium salts **8** coincide with the end of the first TGA step. Compound **8** likely has compromised stability from other residual decomposition products and the second TGA step represents further decomposition of **8** to PPh_3_ or a similar molecular weight salt (Figure 4a, step 2).

To confirm our mechanistic proposal for decomposition step 1, a commercial sample of (methyloxycarbonylmethyl)triphenylphosphonium bromide **6** was analysed by TGA (Figure 4b,c). The thermogram showed three decomposition steps, with a *T*_onset_ value of 164 °C. The molecular weight plot shows an endset value corresponding to the molecular weight of **8** after the first TGA step (Figure 4g), supporting the proposition that **6** decomposed to **8**.

Importantly, DSC data captures the enthalpy of decomposition for TGA step 1 only (decomposition of phosphorus-iodonium ylids **1** and **2** to (methyl)triphenylphosphonium salt **8**). This suggests that differences in the enthalpy of decomposition Δ*H* arise mainly due to difference in the aryl substituent and the anion.

When analysing *para*-substituted phosphoranyl(aryl)iodonium compounds **1f–i**, no direct correlation between the substituents’ Hammett parameter σ_p_ and the decomposition onset temperature *T*_onset_ was observed (Supporting Information File 2, Figure S13). However, the DSC thermograms of iodonium ylids with electron-poor aryl substituents (**1h**,**i**) showed two exothermic peaks (Figure 3f), which suggests that two competing decomposition pathways may be occurring in these compounds, complicating the data and any correlations drawn from it. While DSC decomposition enthalpies of both steps can be deconvoluted (Figure 3h), further studies are necessary to fully understand the electronic effect of the arene substituent.

## Conclusion

A systematic investigation of phosphorus-iodonium ylids was carried out, correlating structural data from X-ray crystallography with thermal stability data from DSC and TGA measurements.

A common decomposition mechanism involving scission of the C(ylid)–I bond or the C(Ar)–I bond was proposed based on ex situ MS and NMR analysis, resulting in the formation of (methyl)triphenylphosphonium intermediate **8**. The nature of the arene substituent (I–Ar) and anion (X) appear to play an important, yet currently unquantifiable, role in this decomposition, which will be elucidated with future computational studies.

It was, however, found that the torsion angle φ (or ‘hypervalent twist’) between the plane of the arene substituent and the hypervalent 3-centre-4-electron bond was instrumental: When the arene ring was locked in the same plane as the R–I–X bond through formation of a cyclic or pseudocyclic structure (φ < 5°), relatively stable compounds ensued, while a large twist away from planarity resulted in compounds that were destabilised towards thermal decomposition.

We envisage that the insights gained from this study will stimulate the design and synthesis of new hypervalent iodine compounds, expanding the functionalisation reactions currently available through these useful reagents in organic synthesis.

## Supporting Information

File 1 Experimental procedures, analytical data (NMR spectra), thermal (DSC, TGA) and structural (XRD) data.

File 2 X-ray structure data files.

## Data Availability

All data that supports the findings of this study is available in the published article and/or the supporting information of this article.
