# Supplementary material for: Structure and thermal stability of phosphorus-iodonium ylids
Source: Beilstein J Org Chem. 2024 Nov 14;20:2931–9. doi: 10.3762/bjoc.20.245 (PMC11571945; doi:10.3762/bjoc.20.245)
Supplement: File 1 — Experimental procedures, analytical data (NMR spectra), thermal (DSC, TGA) and structural (XRD) data. [file Beilstein_J_Org_Chem-20-2931-s001.pdf]

BEILSTEIN JOURNAL OF ORGANIC CHEMISTRY

Supporting Information

for

Structure and thermal stability of phosphorus-iodonium ylids

Andrew Greener, Stephen P. Argent, Coby J. Clarke and Miriam L. O'Duill

Beilstein J. Org. Chem. **2024,** *20,* 2931–2939. doi:10.3762/bjoc.20.245

Experimental procedures, analytical data (NMR spectra), thermal (DSC, TGA) and structural (XRD) data

License and Terms: This is a supporting information file under the terms of the Creative Commons Attribution License (https://creativecommons.org/licenses/by/4.0). Please note that the reuse, redistribution and reproduction in particular requires that the author(s) and source are credited and that individual graphics may be subject to special legal provisions.
The license is subject to the *Beilstein Journal of Organic Chemistry* terms and conditions: (https://www.beilstein-journals.org/bjoc/terms)

Contents

1. General Experimental Information S2
2. Synthesis of Phosphorus-Iodonium Ylids: Experimental Procedures and Analytical Data S2
3. Thermal Analysis S9
   3.1 Thermogravimetric Analysis (TGA) S9
   3.2 Differential Scanning Calorimetry (DSC) S11
   3.3 Correlation Analysis between Structural and Thermal Data S12
   3.4 Ex-Situ Analysis of Decomposition Products S15
4. XRD Data S17
   4.1 General Method S17
   4.2 Single Crystal X-Ray Diffraction Experimental Parameters: Summary S18
   4.3 Refinement Details S19
5. Copies of NMR Spectra S24
6. References S54

1. General Experimental Information

All reactions were performed in oven dried apparatus with magnetic stirring under an inert atmosphere of argon. All solvents and reagents were obtained from commercial suppliers and used as provided, unless stated otherwise. Solvents were dried on a column of alumina prior to use.

All NMR spectra were recorded on a Bruker AV 400 MHz spectrometer. NMR data were processed using MestReNova 14.2.1 software. Proton and carbon-13 NMR spectra are reported as chemical shifts (δ) in parts per million (ppm) relative to residual undeuterated solvent peak using the Bruker internal referencing procedure (edlock). Fluorine-19 NMR spectra are referenced relative to $CFCl_3$ in $CDCl_3$. Phosphorus-31 NMR spectra are referenced relative to phosphoric acid H_3PO_4 in $CDCl_3$. Coupling constants (*J*) are reported in units of hertz (Hz) and are rounded to the nearest 0.5 Hz for ^{1}H, ^{19}F and ^{31}P NMR and the nearest 1 Hz for ^{13}C NMR. The following abbreviations are used to describe multiplets: s (singlet), d (doublet), t (triplet), q (quartet), m (multiplet), br (broad signal), a s (apparent singlet). High-resolution mass spectrometry (HRMS) measurements were carried out on Bruker micrOTOF II. Infrared spectra (ATR-FTIR) were recorded on a Perkin–Elmer 1600 FTIR instrument as solids. Known compounds have been checked against literature references and only relevant analytical data are given. As previously reported, the quaternary ylid carbon is generally not observed in the room temperature ^{13}C NMR spectra reported herein.[1]

2. Synthesis of Phosphorus-Iodonium Ylids: Experimental Procedures and Analytical Data

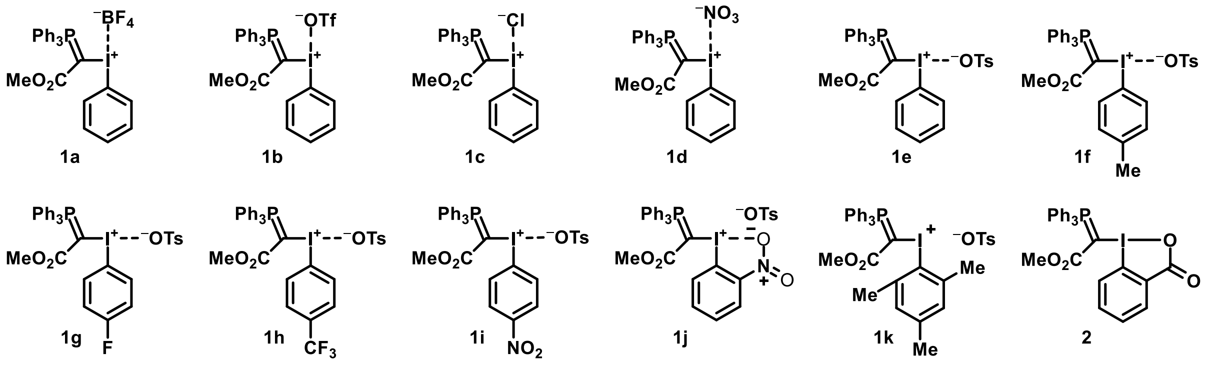

General Procedure A

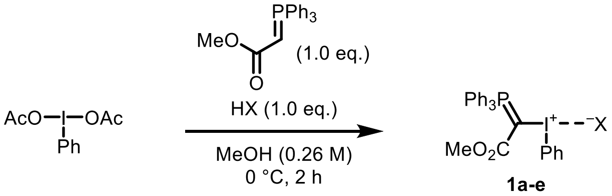

1a-e

Following a modified literature procedure,[2] phenyliodonium diacetate (PIDA) (1.0 equiv) was dissolved in MeOH (0.26 M) and cooled to 0 °C. Methyl (triphenylphospharylidene)acetate (1.0 equiv) was added followed by the acid HX (1.0 equiv), and the reaction was stirred at 0 °C for 1 h. Diethyl ether was added until a precipitate formed, and the solution was stirred at 0 °C for 1 h. The suspension was filtered and the solid washed with Et_2O and dried under vacuum to yield pure products **1a**–**e**.

Phenyliodonium(methyl (triphenylphosphoranylidene)acetate) tetrafluoroborate (1a) was prepared according to General Procedure A with phenyliodonium diacetate (PIDA) (1.0 mmol, 336 mg), methyl (triphenylphospharylidene)acetate (1.0 mmol, 334 mg) and tetrafluoro boronic acid (1.0 mmol, 0.13 mL) in MeOH (3.8 mL, 0.26 M). Precipitation with Et_2O (50 mL) afforded pure **1a** as a white solid (578 mg, 93% yield). X-ray quality single crystals were grown via vapour diffusion with MeOH/Et_2O. Analytical data matched that reported in the literature.[1]

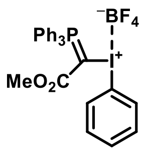

^{1}**H NMR** (400 MHz, CD_3OD) δ 7.79–7.64 (m, 3H), 7.66–7.54 (m, 15H), 7.46 (t, *J* = 7.5 Hz, 2H), 3.80 (brs, 3H); ^{13}**C NMR** (101 MHz, CD_3 OD) δ 170.1 (d, *J* = 14 Hz), 135.1 (d, *J* = 9 Hz), 135.0, 134.4, 133.0, 132.4, 130.6 (d, *J* = 13 Hz), 125.1 (d, *J* = 93 Hz), 120.3 (d, *J* = 3 Hz), 53.0; The quaternary ylid carbon was not observed.[1] ^{31}**P NMR** (162 MHz, CD_3 OD) δ_P 28.4 (s, 1P); ^{19}**F{^{1}H} NMR** (376 MHz, CD_3 OD) δ_F −154.56 (brs, $[^{10}BF_4]^-$), −154.61 (brs, $[^{11}BF_4]^-$); ^{11}**B NMR** (128 MHz, CD_3 OD) δ_B –1.1 (s, 1B); **IR** (thin film) ν_{max}/cm^{-1} 1601, 1433, 1294, 1060; **HRMS** (ESI^+) *m/z* calcd. for $C_{27}H_{23}IO_2P$ $(M)^+$ 537.0475, meas. 537.0471.

Phenyliodonium(methyl (triphenylphosphoranylidene)acetate) triflate (1b) was prepared according to General Procedure A with phenyliodonium diacetate (PIDA) (1.0 mmol, 336 mg), methyl (triphenylphospharylidene)acetate (1.0 mmol, 334 mg) and triflic acid (1.0 mmol, 0.09 mL) in MeOH (3.8 mL, 0.26 M). Precipitation with Et_2O (50 mL) afforded pure **1b** as a white solid (594 mg, 87% yield). X-ray quality single crystals were grown via vapour diffusion with $CHCl_3$/Et_2O.

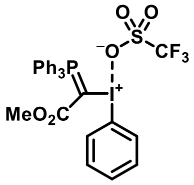

^{1}**H NMR** (400 MHz, CD_3OD) δ_H 7.79–7.75 (m, 3H), 7.66–7.54 (m, 15H), 7.46 (t, *J* = 8.0 Hz, 2H), 3.58 (brs, 3H); ^{13}**C{^{1}H} NMR** (101 MHz, CD_3OD) δ_C 170.0 (d, J_{CP} = 14 Hz), 135.1 (d, J_{CP} = 8 Hz), 135.0, 134.3, 133.0, 132.4, 130.6 (d, J_{CP} = 13 Hz), 130.0 (q, J_{CF} = 280 Hz), 125.1 (d, J_{CP} = 94 Hz), 120.3 (d, J_{CP} = 3 Hz), 53.0; The quaternary ylid carbon was not observed.[1] ^{31}**P NMR** (162 MHz, CD_3OD) δ_P 28.4 (s, 1P); ^{19}**F{^{1}H} NMR** (376 MHz, CD_3OD) δ_F –78.22 (s, 3F); **IR** (thin film) ν_{max}/cm^{-1} 1596, 1469, 1281, 1029, 732; **HRMS** (ESI^+) *m/z* calcd. for $C_{27}H_{23}IO_2P$ $(M)^+$ 537.0475, meas. 537.0469.

Phenyliodonium(methyl (triphenylphosphoranylidene)acetate) chloride (1c) was prepared according to General Procedure A with phenyliodonium diacetate (PIDA) (2.0 mmol, 644 mg), methyl (triphenylphospharylidene)acetate (2.0 mmol, 668 mg) and hydrochloric acid solution in diethyl ether (2.0 M, 2.0 mmol, 1.0 mL) in MeOH (7.6 mL, 0.26 M). Precipitation with Et_2O (80 mL) afforded pure **1c** as a white solid (806 mg, 70% yield). X-ray quality single crystals were grown via vapour diffusion with MeOH/Et_2O.

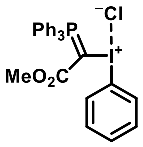

^{1}**H NMR** (400 MHz, CD_3OD) δ_H δ 7.79–7.74 (m, 3H), 7.66–7.54 (m, 15H), 7.49–7.44 (m, 2H), 3.57 (brs, 3H); ^{13}**C{^{1}H} NMR** (101 MHz, CD_3OD) δ_C 170.1 (d, J_{CP} = 14 Hz), 135.1 (d, J_{CP} = 11 Hz), 135.0, 134.3, 132.9, 132.3, 130.6 (d, J_{CP} = 13 Hz), 125.1 (d, J_{CP} = 95 Hz), 120.4 (d, *J* = 3 Hz), 53.0; The quaternary ylid carbon was not observed.[1] ^{31}**P NMR** (162 MHz, CD_3OD) δ_P 28.3 (s, 1P); **IR** (thin film) ν_{max}/cm^{-1} 1604, 1434, 1281, 1103, 979, 687; **HRMS** (ESI^+) *m/z* calcd. for $C_{27}H_{23}IO_2P$ $(M)^+$ 537.0475, meas. 537.0476.

Phenyliodonium(methyl (triphenylphosphoranylidene)acetate) nitrate (1d) was prepared according to General Procedure A with phenyliodonium diacetate (PIDA) (2.0 mmol, 644 mg), methyl (triphenylphospharylidene)acetate (2.0 mmol, 668 mg) and nitric acid (70%, 2.0 mmol, 0.13 mL) in MeOH (7.6 mL, 0.26 M). Precipitation with Et_2O (80 mL) afforded pure **1d** as a white solid (1003 mg, 84% yield). X-ray quality single crystals were grown via vapour diffusion with MeOH/Et_2O. [Caution: The decomposition of **1d** is highly exothermal and great care must be taken when preparing and handling this compound.]

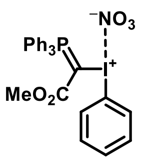

^{1}H NMR (400 MHz, CD_3OD) δ_H 7.79–7.74 (m, 3H), 7.67–7.54 (m, 15H), 7.49–7.44 (m, 2H), 3.58 (brs, 3H); **^{13}C{^{1}H} NMR** (101 MHz, CD_3OD) δ_C 170.1 (d, J_{CP} = 14 Hz), 135.1 (d, J_{CP} = 11 Hz), 135.0, 134.3, 132.9, 132.3, 130.6 (d, J_{CP} = 13 Hz), 125.1 (d, J_{CP} = 95 Hz), 120.4 (d, J_{CP} = 3 Hz), 53.0; The quaternary ylid carbon was not observed.[1] **^{31}P NMR** (162 MHz, CD_3OD) δ_P 28.4 (s, 1P); **IR** (thin film) ν_{max}/cm^{-1} 1604, 1434, 1281, 1103, 979, 687; **HRMS** (ESI$^+$) *m/z* calcd. for $C_{27}H_{23}IO_2P$ (M)$^+$ 537.0475, meas. 537.0476.

Phenyliodonium(methyl (triphenylphosphoranylidene)acetate) tosylate (1e) was prepared according to General Procedure A with phenyliodonium diacetate (PIDA) (1.0 mmol, 336 mg), methyl (triphenylphospharylidene)acetate (1.0 mmol, 334 mg) and tosic acid (1.0 mmol, 190 mg) in MeOH (3.8 mL, 0.26 M). Precipitation with Et_2O (50 mL) afforded pure **1e** as a white solid (652 mg, 92% yield). X-ray quality single crystals were grown via vapour diffusion with $CHCl_3/Et_2O$.

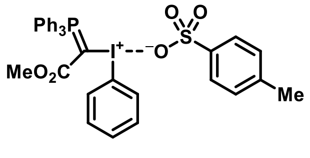

^{1}H NMR (400 MHz, CD_3OD) δ_H 7.79–7.74 (m, 3H), 7.70 (d, J = 8.0 Hz, 2H), 7.66–7.53 (m, 15H), 7.46 (t, J = 7.5 Hz, 2H), 7.21 (d, J = 8.0 Hz, 2H), 3.57 (brs, 3H), 2.36 (s, 3H); **^{13}C{^{1}H} NMR** (101 MHz, CD_3OD) δ_C 169.8 (d, J_{CP} = 14 Hz), 143.8, 141.3, 134.9 (d, J_{CP} = 6 Hz), 134.8, 134.1, 132.8, 132.2, 130.5 (d, J_{CP} = 13 Hz), 129.7, 126.9, 124.9 (d, J_{CP} = 94 Hz), 120.2 (d, J_{CP} = 4 Hz), 53.0, 21.4; The quaternary ylid carbon was not observed.[1] **^{31}P NMR** (162 MHz, CD_3OD) δ_P 28.5 (s, 1P); **IR** (thin film) ν_{max}/cm^{-1} 1618, 1433, 1283, 1176, 1099, 674; **HRMS** (ESI$^+$) *m/z* calcd. for $C_{27}H_{23}IO_2P$ (M)$^+$ 537.0475, meas. 537.0470.

General Procedures B and C

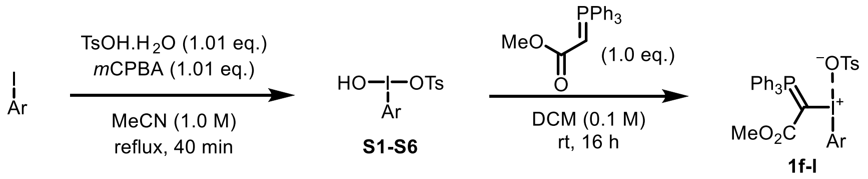

General Procedure B: Following a modified literature procedure,[3] aryl iodide (1.0 equiv) and tosic acid monohydrate (1.01 equiv) were suspended in MeCN (1.0 M) and *meta*-chloroperbenzoic acid (*m*CPBA) (1.01 equiv) was added. The suspension was heated to reflux and stirred for 40 min before being allowed to cool to room temperature. Diethyl ether was added until a precipitate formed, and the suspension was filtered and the solid washed Et_2O and then dried under vacuum to yield the products **S1**–**S6**.

General Procedure C: Following a modified literature procedure,[4] hydroxy(tosyloxy)arenes **S1–S6** (1.0 equiv) and methyl (triphenylphospharylidene)acetate (1.0 equiv) were dissolved in DCM (0.1 M) and the reaction stirred at room temperature for 16 h. The solution was concentrated under vacuum and the residue was dissolved in minimal DCM before diethyl ether was added until a precipitate formed. The suspension was filtered and the solid washed with diethyl ether and then dried under vacuum to yield the products **1f–l**.

1-(Hydroxy(tosyloxy)iodo)-4-methylbenzene (S1) was prepared according to General Procedure B with 4-iodomethylbenzene (2.00 mmol, 436 mg), *m*CPBA (2.02 mmol, 349 mg) and tosic acid monohydrate (2.02 mmol, 384 mg) in MeCN (2.0 mL, 1.0 M). Precipitation with Et_2O (80 mL) afforded pure **S1** as a white solid (692 mg, 85% yield). Analytical data matched that reported in literature.[5]

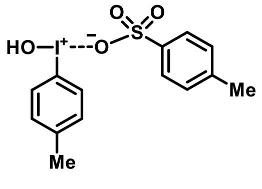

^{1}H NMR (400 MHz, CD_3OD) δ_H 8.21 (d, J = 8.0 Hz, 2H), 7.67 (d, J = 8.0 Hz, 2H), 7.49 (d, J = 8.0 Hz, 2H), 7.22 (d, J = 8.0 Hz, 2H), 2.50 (s, 3H), 2.37 (s, 3H); **^{13}C{^{1}H} NMR** (101 MHz, CD_3OD) δ_C 146.9, 143.2, 141.8, 137.4, 133.5, 129.8, 126.9, 118.6, 21.7, 21.3.

4-Methylphenyliodonium(methyl (triphenylphosphoranylidene)acetate) tosylate (1f) was prepared according to General Procedure C with 1-(hydroxy-(tosyloxy)iodo)-4-methylbenzene **S1** (1.0 mmol, 406 mg) and methyl (triphenyl-phospharylidene) acetate (1.0 mmol, 334 mg) in DCM (10 mL, 0.1 M). Precipitation with Et_2O (50 mL) afforded pure **1f** as a white solid (644 mg, 89% yield). X-ray quality single crystals were grown via vapour diffusion with MeOH/Et_2O. Two different solvatomorphs (**1f-hydrate1** and **1f-hydrate2**) were obtained.

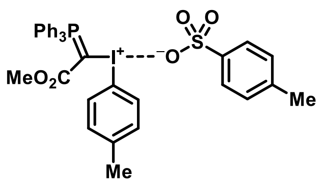

^{1}H NMR (400 MHz, CD_3OD) δ_H 7.77–7.73 (m, 3H), 7.71–7.69 (m, 2H), 7.60–7.56 (m, 12H), 7.44 (d, *J* = 8.0 Hz, 2H), 7.27 (d, *J* = 7.5 Hz, 2H), 7.20 (d, *J* = 7.5 Hz, 2H), 3.58 (brs, 3H), 2.42 (s, 3H), 2.34 (s, 3H); **^{13}C{^{1}H} NMR** (101 MHz, CD_3OD) δ_C 170.1 (d, J_{CP} = 14 Hz), 144.2, 143.7, 141.5, 135.1 (d, J_{CP} = 11 Hz), 135.0, 134.4, 133.0, 130.6 (d, J_{CP} = 13 Hz), 129.8, 127.0, 125.2 (d, J_{CP} = 92 Hz), 116.7, 53.0, 21.3; The quaternary ylid carbon was not observed.[1] **^{31}P NMR** (162 MHz, CD_3OD) δ_P 28.4 (s, 1P); **IR** (thin film) ν_{max}/cm^{-1} 3059, 1591, 1434, 1170, 1102, 1001, 677; **HRMS** (ESI$^+$) *m/z* calcd. for $C_{27}H_{22}FIO_2P$ $(M)^+$ 555.0381, meas. 555.0378.

1-(Hydroxy(tosyloxy)iodo)-4-fluorobenzene (S2) was prepared according to General Procedure B with 4-fluoroiodobenzene (2.00 mmol, 444 mg), *m*CPBA (2.02 mmol, 349 mg) and tosic acid monohydrate (2.02 mmol, 384 mg) in MeCN (2.0 mL, 1.0 M). Precipitation with Et_2O (80 mL) afforded pure **S2** as a white solid (598 mg, 73% yield).

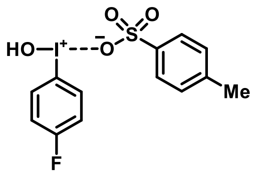

^{1}H NMR (400 MHz, CD_3OD) δ_H 8.39 (dd, *J* = 8.5, 5.0 Hz, 2H), 7.66 (d, *J* = 8.0 Hz, 2H), 7.41 (dd, *J* = 8.5 Hz, 2H), 7.22 (d, *J* = 8.0 Hz, 2H), 2.36 (s, 3H); **^{13}C{^{1}H} NMR** (101 MHz, CD_3OD) δ_C 166.9 (d, J_{CF} = 255 Hz), 143.0, 141.9, 140.3 (d, J_{CF} = 9 Hz), 129.8, 126.9, 120.2 (d, J_{CF} = 23 Hz), 116.5, 21.2; **^{19}F{^{1}H} NMR** (376 MHz, CD_3OD) δ_F –104.52 (s, 1F).

4-Fluoromethylphenyliodonium(methyl (triphenylphosphoranylidene)acetate) tosylate (1g) was prepared according to General Procedure C with 1-(hydroxy(tosyloxy)iodo)-4-fluorobenzene **S2** (1.0 mmol, 410 mg) and methyl (triphenylphospharylidene)acetate (1.0 mmol, 334 mg) in DCM (10 mL, 0.1 M). Precipitation with Et_2O (50 mL) afforded pure **1g** as a white solid (518 mg, 80% yield).

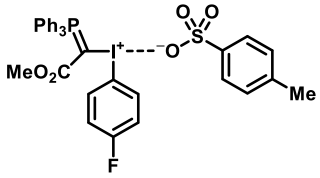

^{1}H NMR (400 MHz, CD_3OD) δ_H 7.79–7.75 (m, 3H), 7.71 (d, *J* = 8.0 Hz, 2H), 7.64–7.57 (m, 14H), 7.26–7.21 (m, 4H), 3.59 (brs, 3H), 2.36 (s, 3H); **^{13}C{^{1}H} NMR** (101 MHz, CD_3OD) δ_C 169.9 (d, J_{CP} = 14 Hz), 165.8 (d, J_{CF} = 252 Hz), 143.7, 141.5, 137.0, 135.0, 134.9 (d, J_{CP} = 6 Hz), 130.6 (d, J_{CP} = 13 Hz), 129.7, 126.9, 125.0 (d, J_{CP} = 95 Hz), 119.5 (d, J_{CF} = 23 Hz), 114.1, 53.0, 21.3; The quaternary ylid carbon was not observed.[1] **^{31}P NMR** (162 MHz, CD_3OD) δ_P 28.3 (s, 1P); **^{19}F{^{1}H} NMR** (376 MHz, CD_3OD) δ_F –108.43 (s, 1F); **IR** (thin film) ν_{max}/cm^{-1} 1627, 1480, 1434, 1221, 1176, 1008, 677; **HRMS** (ESI$^+$) *m/z* calcd. for $C_{27}H_{22}FIO_2P$ $(M)^+$ 555.0381, meas. 555.0378.

1-(Hydroxy(tosyloxy)iodo)-4-trifluoromethylbenzene (S3) was prepared according to General Procedure B with 4-iodotrifluoromethylbenzene (2.00 mmol, 544 mg), *m*CPBA (2.02 mmol, 349 mg) and tosic acid monohydrate (2.02 mmol, 384 mg) in MeCN (2.0 mL, 1.0 M). Precipitation with Et_2O (80 mL) afforded pure **S3** as a white solid (883 mg, 96%). Analytical data matched that reported in literature.[5]

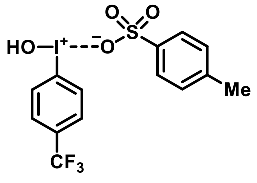

^{1}H NMR (400 MHz, CD_3OD) δ_H 8.47 (d, *J* = 8.0 Hz, 2H), 7.92 (d, *J* = 8.0 Hz, 2H), 7.62 (d, *J* = 8.0 Hz, 2H), 7.20 (d, *J* = 8.0 Hz, 2H), 2.35 (s, 3H); **^{13}C{^{1}H} NMR** (101 MHz, CD_3OD) δ_C 142.3, 142.1, 137.1, 135.6 (q, J_{CF} = 33 Hz), 129.9, 129.3 (q, J_{CF} = 4 Hz), 126.9, 125.6, 124.6 (q, J_{CF} = 272 Hz), 21.2; **^{19}F{^{1}H} NMR** (376 MHz, CD_3OD) δ_F –64.77 (s, 3F); **IR** (thin film) ν_{max}/cm^{-1} 3005, 1318, 1131, 999, 562.

4-Trifluoromethylphenyliodonium(methyl (triphenylphosphoranylidene)acetate) tosylate (1h) was prepared according to General Procedure C with 1-(hydroxy-(tosyloxy)iodo)-4-trifluoromethylbenzene **S3** (1.0 mmol, 460 mg) and methyl (triphenylphospharylidene)acetate (1.0 mmol, 334 mg) in DCM (10 mL, 0.1 M). Precipitation with Et_2O (50 mL) afforded pure **1h** as a white solid (586 mg, 75% yield).

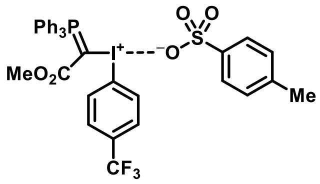

^{1}H NMR (400 MHz, CD_3OD) δ_H 7.82–7.56 (m, 21H), 7.21 (d, *J* = 8.0 Hz, 2H), 3.58 (brs, 3H), 2.35 (s, 3H); **^{13}C{^{1}H} NMR** (101 MHz, CD_3OD) δ_C 169.9 (d, J_{CP} = 13 Hz), 143.7, 141.6, 135.1, 135.1 (d, J_{CP} = 11 Hz), 134.9, 134.4 (q, J_{CF} = 33 Hz), 130.7 (d, J_{CP} = 13 Hz), 129.8, 129.0 (q, J_{CF} = 4 Hz), 127.0, 125.0 (d, J_{CP} = 93 Hz), 124.8 (q, J_{CF} = 272 Hz), 124.5 (q, J_{CF} = 3 Hz), 53.2, 21.3; The quaternary ylid carbon was not observed.[1] **^{31}P NMR** (162 MHz, CD_3OD) δ_P 28.5 (s, 1P); **^{19}F{^{1}H} NMR** (376 MHz, CD_3OD) δ_F –64.36 (s, 3F); **IR** (thin film) ν_{max}/cm^{-1} 3060, 1631, 1163, 1103, 1007, 677; **HRMS** (ESI$^+$) *m/z* calcd. for $C_{27}H_{22}FIO_2P$ $(M)^+$ 555.0381, meas. 555.0378.

1-(Hydroxy(tosyloxy)iodo)-4-nitrobenzene (S4) was prepared according to General Procedure B with 4-iodonitrobenzene (2.00 mmol, 498 mg), *m*CPBA (2.02 mmol, 349 mg) and tosic acid monohydrate (2.02 mmol, 384 mg) in MeCN (2.0 mL, 1.0 M). Precipitation with Et_2O (80 mL) afforded pure **S4** as a white solid (806 mg, 92%).

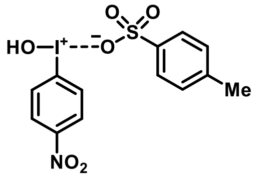

^{1}H NMR (400 MHz, CD_3OD) δ_H 8.49 (d, *J* = 8.5 Hz, 2H), 8.38 (d, *J* = 8.5 Hz, 2H), 7.61 (d, *J* = 8.0 Hz, 2H), 7.20 (d, *J* = 8.0 Hz, 2H), 2.35 (s, 3H); **^{13}C{^{1}H} NMR** (101 MHz, CD_3OD) δ_C 151.7, 142.6, 142.1, 137.5, 129.9, 127.4, 127.1, 126.9, 21.2; **IR** (thin film) ν_{max}/cm^{-1} 2955, 1529, 1350, 1202, 1112, 993, 561.

4-Nitrophenyliodonium(methyl (triphenylphosphoranylidene)acetate) tosylate (1i) was prepared according to General Procedure C with 1-(hydroxy(tosyloxy)iodo)-4-nitrobenzene **S4** (1.0 mmol, 437 mg) and methyl(triphenyl-phospharylidene) acetate (1.0 mmol, 334 mg) in DCM (10 mL, 0.1 M). Precipitation with Et_2O (50 mL) afforded pure **1i** as a white solid (666 mg, 88% yield). X-ray quality single crystals were grown via vapour diffusion with MeOH/Et_2O.

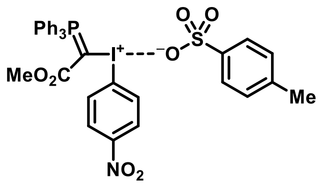

^{1}H NMR (400 MHz, CD_3OD) δ_H 8.25 (d, *J* = 8.5 Hz, 2H), 7.85 (d, *J* = 8.0 Hz, 2H), 7.78–7.74 (m, 3H), 7.67 (d, *J* = 7.0 Hz, 1H), 7.63–7.58 (m, 12H), 7.20 (d, *J* = 7.5 Hz, 2H), 3.58 (brs, 3H), 2.35 (s, 3H); **^{13}C{^{1}H} NMR** (101 MHz, CD_3OD) δ_C 169.8 (d, J_{CP} = 14 Hz), 151.1, 148.6, 141.6, 140.1, 135.2 (d, J_{CP} = 3 Hz), 135.1 (d, J_{CP} = 10 Hz), 130.7 (d, J_{CP} = 13 Hz), 129.8, 126.9, 126.8, 124.9 (d, J_{CP} = 94 Hz), 53.2, 21.3; The quaternary ylid carbon was not observed.[1] **^{31}P NMR** (162 MHz, CD_3OD) δ_P 28.6 (s, 1P); **IR** (thin film) ν_{max}/cm^{-1} 1631, 1522, 1435, 1163, 1103, 1030, 677; **HRMS** (ESI$^+$) *m/z* calcd. for $C_{27}H_{22}FIO_2P$ $(M)^+$ 555.0381, meas. 555.0378.

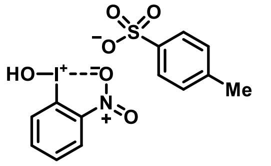

1-(Hydroxy(tosyloxy)iodo)-2-nitrobenzene (S5) was prepared according to General Procedure B with 2-iodonitrobenzene (2.00 mmol, 544 mg), *m*CPBA (2.02 mmol, 349 mg) and tosic acid monohydrate (2.02 mmol, 384 mg) in MeCN (2.0 mL, 1.0 M). Precipitation with Et_2O (80 mL) afforded pure **S5** as a white solid (601 mg, 69%). **^{1}H NMR** (400 MHz, CD_3OD) δ_H 8.60 (d, *J* = 8.0 Hz, 1H), 8.25 (dd, *J* = 8.0, 7.5 Hz, 1H), 8.19–8.15 (m, 1H), 7.98 (dd, *J* = 8.0, 7.5 Hz, 1H), 7.66 (d, *J* = 8.0 Hz, 2H), 7.21 (d, *J* = 8.0 Hz, 2H), 2.36 (s, 3H); **^{13}C{^{1}H} NMR** (101 MHz, CD_3OD) δ_C 145.3, 143.3, 141.8, 140.1, 134.2, 131.0, 129.8, 128.7, 126.9, 115.4, 21.3.

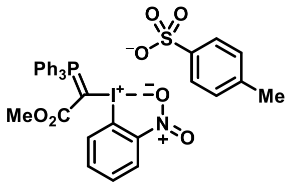

2-Nitrophenyliodonium(methyl (triphenylphosphoranylidene)acetate) tosylate (1j) was prepared according to General Procedure C with 1-(hydroxy(tosyloxy)iodo)-2-nitrobenzene **S5** (1.0 mmol, 437 mg) and methyl (triphenylphospharylidene)acetate (1.0 mmol, 334 mg) in DCM (10 mL, 0.1 M). Precipitation with Et_2O (50 mL) afforded pure **1j** as a white solid (679 mg, 90% yield).

^{1}H NMR (400 MHz, CD_3OD) δ_H 8.44 (d, *J* = 8.0 Hz, 1H), 8.20 (d, *J* = 8.0 Hz, 1H), 8.00 (t, *J* = 7.5 Hz, 1H), 7.92–7.48 (m, 18H), 7.20 (d, *J* = 8.0 Hz, 2H), 3.62 (s, 3H), 2.34 (s, 3H); **^{13}C{^{1}H} NMR** (101 MHz, CD_3OD) δ_C 165.4 (d, J_{CP} = 14 Hz), 146.4, 143.7, 141.6, 138.6, 135.4 (d, J_{CP} = 3 Hz), 135.1 (d, J_{CP} = 10 Hz), 133.7, 130.9 (d, J_{CP} = 13 Hz), 129.8, 128.5, 127.0, 124.9 (d, J_{CP} = 94 Hz), 113.9, 53.5, 21.3; The quaternary ylid carbon was not observed.[1] **^{31}P NMR** (162 MHz, CD_3OD) δ_P 29.6 (s, 1P); **IR** (thin film) ν_{max}/cm^{-1} 1638, 1521, 1435, 1163, 1107, 1030, 677; **HRMS** (ESI$^+$) *m/z* calcd. for $C_{27}H_{22}FIO_2P$ (M)$^+$ 555.0381, meas. 555.0378.

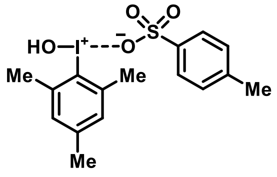

1-(Hydroxy(tosyloxy)iodo)-2,4,6-trimethylbenzene (S6) was prepared according to General Procedure B with 4-iodo-2,4,6-trimethylbenzene (2.00 mmol, 492 mg), *m*CPBA (2.02 mmol, 349 mg) and tosic acid monohydrate (2.02 mmol, 384 mg) in MeCN (2.0 mL, 1.0 M). Precipitation with Et_2O (80 mL) afforded pure **S6** as a white solid (491 mg, 57% yield).

^{1}H NMR (400 MHz, CD_3OD) δ_H 7.61 (d, *J* = 8.0 Hz, 2H), 7.28 (s, 2H), 7.20 (d, *J* = 8.0 Hz, 2H), 2.72 (s, 6H), 2.41 (s, 3H), 2.36 (s, 3H); **^{13}C{^{1}H} NMR** (101 MHz, CD_3OD) δ_C 147.5, 144.2, 143.0, 141.9, 130.6, 129.8, 128.5, 126.9, 26.7, 21.34, 21.30.

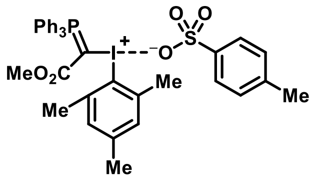

2,4,6-Trimethylphenyliodonium(methyl (triphenylphosphoranylidene)acetate) tosylate (1k) was prepared according to General Procedure C with 1-(hydroxy(tosyloxy)iodo)-2,4,6-trimethylbenzene **S6** (1.0 mmol, 434 mg) and methyl (triphenylphospharylidene)acetate (1.0 mmol, 334 mg) in DCM (10 mL, 0.1 M). Precipitation with Et_2O (50 mL) afforded pure **1k** as a white solid (560 mg, 75% yield).

^{1}H NMR (400 MHz, CD_3OD) δ_H 7.70 (d, *J* = 8.0 Hz, 2H), 7.59–7.56 (m, 15H), 7.22 (d, *J* = 8.0 Hz, 2H), 7.05 (s, 2H), 3.48 (brs, 3H), 2.36 (s, 6H), 2.13 (s, 6H); **^{13}C{^{1}H} NMR** (101 MHz, CD_3OD) δ_C 166.5, 143.5, 142.7, 141.6, 136.4 (d, J_{CP} = 3 Hz), 135.0, 133.1, 131.4, 130.0 (d, J_{CP} = 12 Hz), 129.8, 126.9, 119.4 (d, J_{CP} = 90 Hz), 104.5, 53.9, 29.7, 21.3, 20.7; The quaternary ylid carbon was not observed.[1] **^{31}P NMR** (162 MHz, CD_3OD) δ_P 26.0 (s, 1P); **IR** (thin film) ν_{max}/cm^{-1}2905, 1722, 1436, 1166, 1117, 1008, 672; **HRMS** (ESI$^+$) *m/z* calcd. for $C_{27}H_{22}FIO_2P$ (M)$^+$ 555.0381, meas. 555.0378.

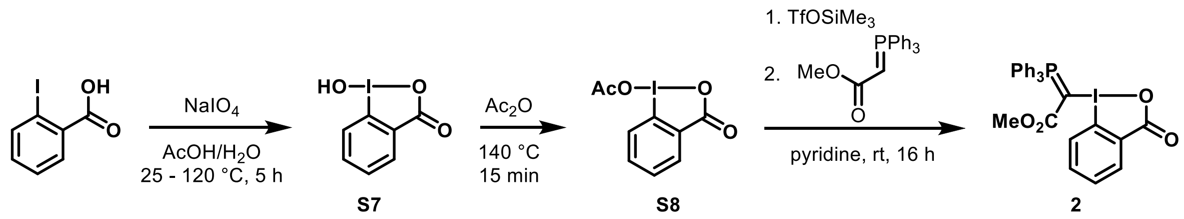

Hydroxybenziodoxolone (S7)

Following a literature procedure,[6] 2-iodobenzoic acid (1.0 equiv, 8.0 mmol, 1.98 g) and sodium periodate (1.05 equiv, 8.4 mmol, 1.80 g) were dissolved in a solution of acetic acid (30% v/v in water, 0.6 M, 12 mL). The resulting solution was heated to 120 °C and stirred vigorously for 4 h. The resulting suspension was cooled to room temperature and ice-cold water was added and the flask wrapped in foil to protect from ambient light. After being stirred for 1 h the suspension was filtered, and the resulting solid was washed with ice-cold water and acetone. The solid was air-dried whilst being protected from ambient light to yield pure **S7** as a white solid (1.99 g, 94% yield). Spectroscopic data matched that reported in the literature.[7]

^{1}H NMR (400 MHz, $(CD_3)_2SO$) δ_H 8.06 (brs, 1H, –O*H*), 8.01 (dd, *J* = 7.5, 1.5 Hz, 1H), 7.96 (ddd, *J* = 8.0, 7.0, 1.5 Hz, 1H), 7.84 (dd, *J* = 8.0, 1.0 Hz, 1H), 7.70 (ddd, *J* = 7.5, 7.0, 1.0 Hz, 1H); **^{13}C{^{1}H} NMR** (101 MHz, $(CD_3)_2SO$) δ_C 167.7, 134.5, 131.5, 131.1, 130.4, 126.3, 120.4; **HRMS** (ESI$^+$) *m/z* calcd. for $C_7H_6IO_3$ $(M + H)^+$ 264.9356, meas. 264.9355.

Acetoxybenziodoxolone (S8)

Following a literature procedure,[8] hydroxybenziodoxolone **S7** (1.0 equiv, 8.0 mmol, 2.11 g) was dissolved in neat acetic anhydride (1.4 M, 5.8 mL). The resulting solution was heated to 140 °C and stirred vigorously for 15 min. The resulting suspension was cooled to −18 °C and the excess acetic anhydride was decanted. The solid was dried under vacuum to yield pure **S8** as a white solid (2.42 g, 99% yield). Spectroscopic data matched that reported in the literature.[7]

^{1}H NMR (400 MHz, $CDCl_3$) δ_H 8.26 (dd, *J* = 7.5, 1.5 Hz, 1H), 8.01 (dd, *J* = 8.5, 1.0 Hz, 1H), 7.93 (ddd, *J* = 8.5, 7.0, 1.5 Hz, 1H), 7.72 (ddd, *J* = 7.5, 7.0, 1.0 Hz, 1H), 2.26 (s, 3H); **^{13}C{^{1}H} NMR** (75 MHz, $CDCl_3$) δ_C 176.5, 168.3, 136.3, 133.4, 131.5, 129.5, 129.2, 118.5, 20.5; **HRMS** (ESI$^+$) *m/z* calcd. for $C_9H_8IO_4$ $(M + H)^+$ 306.9462, meas. 306.9462.

Methyl (triphenylphosphoranylidenylbenziodoxolone)acetate (2)

Following a modified literature procedure,[9] acetoxybenziodoxolone **S8** (1.0 equiv, 2.0 mmol, 612 mg) and trimethylsilyl triflate (1.0 equiv 2.0 mmol, 349 mg) were dissolved in pyridine (0.2 M, 10.0 mL). The resulting solution was stirred for 2 h at room temperature. Methyl(triphenylphosphoranylidene) acetate (1.0 equiv, 2.0 mmol, 669 mg) was added, and the solution was stirred at room temperature for 16 h. The resulting suspension was diluted in DCM, filtered, and washed with a saturated $NaHCO_3$ solution. The combined organic phases were dried over $MgSO_4$ and concentrated in vacuo. The residue was recrystalised in DCM and Et_2O. The resulting material was redissolved in DCM and washed with a saturated $NaHCO_3$ solution. The DCM layer was dried over $MgSO_4$ and concentrated under vacuum to yield pure **2** as a pale pink solid (255 mg, 22% yield). Analytical data matched that reported in literature.[9]

^{1}H NMR (400 MHz, $CDCl_3$) δ_H 8.33 (dd, *J* = 7.5, 1.5 Hz, 1H), 7.79 (d, *J* = 8.0 Hz, 1H), 7.59–7.51 (m, 17H), 3.56 (s, 3H); **^{13}C{^{1}H} NMR** (101 MHz, $CDCl_3$) δ_C 169.5 (d, J_{CP} = 13 Hz), 167.3, 134.3, 133.6, 133.5 (d, J_{CP} = 10 Hz), 132.5 (d, J_{CP} = 6 Hz), 130.0, 129.5 (d, J_{CP} = 13 Hz), 125.2 (d, J_{CP} = 93 Hz), 123.1, 117.5, 52.3; The quaternary ylid carbon was not observed.[1] **^{31}P NMR** (162 MHz, $CDCl_3$) δ_P 27.2 (s, 1P); **IR** (thin film) ν_{max}/cm^{-1} 1601, 1427, 1332, 1258, 1066, 959, 743; **HRMS** (ESI$^+$) *m/z* calcd. for $C_{28}H_{23}IO_4P$ $(M + H)^+$ 581.0373, meas. 581.0368.

3. Thermal Analysis

3.1 Thermogravimetric Analysis (TGA)

Thermogravimetric analysis (TGA) experiments were carried using a TA Instruments Discovery TGA 550. Approximately 2–10 mg of sample was loaded onto a high temperature platinum pan which was heated at 10 °C min^{-1} in an N_2 atmosphere at 50 mL min^{-1}. (Note: Due to the low onset temperature of **1k**, a lower heating rate of 5 °C min^{-1} was required for this sample.) Platinum pans were cleaned in a high temperature flame between runs to burn off residual organics.

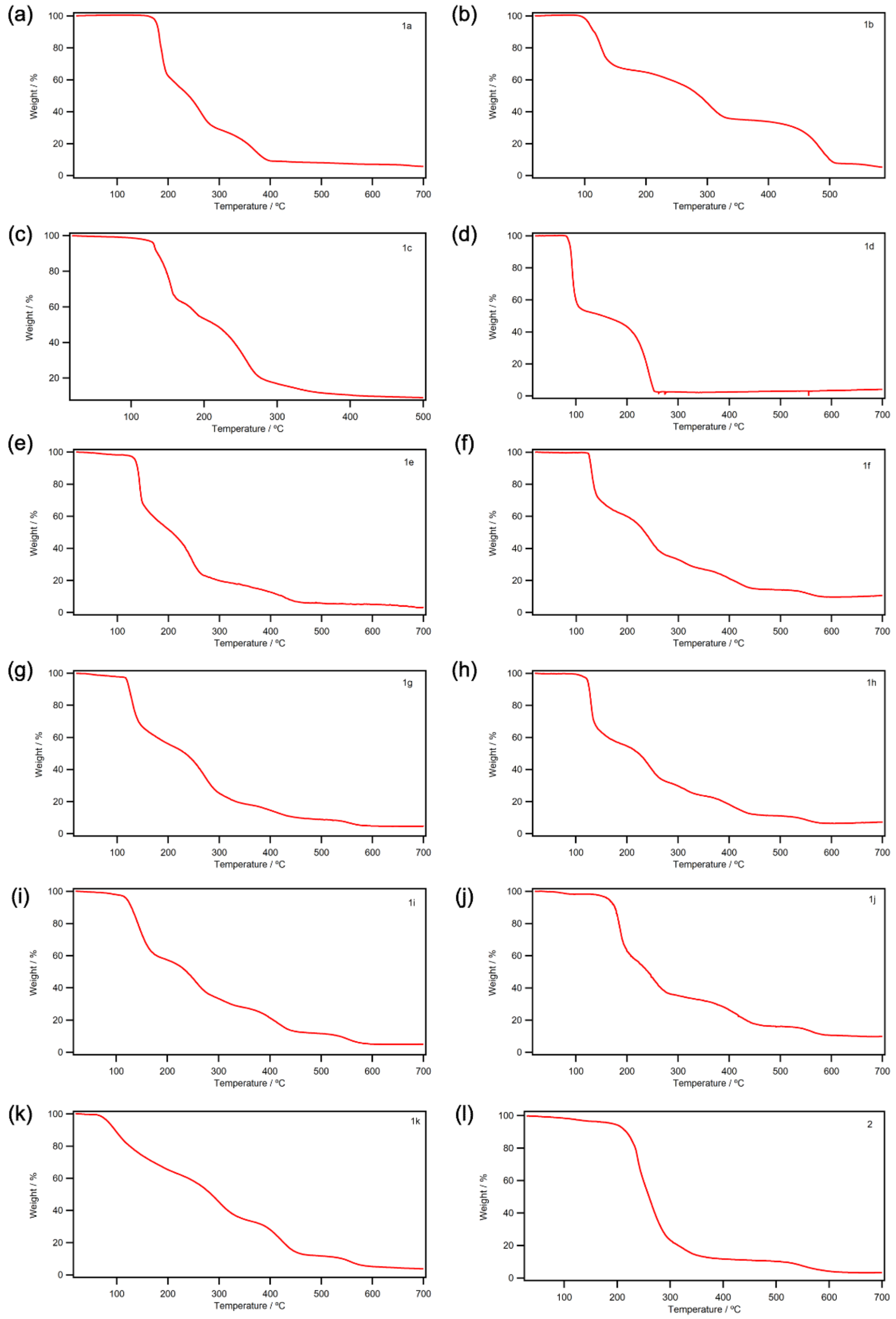

Figure S1: TGA thermograms measured at 10 °C min^{-1} in N_2 for phosphorus-iodonium ylids **1a–k**.

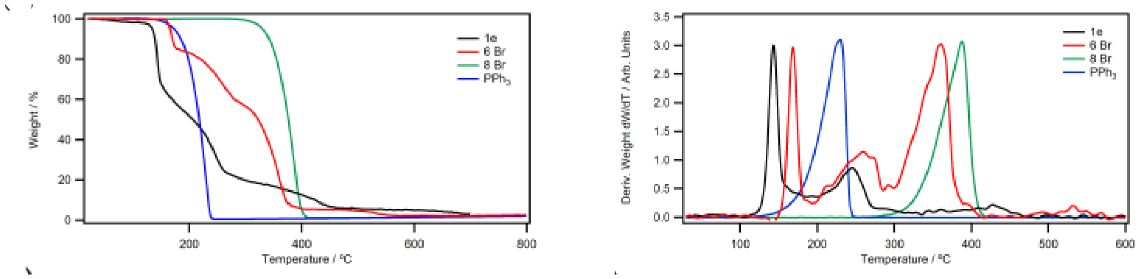

Figure S2: TGA and derivative plots for **1e** and commercial samples of (methyloxycarbonylmethyl)-triphenylphosphonium bromide **6**, (methyl)triphenylphosphonium bromide **8** and PPh_3 (10 °C min^{-1} in N_2).

3.2 Differential Scanning Calorimetry (DSC)

Differential scanning calorimetry (DSC) experiments were performed using a TA Instruments Discovery DSC2500 equipped with an RCS90 chiller. Temperature and the cell constant were calibrated before use with a high purity indium standard. Approximately 2–5 mg of each compound was sealed in individual hermetic alodined aluminium pans and samples were heated at 10 °C min^{-1} in an N_2 atmosphere at 50 mL min^{-1}. (Note: Due to the low onset temperature of **1k**, a lower heating rate of 5 °C min^{-1} was required for this sample.) Data was analysed in Trios 5.1.1 using linear baselines.

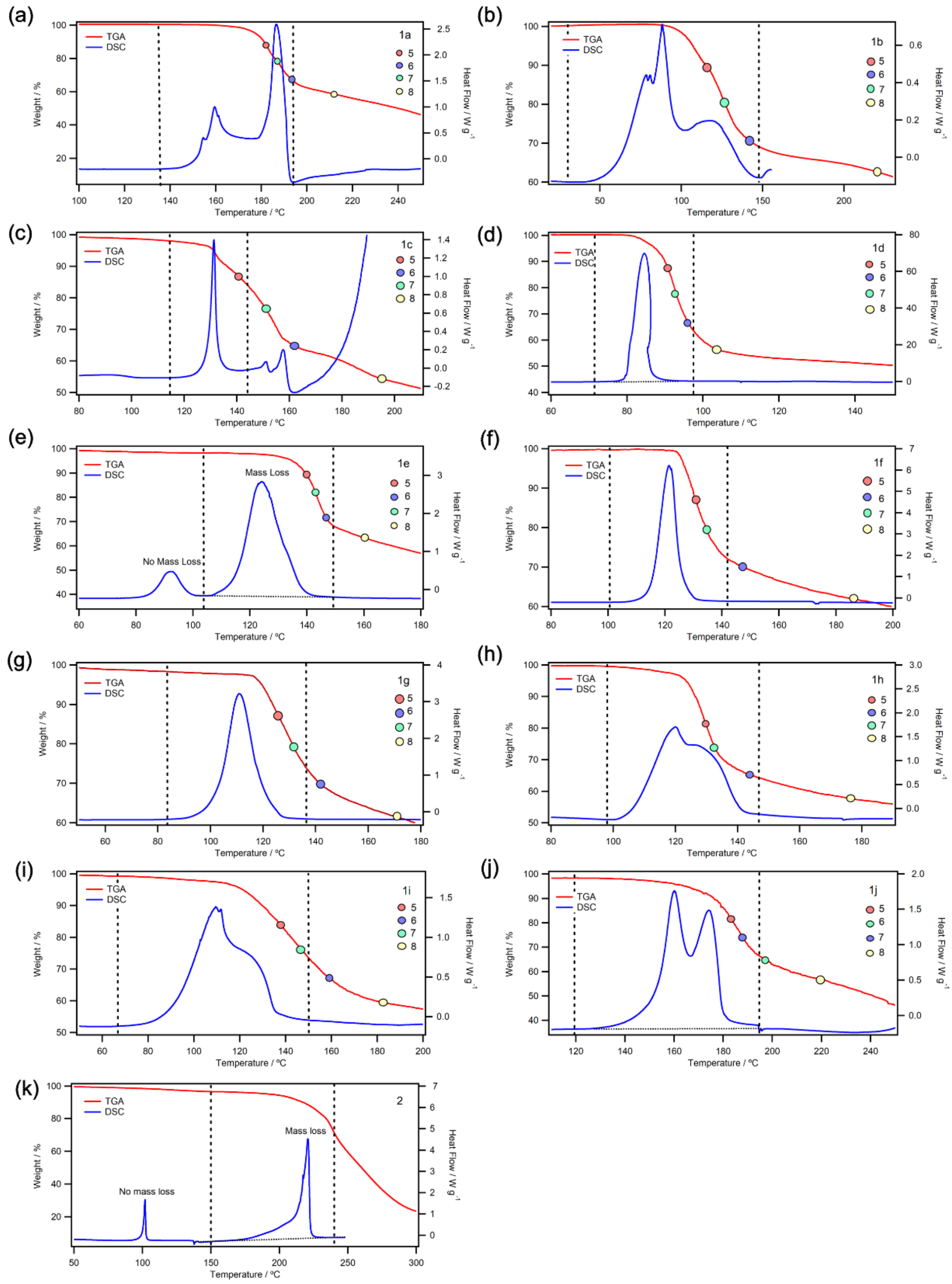

Figure S3: DSC thermograms overlaid with TGA thermograms with integration windows (dashed lines). Points 5–8 on the TGA thermogram denote calculated wt % values for the decomposition products **5**–**8** shown in Fig S13.

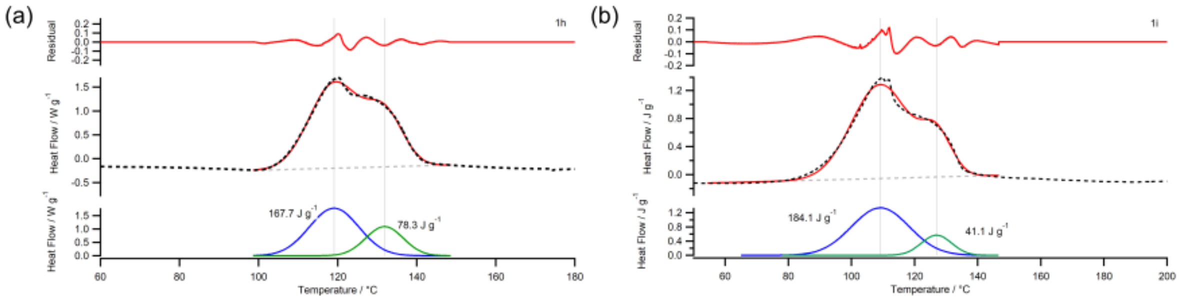

Figure S4: Gaussian peak fittings for DSC exotherms of (a) **1h** and (b) **1i**, showing peak components (bottom), fit to data (middle) and residual signal (top).

3.3 Correlation Analysis between Structural and Thermal Data

A range of structural features from XRD (Fig. S5) were analysed with respect to their effect on thermal stability, quantified by the TGA decomposition onset temperature T_{onset}.

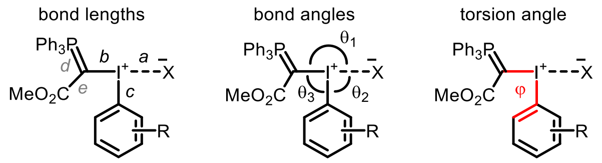

Figure S5. Definition of structural parameters investigated in this section.

The nature of the anion X has previously been shown to have a significant effect on the stability and reactivity of hypervalent iodine compounds. In this work, however, no statistically significant correlation was observed between the TGA decomposition onset temperature T_{onset} and the anions' inductive donor ability σ_m (Fig. S6)[10–12], their Kamlet-Taft hydrogen bond acceptor ability β (Fig. S7),[13] or the pK_a of the conjugate acid HX (Fig. S8). Similarly, there was no correlation between T_{onset} and the length of the I–X (*a*), I–C(ylid) (*b*) or I–C(Ar) (*c*) bonds (Fig. S9), or the bond angles θ_1, θ_2 and θ_3 around the iodine(III) centre (Fig. S10). The degree of distortion of the 3-centre-4-electron bond from 180° did not affect thermal stability either (Fig. S11). However, the torsion angle φ between the plane of the arene substituent and the hypervalent 3-centre-4-electron bond was found to have an important impact on thermal stability (Fig. S12): When the plane of the arene ring was parallel to the R–I–X bond ($\varphi < 5°$), relatively stable compounds ensued, while a large twist away from planarity resulted in compounds that were destabilised towards thermal decomposition.

Finally, the electronics of the arene substituent were considered. No direct correlation between the substituents' Hammett parameter σ_p and the TGA decomposition onset temperature T_{onset} were observed for para-substituted phosphoranyl(aryl)iodonium compounds **1f-i** (Fig. S13). However, the DSC thermograms of iodonium ylids with electron-poor aryl substituents (**1h-i**) showed two exothermic peaks that both coincided with mass loss (Fig. S3-4), which suggests two competing decomposition pathways may be occurring in these compounds. Further studies are therefore necessary to fully understand the effect of the arene substituent.

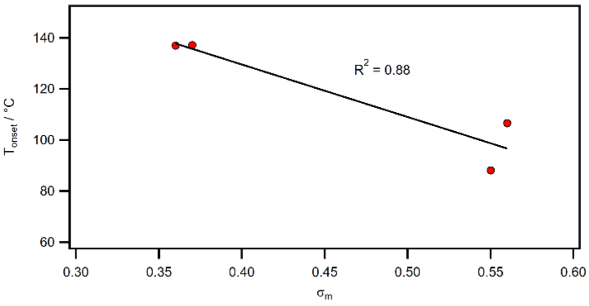

Figure S6. Correlation of anion donor ability (Hammett parameter σ_m) with TGA decomposition onset temperature T_{onset}.

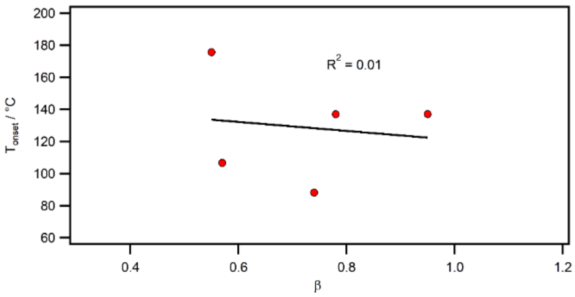

Figure S7. Correlation of anion Kamlet-Taft hydrogen bond acceptor ability β[13] with T_{onset}. (Note: Kamlet-Taft data for 1-butyl-3-methylimidazolium cations was used.)

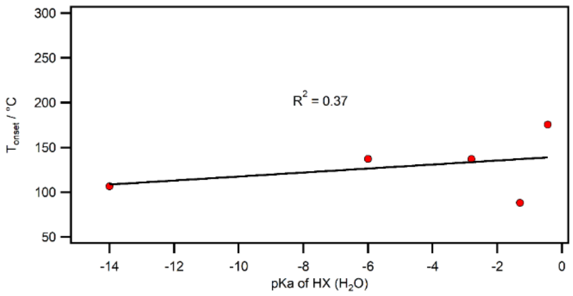

Figure S8. Correlation of anion pK_{aH} and T_{onset}.

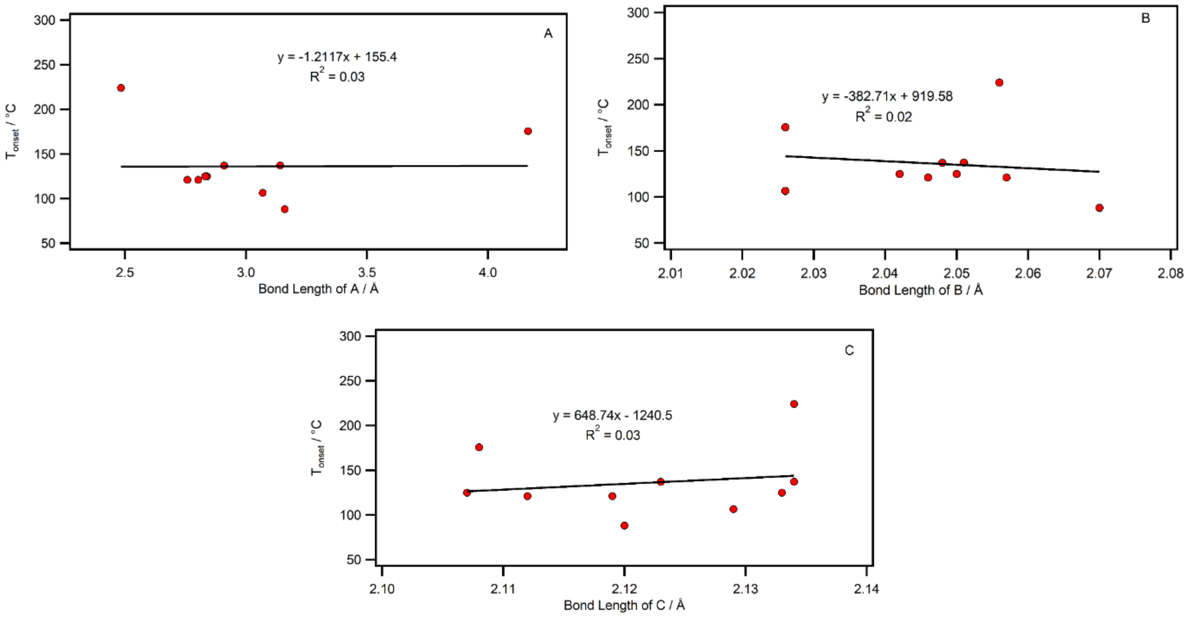

Figure S9. Correlation of bond lengths *a* (I–X), *b* (I–C_{ylid}) and *c* (I–C_{Ar}) with TGA decomposition onset temperature T_{onset}.

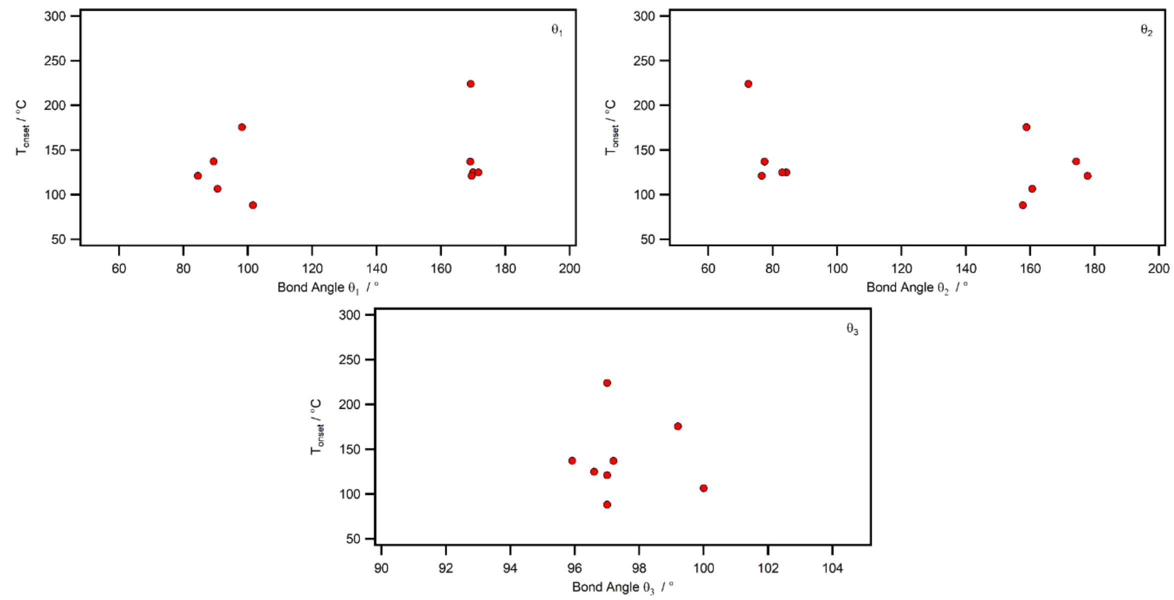

Figure S10. Correlation of bond angles θ$_1$, θ$_2$ and θ$_3$ with T_{onset}.

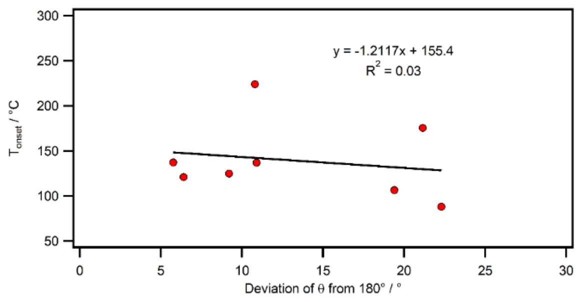

Figure S11. Correlation of the distortion of the R–I–X bond from 180° with T_{onset}.

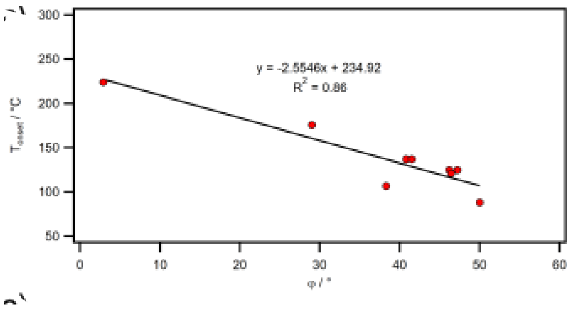

Figure S12. Correlation of torsion angles φ / 'hypervalent twist' with T_{onset}.

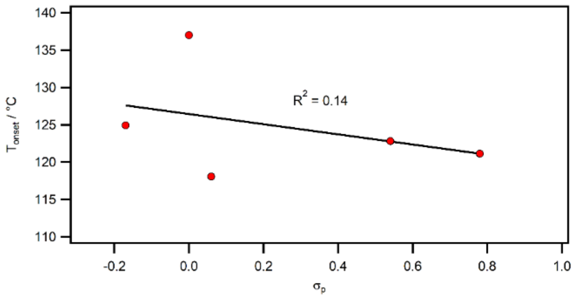

Figure S13. Correlation of *para*-Hammett substitution constant (σ_p) with T_{onset}.

3.4 Ex-Situ Analysis of Decomposition Products

Ex-situ mass spectrometry and NMR analysis was carried out on TGA samples of compounds **1a**, **1i**, **1j** and **1k** that had been held at a constant temperature T_1 under an N_2 atmosphere in open pans for 30 min or until a mass loss >5% of the original mass was observed, then heated to temperature T_2 until 20–40% mass loss of original weight. The data and a proposed decomposition mechanism are summarised in Fig. S13:

Experimental parameters	T_1	T_2
**1e**	Held at 110 °C for 13 min	Heated to 193 °C for 40% mass loss
**1i**	Held at 50 °C for 3 min	Heated to 136 °C for 20% mass loss
**1j**	Held at 90 °C for 30 min	Heated to 181 °C for 40% mass loss
**1k**	Held at 140 °C for 22 min	Heated to 205 °C for 40% mass loss

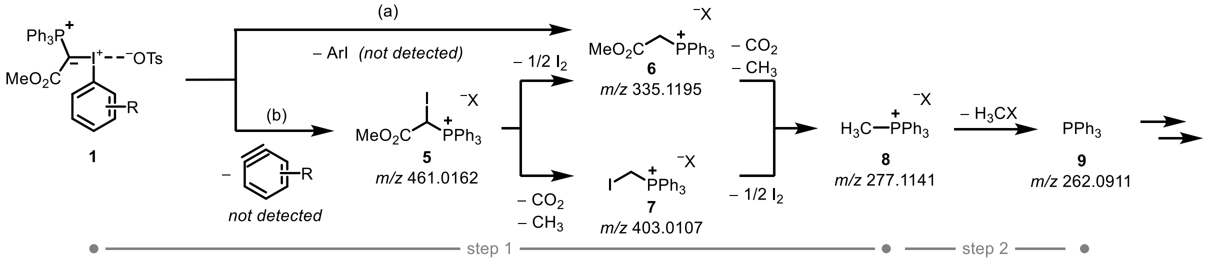

Figure S14. TGA ex-situ analysis: experimental parameters and summary of data.

(Methoxycarbonyliodomethyl)triphenylphosphonium tosylate (5)

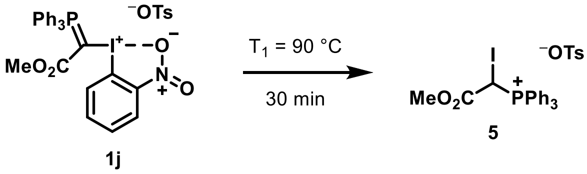

5 was detected by HRMS after compound **1j** was heated to 90 °C for 30 min.
HRMS (ESI$^+$) *m/z* calcd. for $C_{21}H_{19}IO_2P$ [M–OTs]$^+$ 461.0162, detected 461.0116.

(Methyloxycarbonylmethyl)triphenylphosphonium tosylate (6)

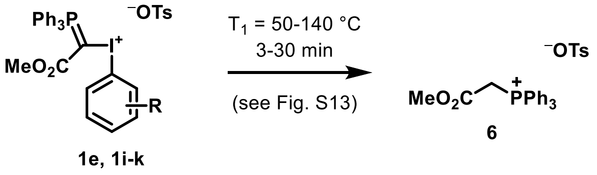

6 was detected by HRMS, ^{1}H NMR and ^{31}P NMR spectroscopy after compounds **1e**, **1i**, **1j** or **1k** were heated to T_1 (as specified in Fig. S13) for the time specified in Fig. S13 (T_1). Analytical data matched that recorded in the literature.[14]

^{1}H NMR (400 MHz, CD_3OD) δ_H 7.86–7.60 (m, 15H) 7.48–7.33 (m, 2H), 7.26–7.17 (m, 2H), 4.99 (d, J = 14.5 Hz, 2H), 3.47 (s, 3H), 2.22 (s, 3H); **^{31}P NMR** (162 MHz, $CDCl_3$) δ_P 19.4 (s, 1P); **HRMS** (ESI$^+$) *m/z* calcd. for $C_{21}H_{19}IO_2P$ [M–OTs]$^+$ 335.1195, detected 335.1199.

(Iodomethyl)triphenylphosphonium tosylate (7)

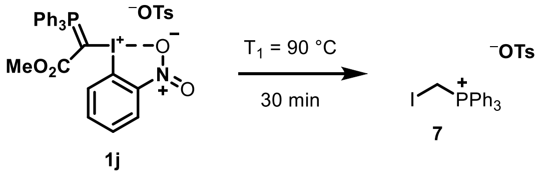

7 was detected by HRMS, ^{1}H NMR and ^{31}P NMR after compound **1k** was heated to 90 °C for 30 min. Analytical data matched that recorded in the literature.[15,16]

^{1}H NMR (400 MHz, CD3OD) δ_H 7.83–7.59 (m, 17H), 7.23 (d, *J* = 8.0 Hz, 2H), 3.63 (d, J = 12.5 Hz, 2H), 3.61 (s, 3H), 2.37 (s, 3H); **^{31}P NMR** (162 MHz, $CDCl_3$) δ_P 29.6; **HRMS** (ESI$^+$) *m/z* calcd. for $C_{19}H_{17}IP$ [M–OTs]$^+$ 403.0107, detected 403.0105.

(Methyl)triphenylphosphonium tosylate 8

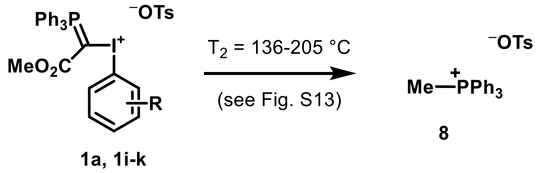

8 was detected by HRMS, ^{1}H NMR and ^{31}P NMR after compounds **1a**, **1i**, **1j** and **1k** were heated to T_2 (as specified in Fig. S13). Analytical data matched that recorded in the literature.[17]

^{1}H NMR (400 MHz, CD3OD) δ_H 7.94 – 7.51 (m, 17H), 7.23 (d, *J* = 8.0 Hz, 2H), 2.98 (d, *J* = 14.0 Hz, 3H), 2.36 (s, 3H); **^{31}P NMR** (162 MHz, $CDCl_3$) δ_P 33.4 (s, 1P); **HRMS** (ESI$^+$) *m/z* calcd. for $C_{19}H_{18}P$ [M–OTs]$^+$ 277.1141, detected 277.1140.

Triphenylphosphine 9

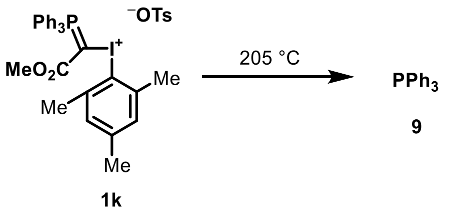

9 was detected by HRMS after compound **1k** was heated to 205 °C.

HRMS (ESI$^+$) *m/z* calcd. for $C_{18}H_{16}P$ [M+H]$^+$ 263.0984, detected 263.0977.

4. XRD Data

4.1 General Method

Single crystals were selected and mounted using Fomblin® (YR-1800 perfluoropolyether oil) on a polymer-tipped MiTeGen MicroMountTM and cooled rapidly to 120 K in a stream of cold N_2 using an Oxford Cryosystems open flow cryostat.[18] Single crystal X-ray diffraction data for **1a** were collected on a Rigaku XtaLAB PRO MM007 (PILATUS3 R 200K Hybrid Pixel Array detector, mirror-monochromated Cu-Kα radiation source; λ = 1.54184 Å, ω scans). Single crystal X-ray diffraction data for **1b**, **1c** and **1f-hydrate2** were collected on an Oxford Diffraction SuperNova GV1000 (AtlasS2 CCD area detector, mirror-monochromated Cu-Kα radiation source; λ = 1.54184 Å, ω scans). Single crystal X-ray diffraction data for **1d**, **1e**, **1f-hydrate1** and **1i** were collected on an Oxford Diffraction SuperNova GV1000 (AtlasS2 CCD area detector, mirror-monochromated Cu-Kα radiation source; λ = 1.54184 Å, ω scans). Cell parameters were refined from the observed positions of all strong reflections and absorption corrections were applied using a Gaussian numerical method with beam profile correction (CrysAlisPro).[19] Structures were solved within Olex2[20] by dual space iterative methods (SHELXT)[21] and all non-hydrogen atoms refined by full-matrix least-squares on all unique F2 values with anisotropic displacement parameters (SHELXL).[22] Structures were checked with checkCIF (http://checkcif.iucr.org). CCDC- 2367436-2367443 contains the supplementary data for these compounds. These data can be obtained free of charge from The Cambridge Crystallographic Data Centre via www.ccdc.cam.ac.uk/data_request/cif.

4.2 Single Crystal X-Ray Diffraction Experimental Parameters: Summary

	**1a**	**1b**	**1c**	**1d**	**1e**	**1f-hydrate1**	**1f-hydrate2**	**1i**
Chemical formula moiety	$C_{27}H_{23}IO_2P·BF_4$	$C_{27}H_{23}IO_2P·$ $CF_3SO_3·C_4H_{10}O$	$C_{27}H_{23}IO_2P·Cl·$ CH_4O	$C_{27}H_{23}IO_2P·NO_3·H_2O$	$C_{27}H_{23}IO_2P·$ $C_7H_7SO_3$	$C_{28}H_{25}IO_2P·$ $C_7H_7SO_3·$ $1.25(H_2O)$	$C_{28}H_{25}IO_2P·$ $C_7H_7SO_3·$ $1.35(H_2O)$	$C_{27}H_{22}INO_4P·$ $C_7H_7SO_3·$ $0.75(C_4H_{10}O)$
Formula sum	$C_{27}H_{23}BF_4IO_2P$	$C_{32}H_{33}O_6F_3PSI$	$C_{28}H_{27}ClIO_3P$	$C_{27}H_{25}INO_6P$	$C_{34}H_{30}IO_5PS$	$C_{35}H_{34.5}IO_{6.2}PS$	$C_{35}H_{32}IO_{6.3}PS$	$C_{37}H_{36.5}INO_{7.8}PS$
M_r	624.13	760.51	604.81	617.35	708.51	745.05	744.05	809.10
Crystal system, space group	Monoclinic, $P2_1/n$	Monoclinic, $I2/a$	Triclinic, $P\bar{1}$	Triclinic, $P\bar{1}$	Tetragonal, $P4_32_12$	Triclinic, $P\bar{1}$	Triclinic, $P\bar{1}$	Triclinic, $P\bar{1}$
a, *b*, *c* (Å)	12.4385 (4), 13.1266 (4), 16.0230 (6)	20.2187 (9), 13.3358 (5), 25.8451 (12)	9.6183 (2), 10.3151 (3), 15.1187 (3)	9.6938 (8), 9.8329 (10), 15.9817 (11)	9.9300 (1), 9.9300 (1), 61.4074 (8)	13.1499 (5), 13.5605 (4), 19.5611 (4)	13.5758 (5), 16.2685 (4), 16.5108 (3)	15.5011 (2), 16.8577 (3), 17.4634 (3)
α, β, γ (°)	90, 91.027 (3), 90	90, 111.822 (5), 90	90.597 (2), 102.945 (2), 116.300 (3)	73.248 (8), 87.642 (6), 61.128 (9)	90, 90, 90	106.193 (2), 90.770 (2), 97.815 (3)	106.331 (2), 94.206 (2), 105.684 (3)	113.219 (2), 101.897 (1), 105.529 (1)
V (Å^3)	2615.74 (15)	6469.3 (5)	1300.17 (6)	1268.6 (2)	6055.07 (15)	3313.79 (18)	3324.81 (17)	3782.05 (12)
Z	4	8	2	2	8	4	4	4
Z'	1	1	1	1	1	2	2	2
μ (mm^{-1})	10.65	9.37	11.42	10.88	9.80	9.01	8.98	7.98
Crystal size (mm)	0.19 × 0.06 × 0.03	0.12 × 0.06 × 0.03	0.16 × 0.13 × 0.11	0.15 × 0.11 × 0.05	0.13 × 0.05 × 0.03	0.11 × 0.09 × 0.04	0.11 × 0.10 × 0.05	0.24 × 0.13 × 0.05
Diffractometer	XtalLAB PRO *MM007*, PILATUS3 R 200K	SuperNova, Atlas S2	SuperNova, Atlas S2	SuperNova, Titan S2	SuperNova, Titan S2	SuperNova, TitanS2	SuperNova, Atlas S2	SuperNova, Titan S2
T_{min}, T_{max}	0.337, 0.859	0.482, 0.830	0.298, 0.550	0.316, 0.726	0.391, 0.875	0.565, 0.836	0.496, 0.820	0.248, 1.000
No. of measured, independent and observed [$I > 2\sigma(I)$] reflections	25908, 5277, 4697	23115, 6330, 5657	19215, 5069, 5047	15472, 4816, 4030	90819, 5809, 5687	26653, 12758, 9796	53875, 13080, 11237	65660, 14787, 13687
R_{int}	0.070	0.059	0.045	0.107	0.062	0.054	0.067	0.061
θ_{max} (°)	76.3	73.5	72.7	76.1	70.9	72.8	73.3	72.5
$(\sin \theta/\lambda)_{max}$ (Å^{-1})	0.630	0.622	0.619	0.630	0.613	0.619	0.621	0.618
$R[F^2 > 2\sigma(F^2)]$, $wR(F^2)$, S	0.069, 0.199, 1.05	0.051, 0.141, 1.08	0.020, 0.050, 1.04	0.104, 0.302, 1.05	0.030, 0.074, 1.09	0.056, 0.160, 1.03	0.056, 0.156, 1.05	0.053, 0.148, 1.07
No. of reflections	5277	6330	5069	4816	5809	12758	13080	14787
No. of parameters	359	400	312	329	436	824	836	909
No. of restraints	812	0	1	0	54	3	154	219
$_{max}$, $\Delta\rangle_{min}$ (e Å^{-3})	1.01, -2.22	1.23, -2.14	0.77, -0.59	4.26, -3.37	0.72, -0.98	1.72, -2.10	1.27, -2.05	2.29, -1.38
Absolute Structure (Flack)	-	-	-	-	-0.016 (2)	-	-	-
CCDC	2367436	2367437	2367438	2367439	2367440	2367441	2367442	2367443

4.3 Refinement Details

Phenyliodonium(methyl (triphenylphosphoranylidene)acetate) tetrafluoroborate (1a)

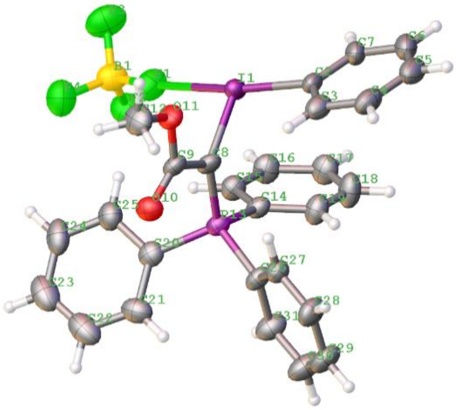

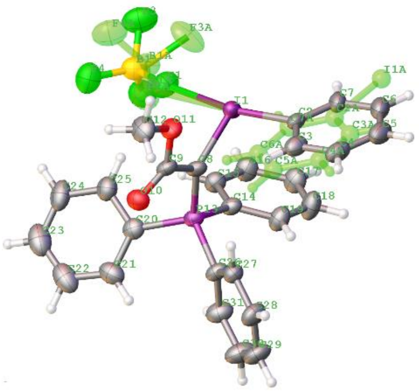

Before a disordered iodophenyl ring residue was included in the model a large residual electron density peak of height 8.4 e Å^{-3} was observed 1.76 and 1.64 Å from phenyl carbon atoms C6 and C7 respectively. The PLATON tools AddSymm and TwinRotMAt did not indicate the presence of missed symmetry or non-merohedral twinning. The large residual electron density peak could not be accounted for with a disorder component that made chemical sense with respect to the main residue of the structure. When the large residual electron density peak was modelled as an iodine atom with a free occupancy it refined to a value of 0.07 and further adjacent residual electron density peaks resembling a phenyl ring overlapping phenyl ring C2-C7 became apparent in the electron density map. The location of this iodophenyl fragment does not make chemical sense with respect to the main residue and is unlikely to be a disorder component, rather and artefact of undiagnosed twinning or whole molecule disorder.

Inclusion of the alternative iodophenyl residue in the model reduced the value of R1 from 9.50% to 7.07% and made the displacement ellipsoids of the main residue more chemically congruous, hence, it was decided to keep the chemically unexplained disorder component in the model. The occupancies of the main residue iodophenyl atoms and minor component are refined and constrained to sum to unity, giving the minor component an occupancy of 0.07(1). The carbon atoms of the phenyl ring of the minor component are constrained to have regular hexagonal geometry (AFIX 66) and make a symmetrical bond to the minor occupancy iodine atom (SADI, FLAT). The minor occupancy atoms were modelled with isotropic displacement parameters, with the iodine atom fixed at a value of 0.032 (to match the Ueq of the major iodine atom) and the phenyl carbon atoms all constrained to refine to a common value of 0.032. Rigid bond and similarity restraints were applied to all isotropic and anisotropic displacement parameters in the structure (RIGU, SIMU).

Conformational disorder is modelled for the tetrafluoroborate anion with the occupancies of the two components refined and constrained to sum to unity, giving values of 0.73(1) and 0.27(1) respectively. The geometries of the two anions were restrained to be similar (SADI) and the anisotropic displacement parameters of the closely overlapping fluorine and boron atoms constrained to be identical (EADP).

Phenyliodonium(methyl (triphenylphosphoranylidene)acetate) triflate (1b)

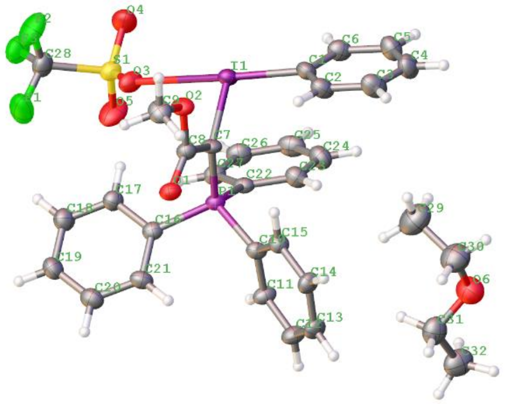

Residual electron density peak close to I1 (1.23 e $Å^{-3}$, 1.44 Å from I1) could not be modelled as disorder components. The residual peaks are potentially caused by deficiencies in the absorption correction or undiagnosed twinning.

Phenyliodonium(methyl (triphenylphosphoranylidene)acetate) chloride (1c)

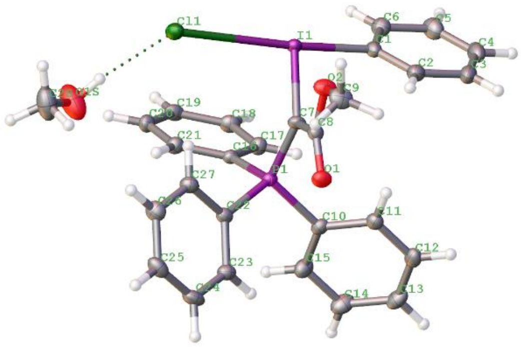

All H atoms were observed in the electron density map. The position of hydroxy hydrogen atom H1S was refined with the O-H distance restrained to a target value of 0.84 Å (DFIX, ESD 0.02 Å). All of the hydrogen atoms were geometrically placed and refined with a riding model.'

Phenyliodonium(methyl (triphenylphosphoranylidene)acetate) nitrate (1d)

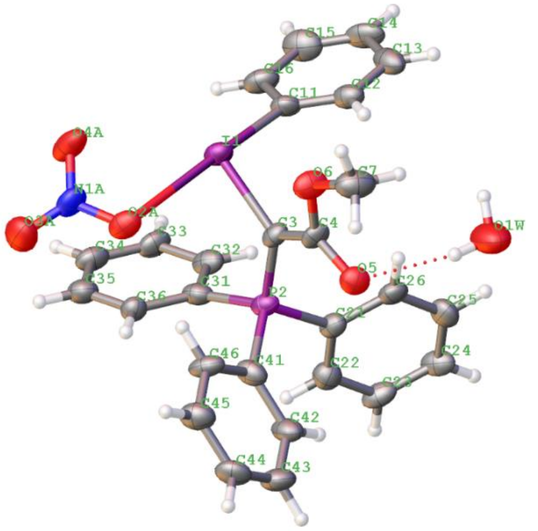

Large residual electron density peaks with heights 4.26 and 3.69 e Å^{-3} are located on opposite sides of iodine atom I1 at distances of 1.03 and 0.96 Å respectively. These peaks are likely caused by deficiencies in the efficacy of the Gaussian face-based absorption correction for the heavy atom structure that was lamentably collected using Cu radiation. The influence of the heavy atoms on the Fourier map may have perturbed the coordinates of nearby lighter atoms and hence decreased the precision of derived C-C distances.

The location of the water hydrogen atoms for residue O1W were not apparent in the electron density map, despite the presence of two appropriately located hydrogen bond acceptor atoms. The water residue is refined as rigid body (AFIX 6) with O-H distance fixed at 0.87 Å and an H...H distance of 1.38 Å. All other hydrogen atoms were observed in the electron density map before being geometrically placed and refined with a riding model.

Phenyliodonium(methyl (triphenylphosphoranylidene)acetate) tosylate (1e)

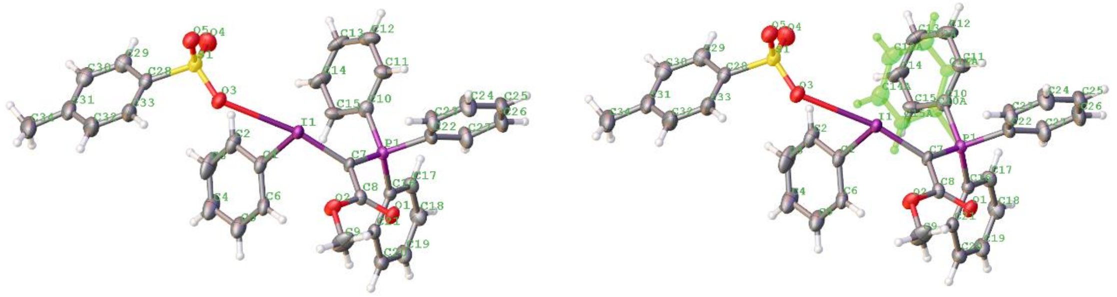

The chemically identical C-C bond lengths of the phenyl moiety comprising of carbon atoms C10, C11, C12, C13, C14, C15 were restrained to have similar distances (SADI) and the geometry of the rings was restrained to be planar (FLAT).

Positional disorder is modelled for one of the phenyl substituents of P1; their pairs of occupancies were refined and respectively constrained to sum to unity resulting in values of 0.50(2) for each.

All hydrogen atoms were observed in the electron density map before being geometrically placed and refined with a riding model.

4-Methylphenyliodonium(methyl (triphenylphosphoranylidene)acetate) tosylate (1f-hydrate1)

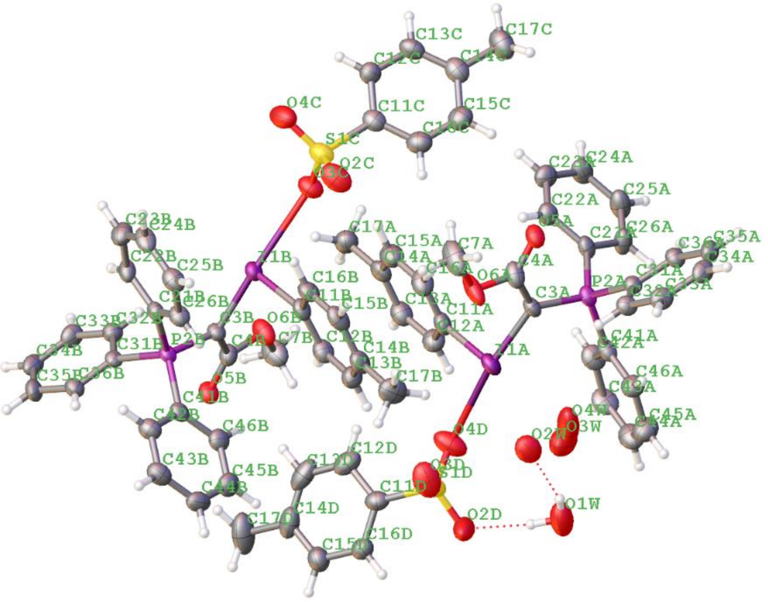

'The hydrogen atoms of water residue O1W were refined with the O-H distances and H...H distance restrained to target values of 0.87 and 1.42 Å (DFIX, esd 0.02 and 0.04 Å respectively). Water residues O2W and O3W are in close proximity to each other and are modelled as mutually exclusive disorder components with their occupancies refined and constrained to sum to unity giving values of 0.34(2) and 0.66(2) respectively. Water residue O4W was modelled at a fixed occupancy of 0.5. The positions of the hydrogen atoms of water residues O2W, O3W and O4W could not be determined from the electron density map despite the presence of suitable proximate hydrogen bond acceptors. The hydrogen atoms for these residues were not included in the model but were included in the unit cell contents and all derived parameters. All other hydrogen atoms were observed in the electron density map before being geometrically placed and refined with a riding model.

4-Methylphenyliodonium(methyl (triphenylphosphoranylidene)acetate) tosylate (1f-hydrate2)

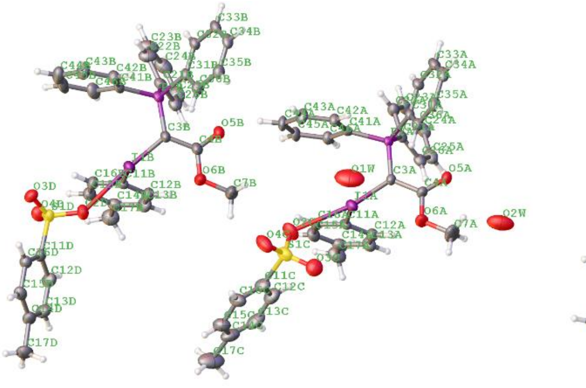

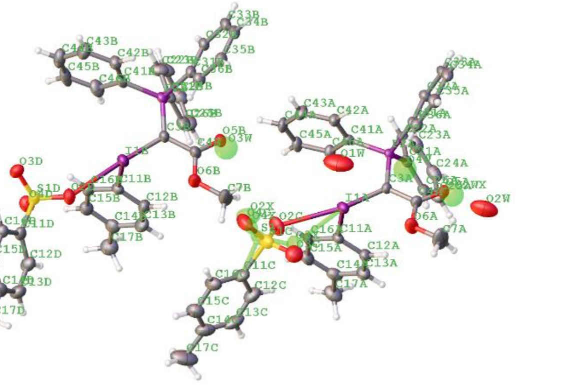

Disorder is modelled for the sulphonate moiety of tosylate anion C with the SO3 atoms found in two conformations the occupancies of which were refined and constrained to sum to unit giving values of 0.61(1) and 0.39(1). The geometries of the two sulphonate disorder components were restrained to be similar (SADI) and rigid bond and similarity restraints were applied to their anisotropic displacement parameters (RIGU, SIMU). The anisotropic displacement parameters of closely overlapping oxygen atoms O4C/X and sulphur atoms S1C/X were constrained to be identical (EADP).

Water residue O2W/X is modelled as disordered over two sites the occupancies of which were refined and constrained to sum to unit giving values of 0.81(1) and 0.19(1). Minor occupancy component O2X is refined with an isotropic displacement parameter fixed at a value of 0.1 matching the Ueq of the major component. The occupancies of water residues O3W and O4W were refined to values of 0.37(1) and 0.32(1) respectively and precluded from bonding to their proximate symmetry equivalent sites. Water residues O3W and O4W were refined with isotropic displacement parameters fixed at values of 0.1 to match the Ueq value of water residue O1W. The hydrogen atoms of all the water residues were not apparent in the electron density map despite the presence of many plausible hydrogen bond acceptors and donors, and were not included in the model, however they were included in the unit cell contents and all derived parameters. All other hydrogen atoms were observed in the electron density map before being geometrically placed and refined with a riding model.

4-Nitrophenyliodonium(methyl (triphenylphosphoranylidene)acetate) tosylate (1i)

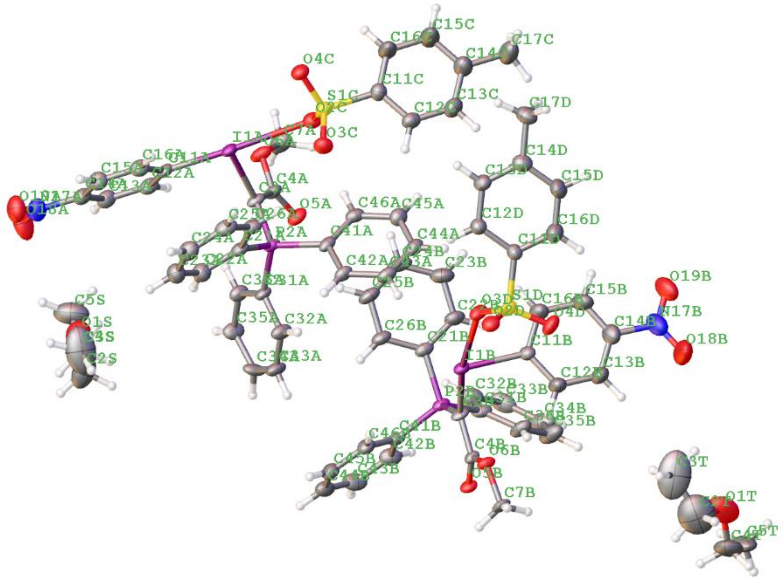

Diethyl ether solvent residue T is modelled with a fixed occupancy of 0.5. A sensible model for a second overlapping disorder model from the residual electron density around this residue could not be developed. The geometries of the two diethyl ether residues were restrained to be similar and symmetrical (SAME). Rigid bond and similarity were applied to the anisotropic displacement parameters of all atoms in both diethyl ether residues (RIGU, SIMU). The anisotropic displacement parameters of all atoms in residue T and ethyl carbon atoms C2S and C3S of residue S were restrained to have more isotropic character (ISOR).

A residual electron density peak with height 2.29 e Å^{-3} is observed 1.47 Å from triphenylphosphine carbon atom C24B. This peak cannot be accounted for as part of a chemically sensible disorder model. No missing symmetry or twin laws were detected by the Platon tools AddSymm and TwinRotMat. This residual electron density peak can likely be attributed to undiagnosed twinning.

5. Copies of NMR Spectra

Phenyliodonium(methyl (triphenylphosphoranylidene)acetate) tetrafluoroborate (1a)

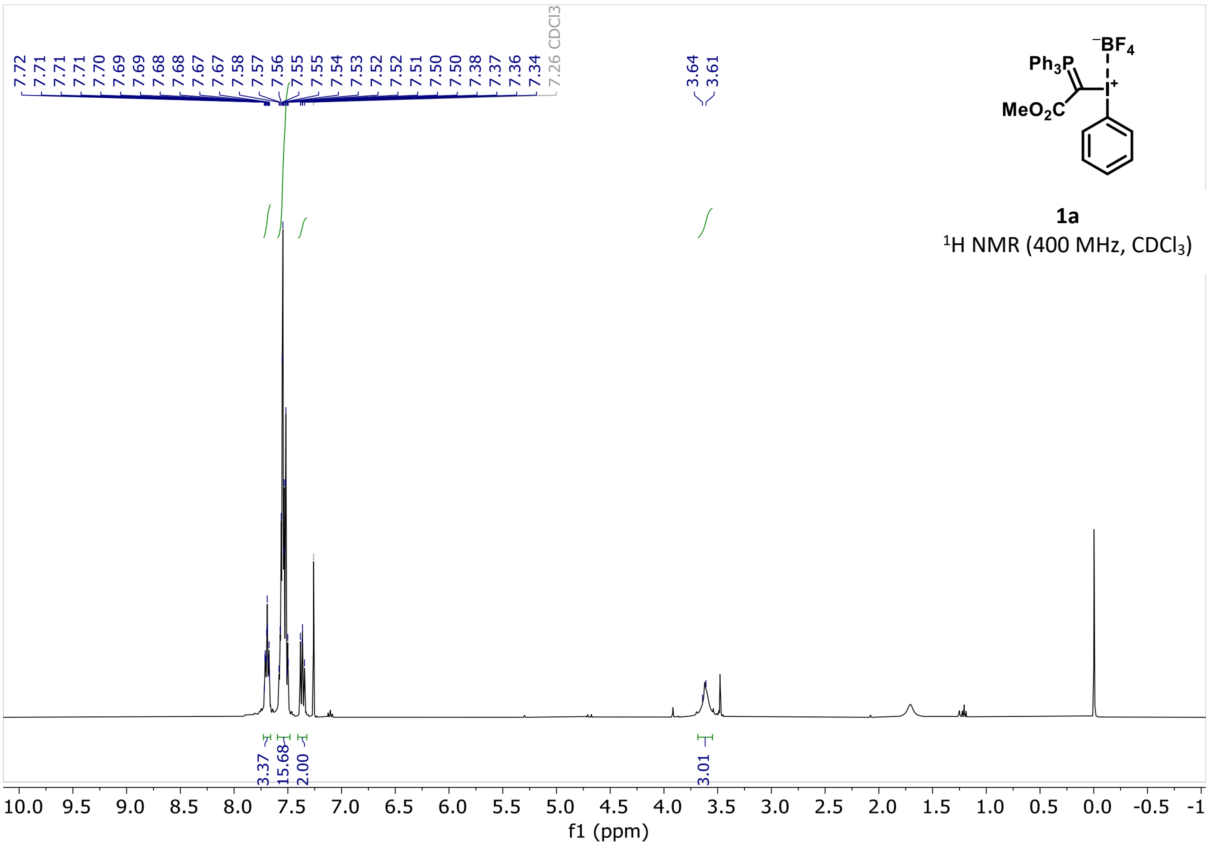

1a

^{1}H NMR (400 MHz, $CDCl_3$)

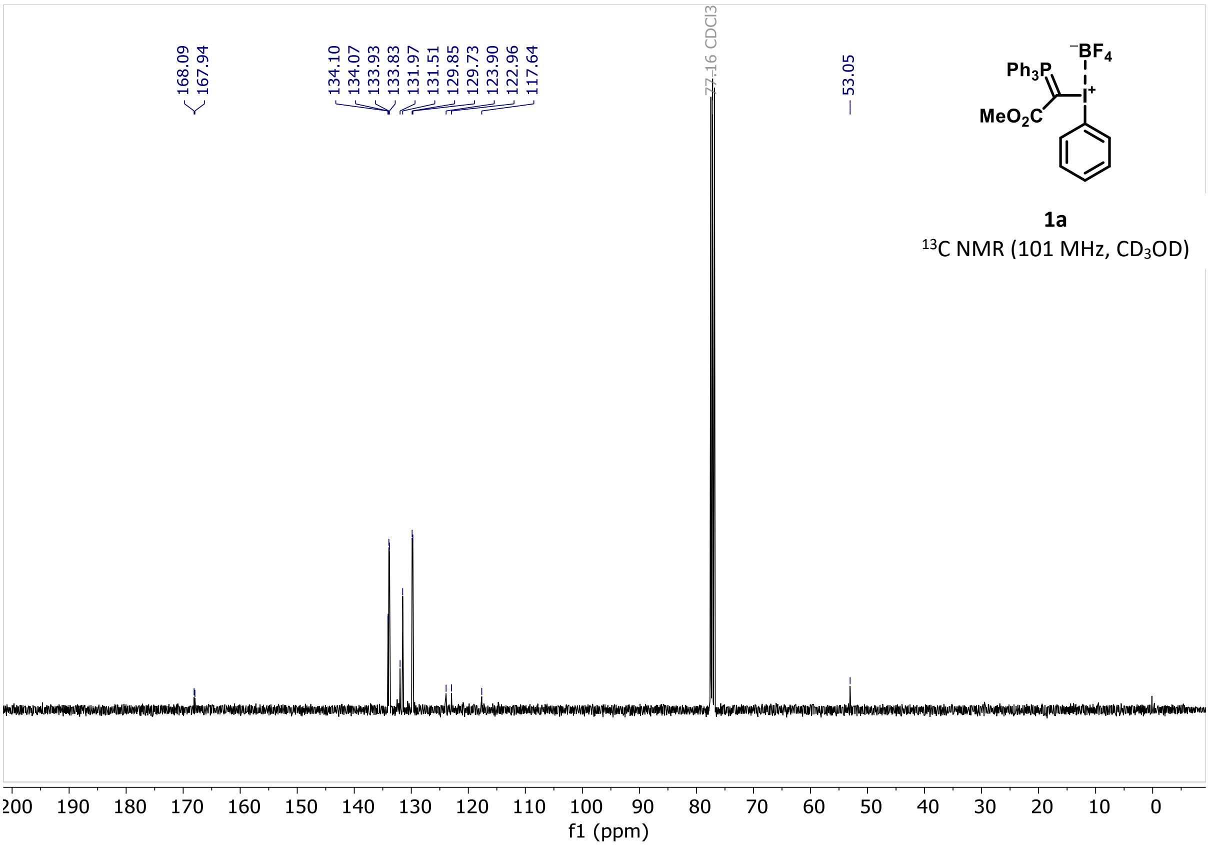

1a

^{13}C NMR (101 MHz, CD_3OD)

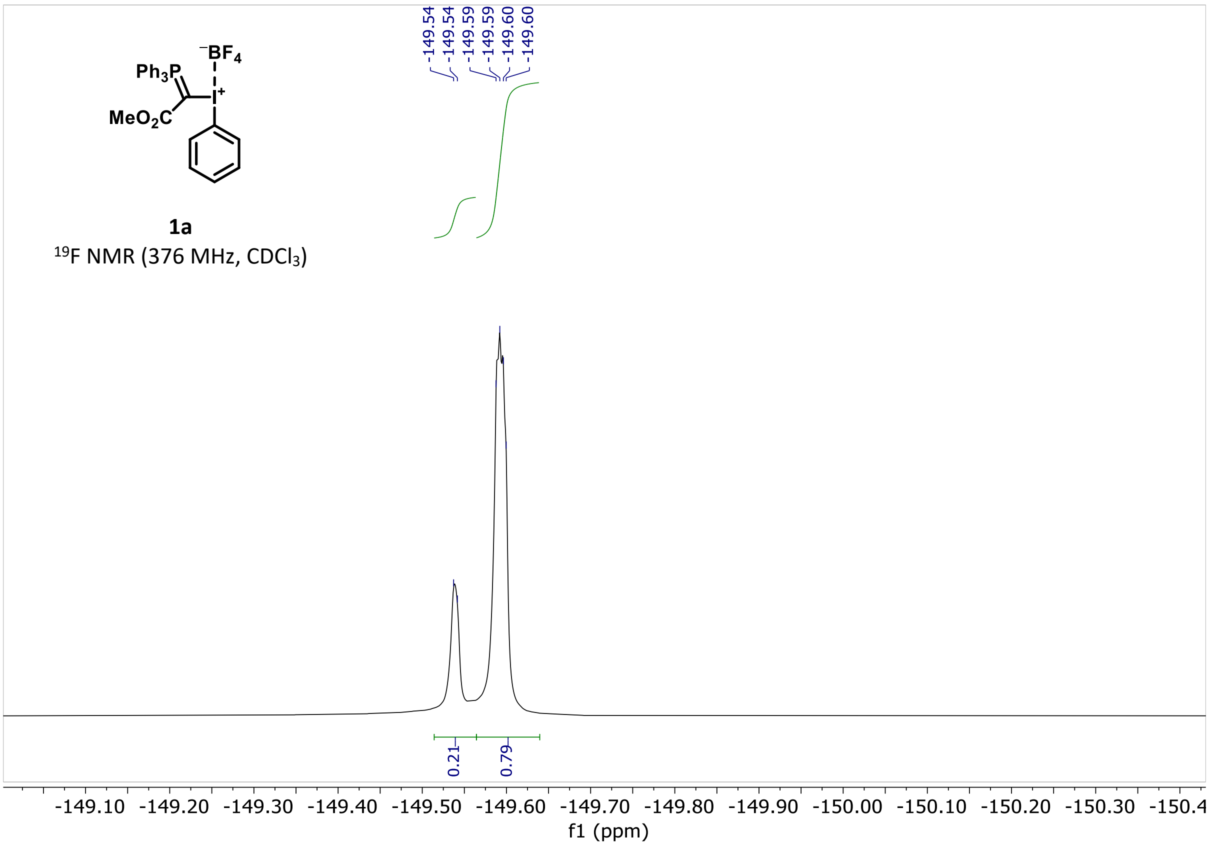

−BF4
Ph3P
I+
MeO2C
1a
19F NMR (376 MHz, CDCl3)
-149.54
-149.54
-149.59
-149.59
-149.60
-149.60
0.21
0.79
-149.10 -149.20 -149.30 -149.40 -149.50 -149.60 -149.70 -149.80 -149.90 -150.00 -150.10 -150.20 -150.30 -150.4
f1 (ppm)

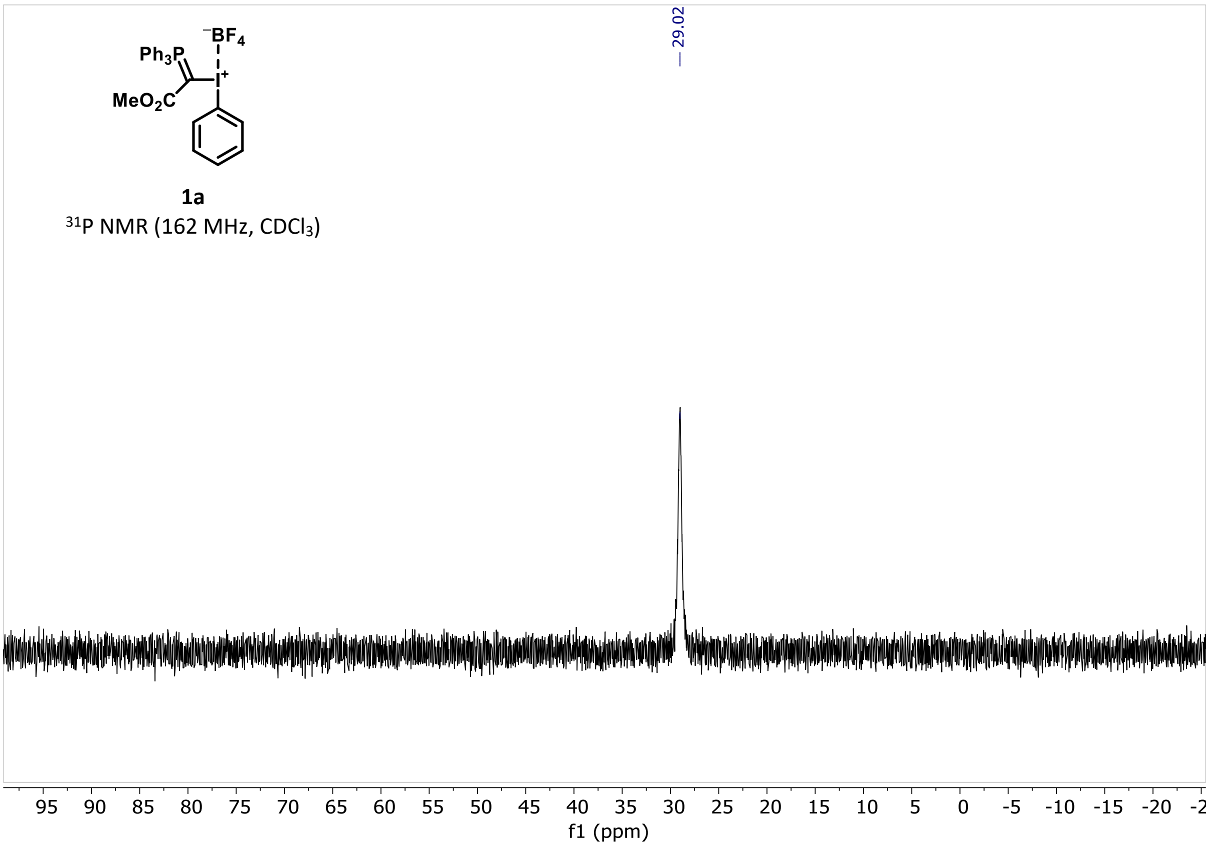

−BF4
Ph3P
I+
MeO2C
1a
31P NMR (162 MHz, CDCl3)
29.02
95 90 85 80 75 70 65 60 55 50 45 40 35 30 25 20 15 10 5 0 -5 -10 -15 -20 -2
f1 (ppm)

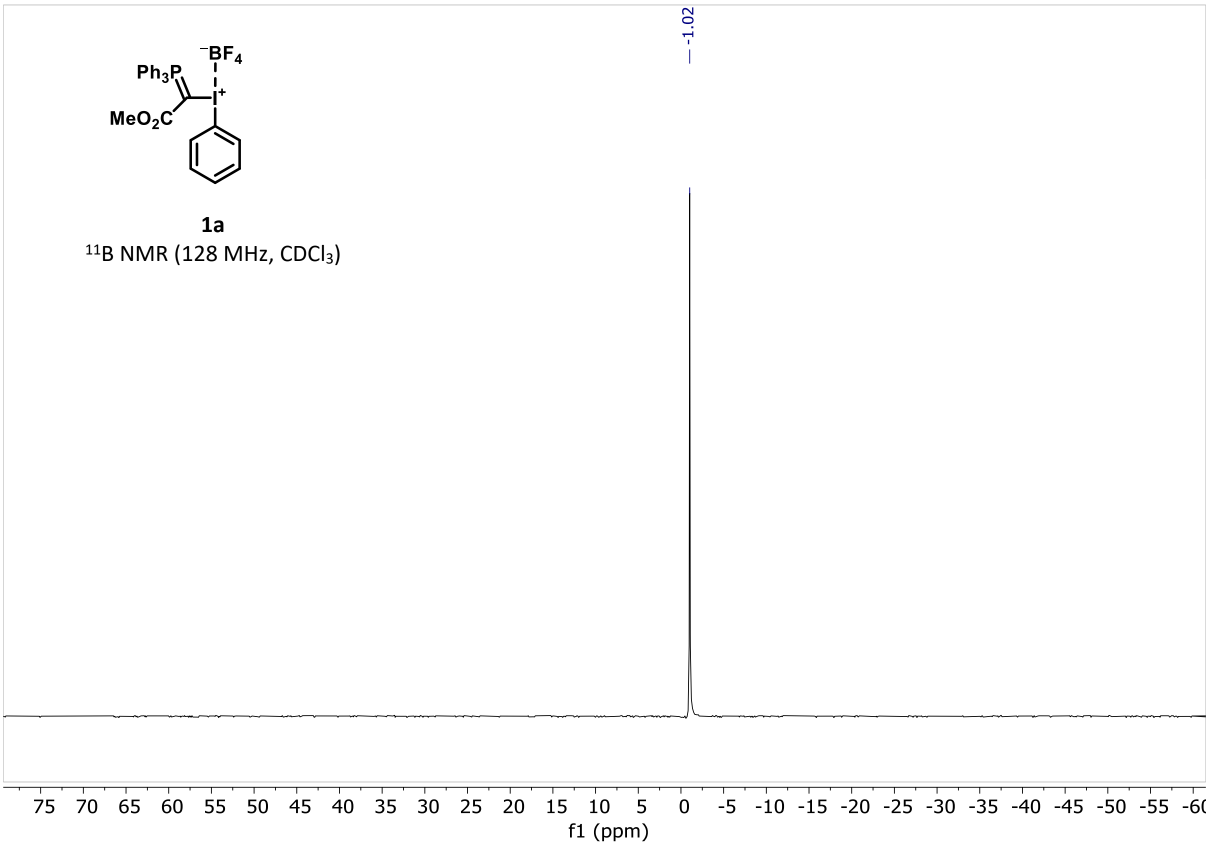

Phenyliodonium(methyl (triphenylphosphoranylidene)acetate) triflate (1b)

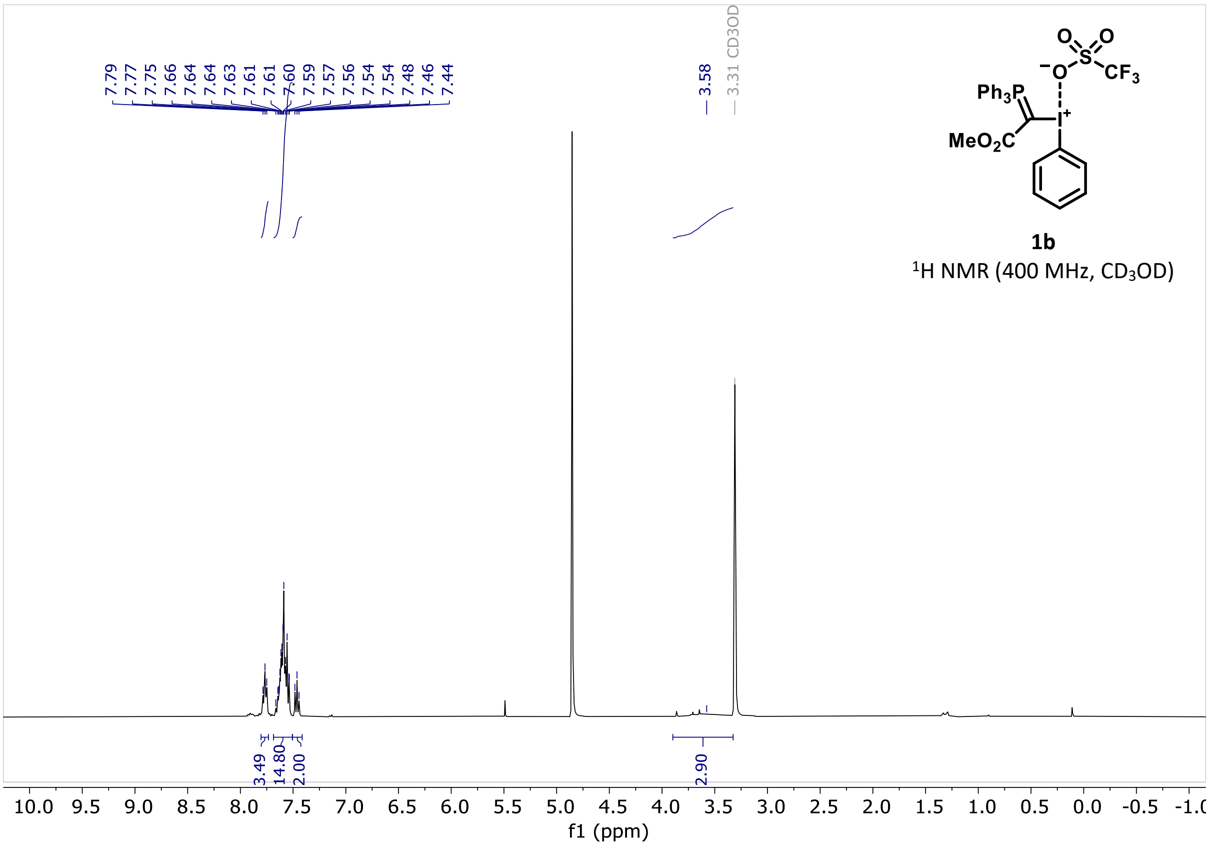

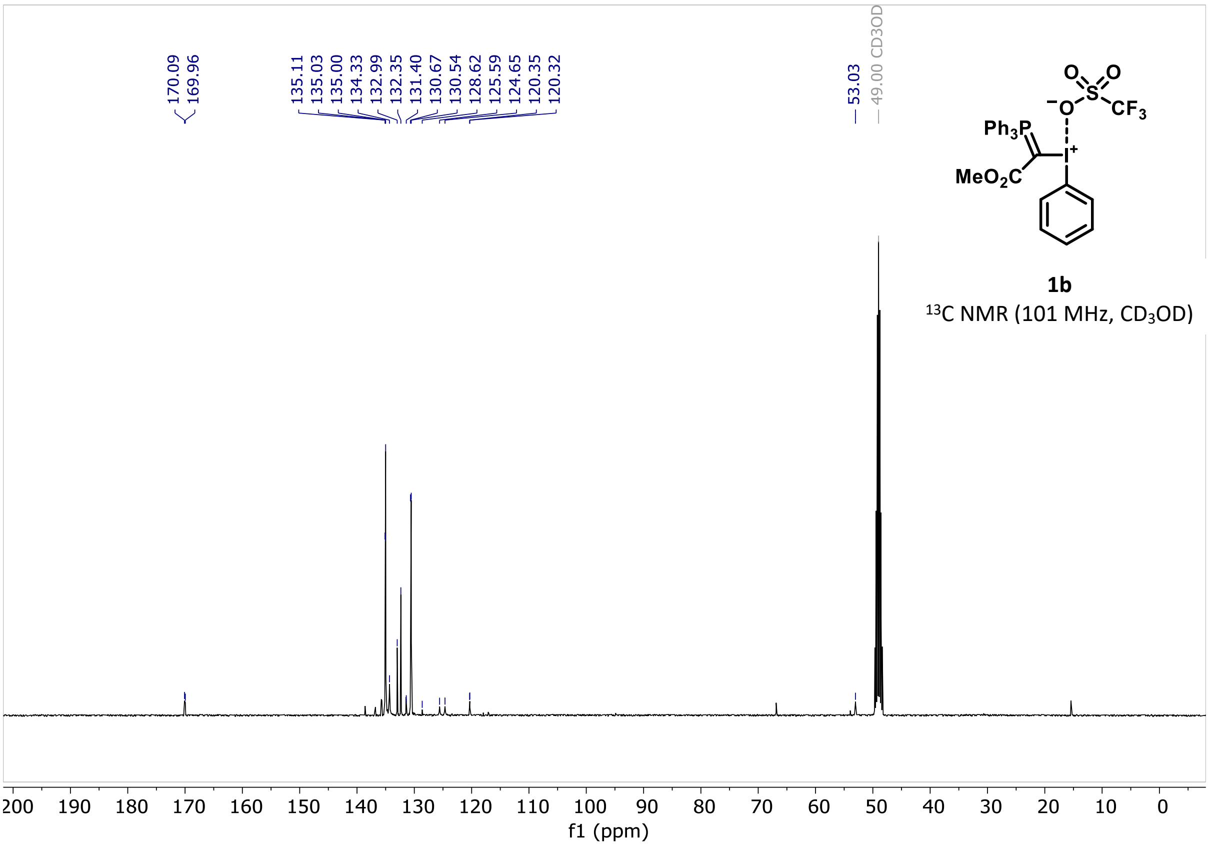
170.09
169.96
135.11
135.03
135.00
134.33
132.99
132.35
131.40
130.67
130.54
128.62
125.59
124.65
120.35
120.32
53.03
49.00 CD3OD
Ph3P
MeO2C
CF3
1b
13C NMR (101 MHz, CD3OD)
200 190 180 170 160 150 140 130 120 110 100 90 80 70 60 50 40 30 20 10 0
f1 (ppm)

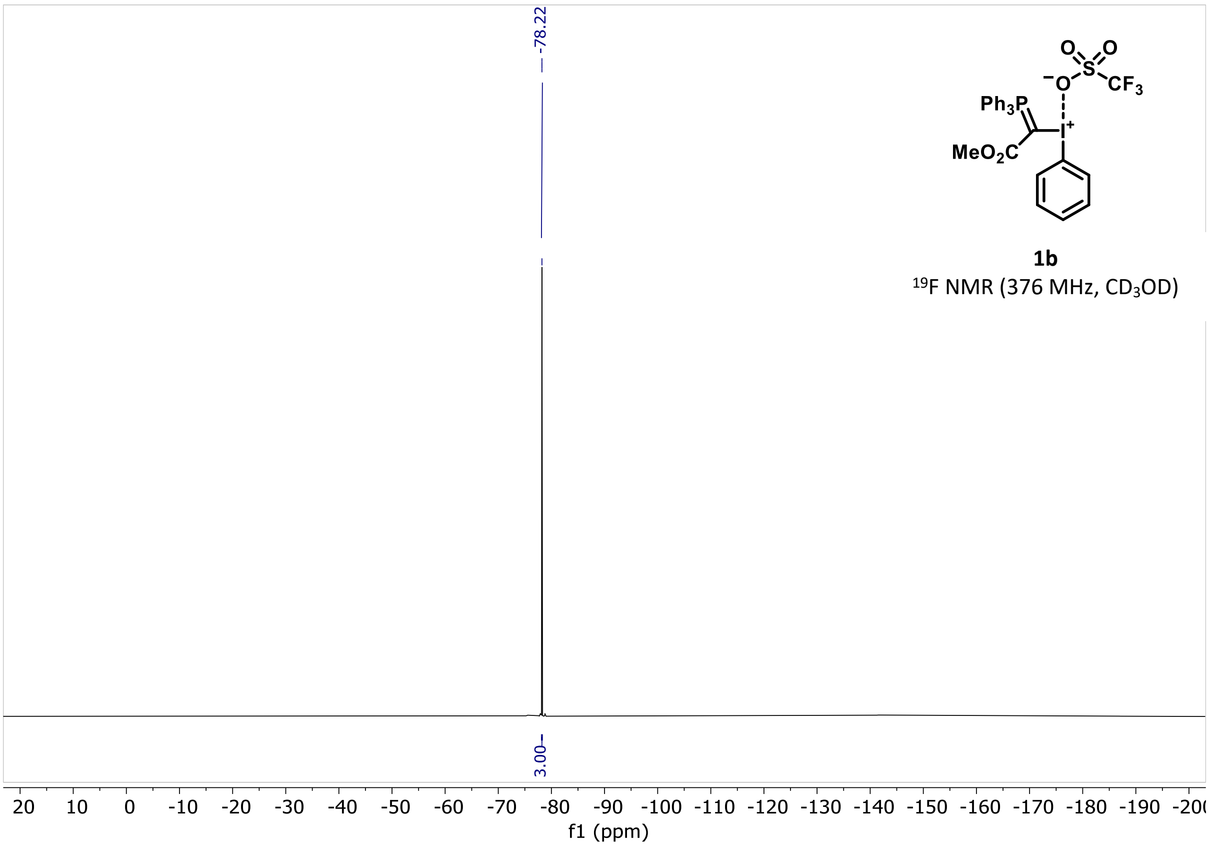
-78.22
3.00
Ph3P
MeO2C
CF3
1b
19F NMR (376 MHz, CD3OD)
20 10 0 -10 -20 -30 -40 -50 -60 -70 -80 -90 -100 -110 -120 -130 -140 -150 -160 -170 -180 -190
f1 (ppm)

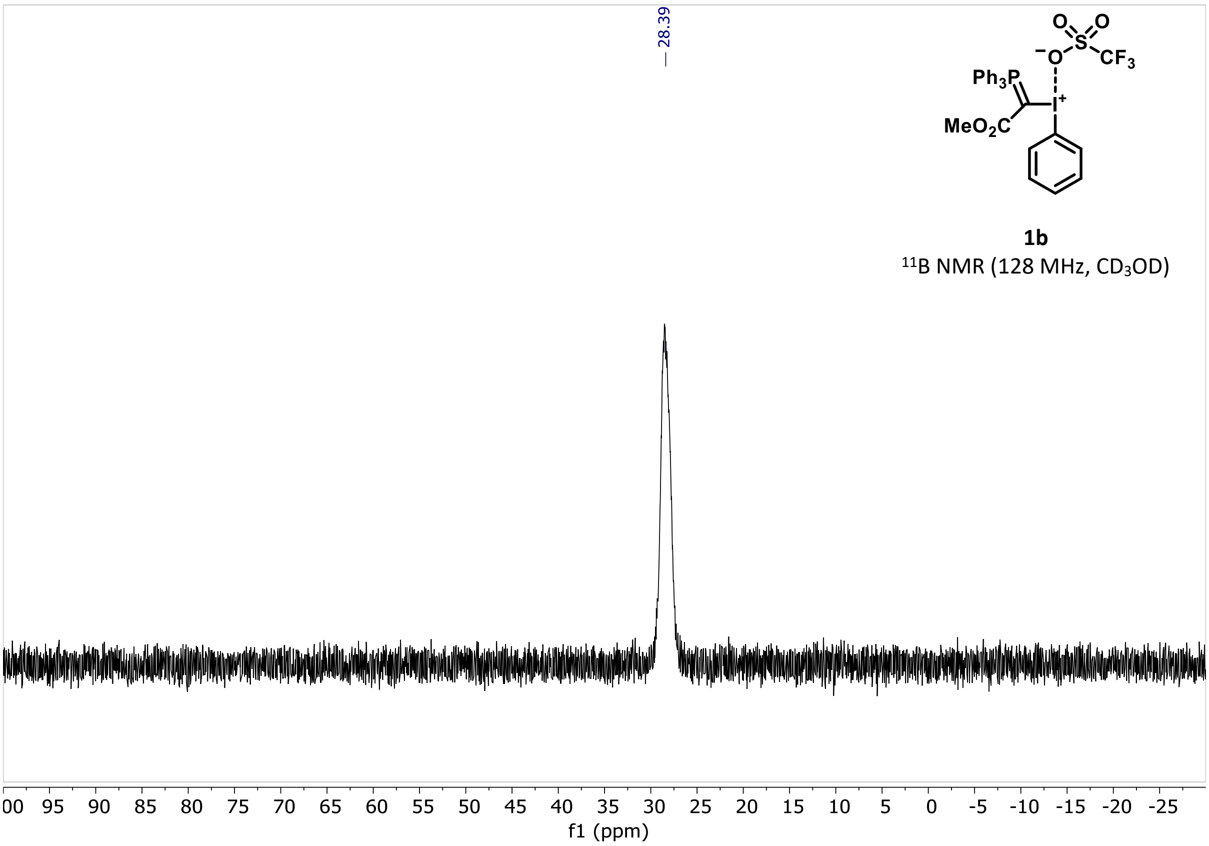

Phenyliodonium(methyl (triphenylphosphoranylidene)acetate) chloride (1c)

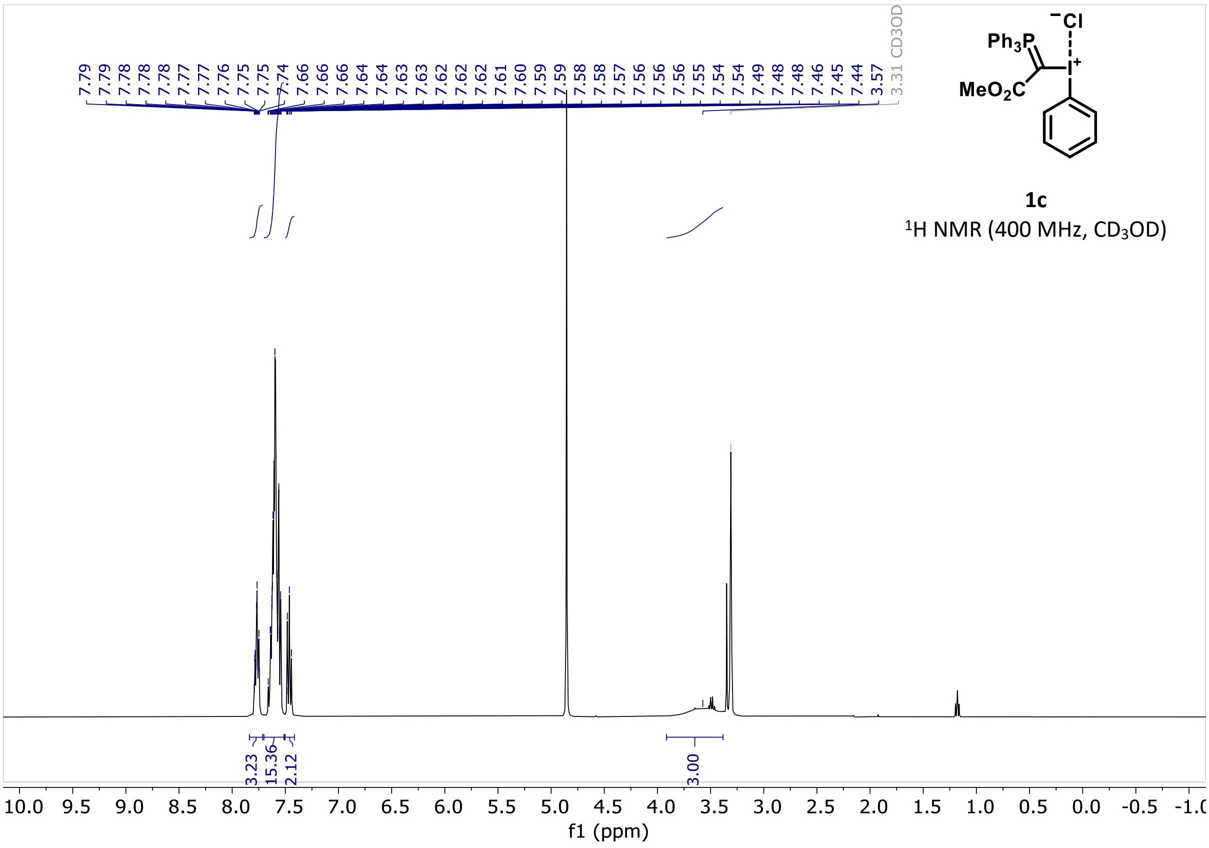

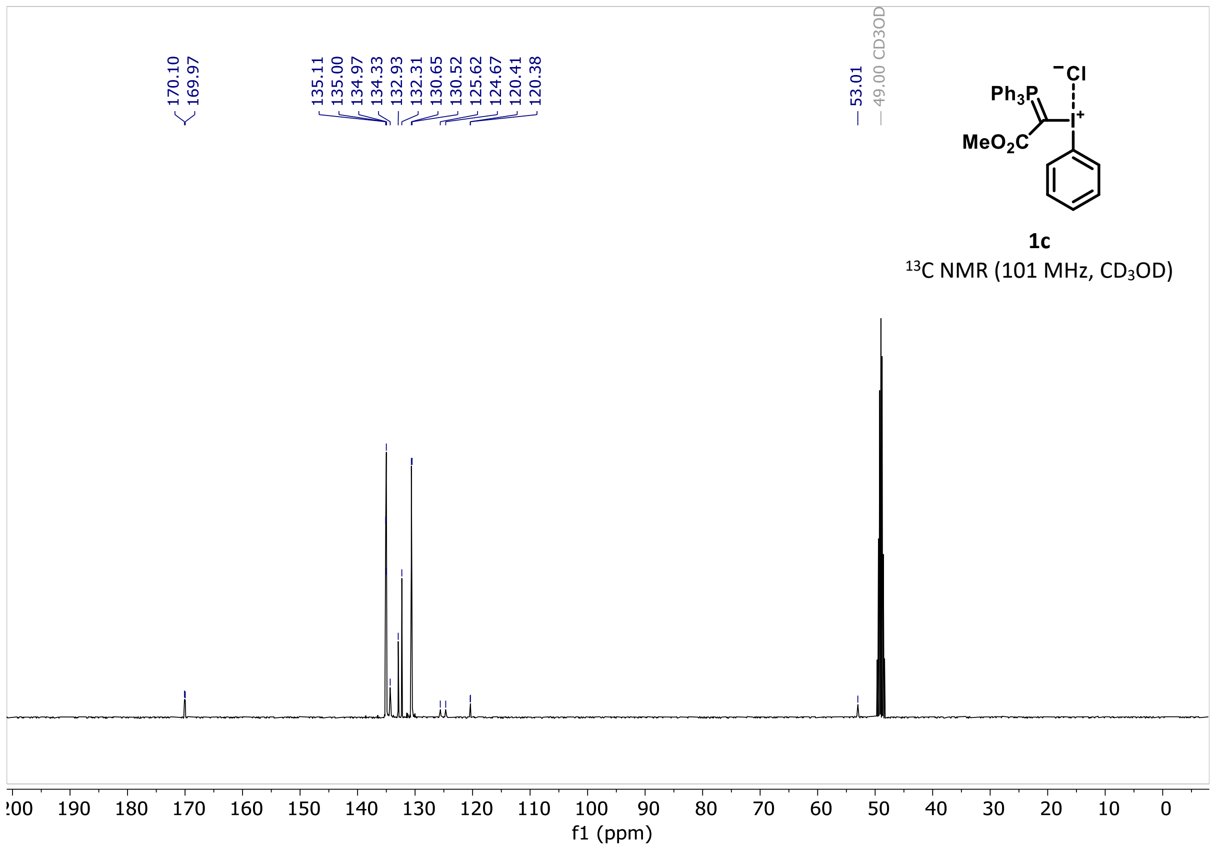

170.10
169.97
135.11
135.00
134.97
134.33
132.93
132.31
130.65
130.52
125.62
124.67
120.41
120.38
53.01
49.00 CD3OD
Ph3P
Cl
I+
MeO2C
1c
13C NMR (101 MHz, CD3OD)
f1 (ppm)

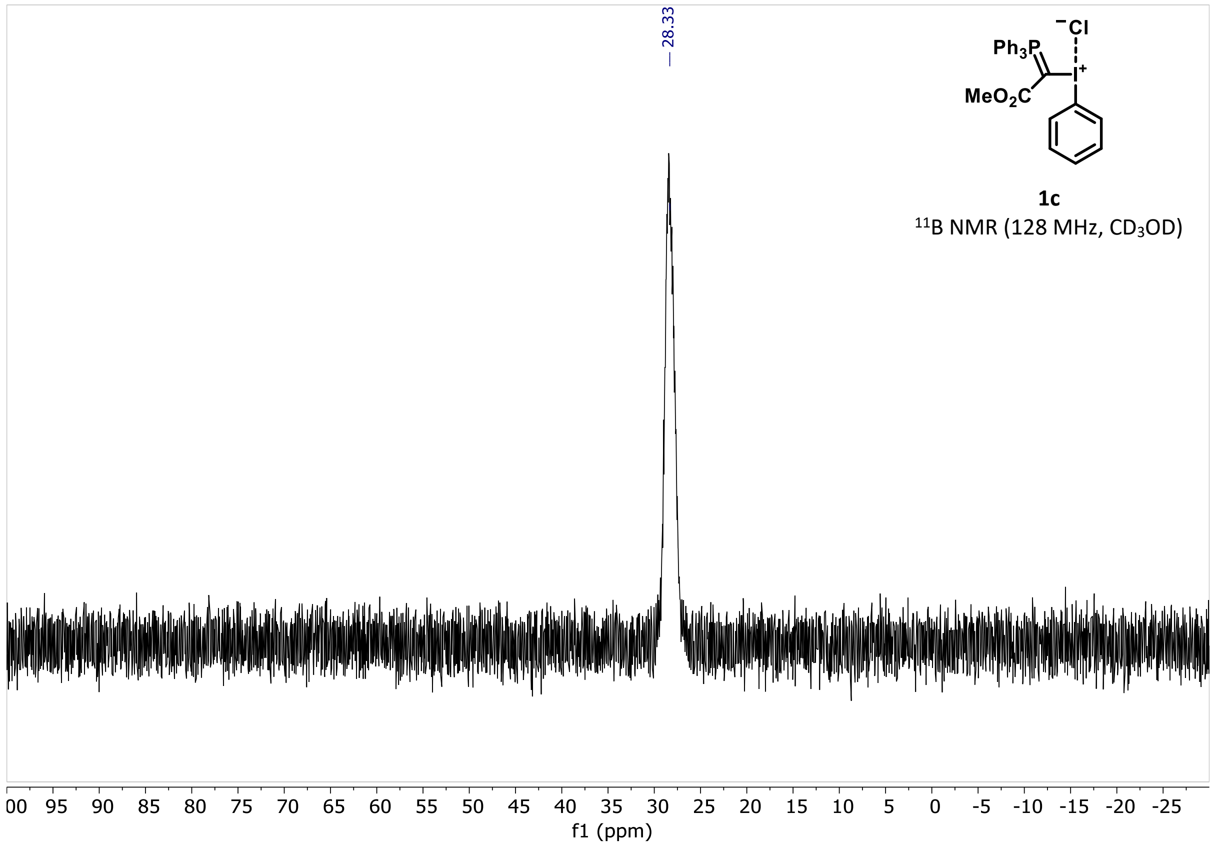

28.33
Ph3P
Cl
I+
MeO2C
1c
11B NMR (128 MHz, CD3OD)
f1 (ppm)

Phenyliodonium(methyl (triphenylphosphoranylidene)acetate) nitrate (1d)

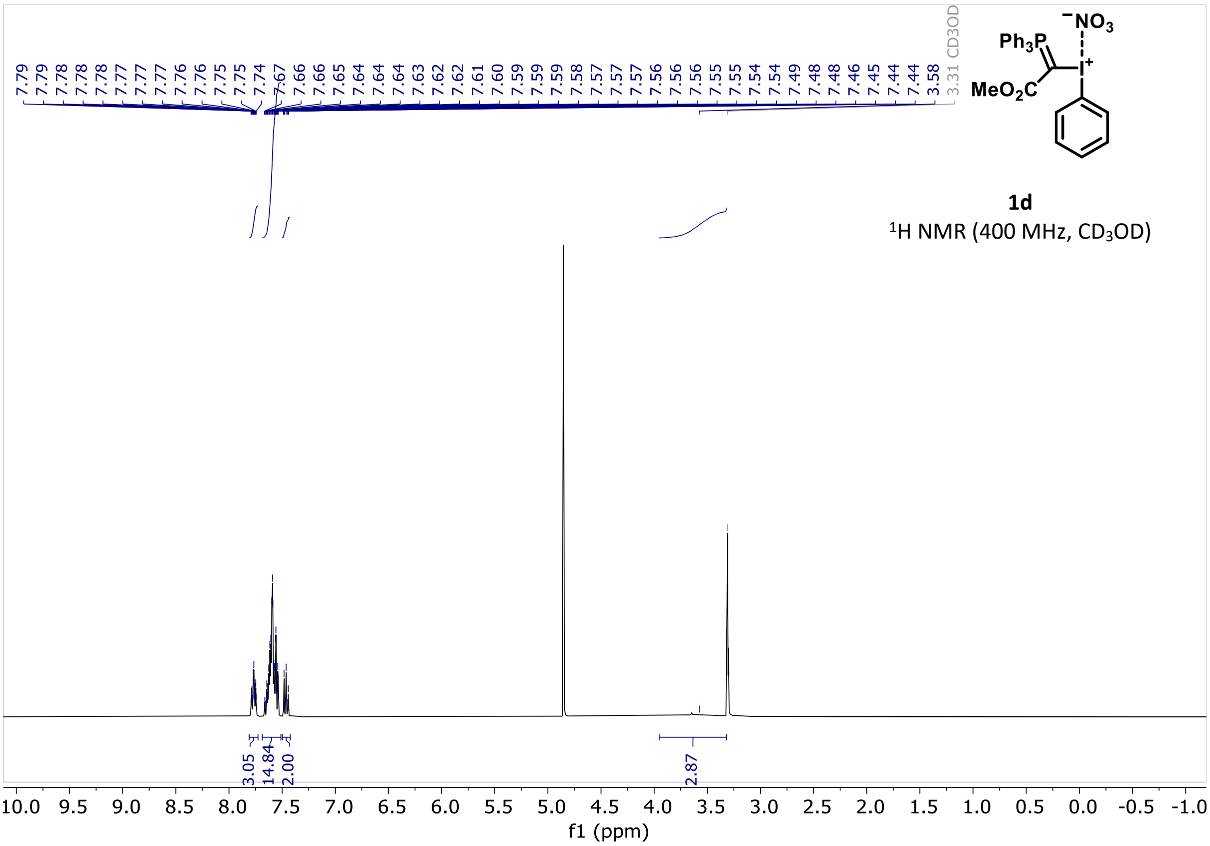

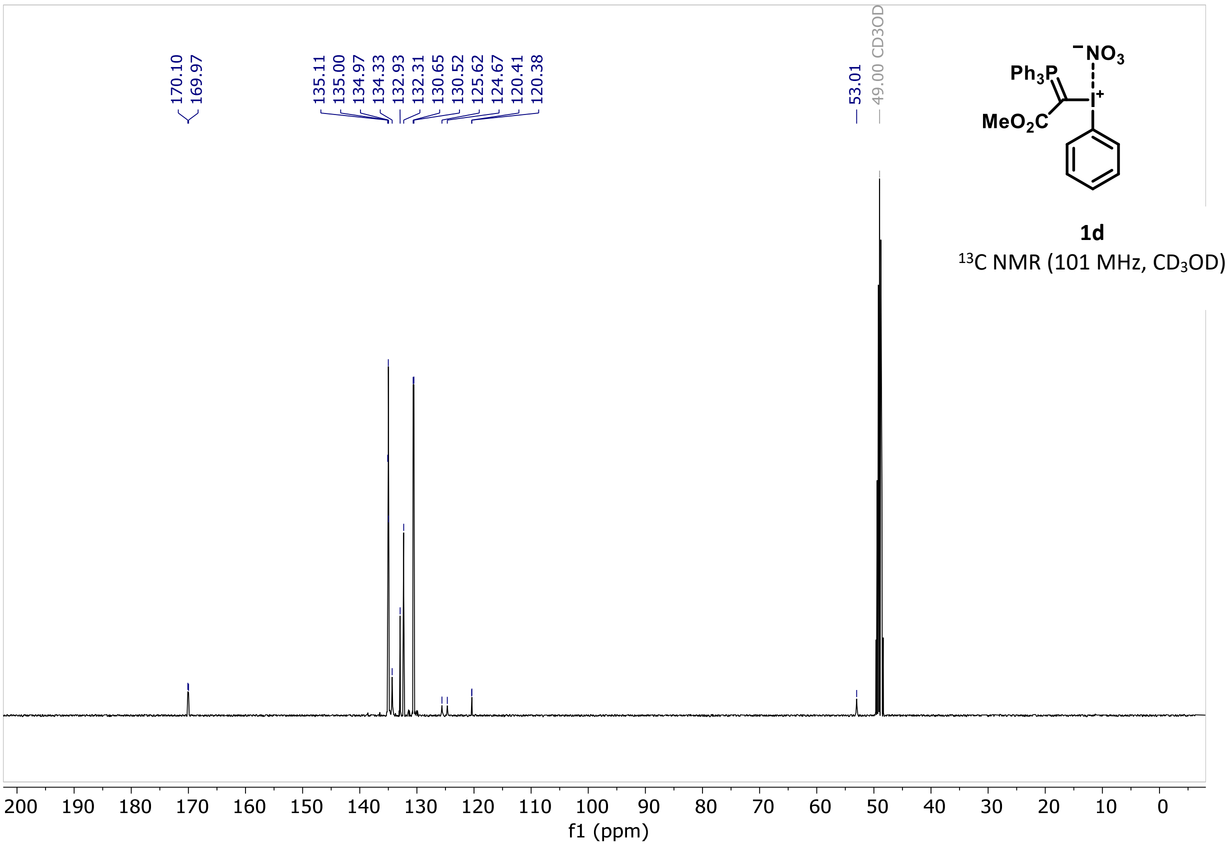

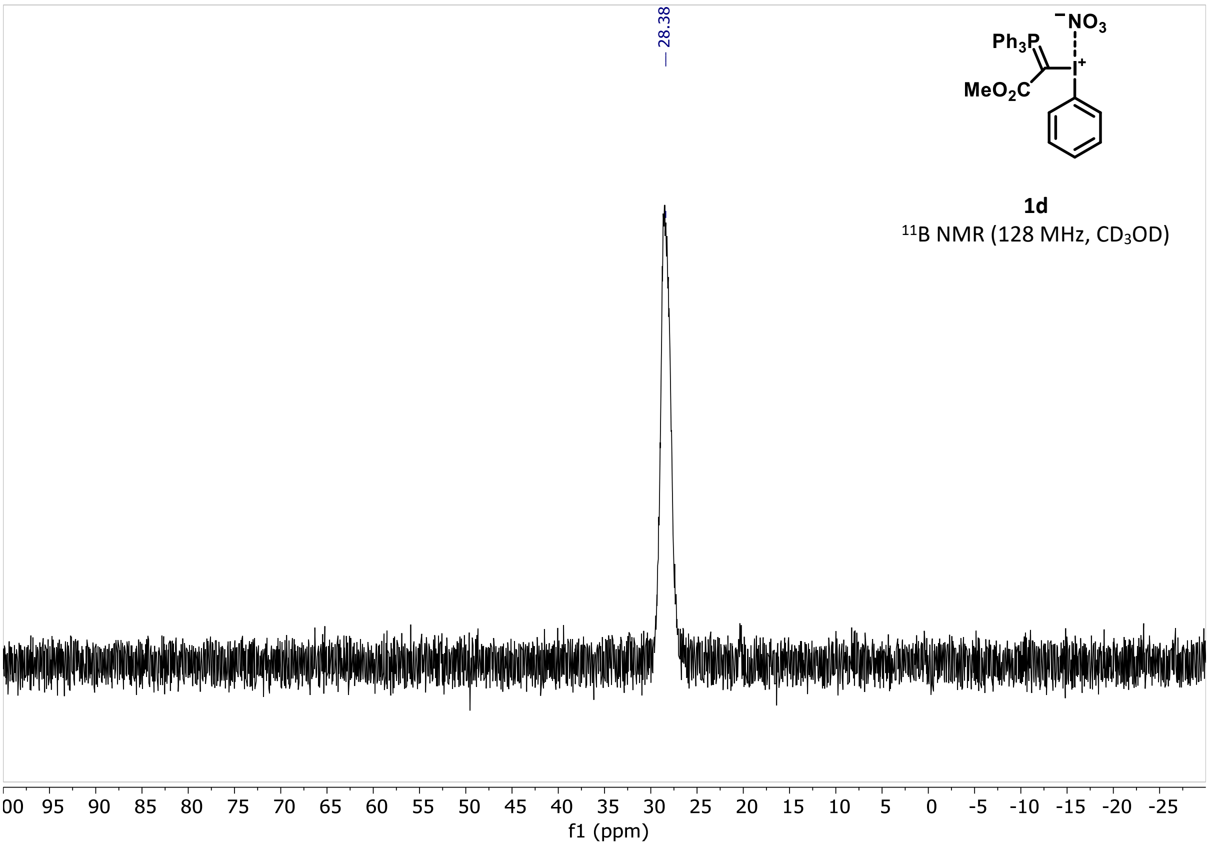

Phenyliodonium(methyl (triphenylphosphoranylidene)acetate) tosylate (1e)

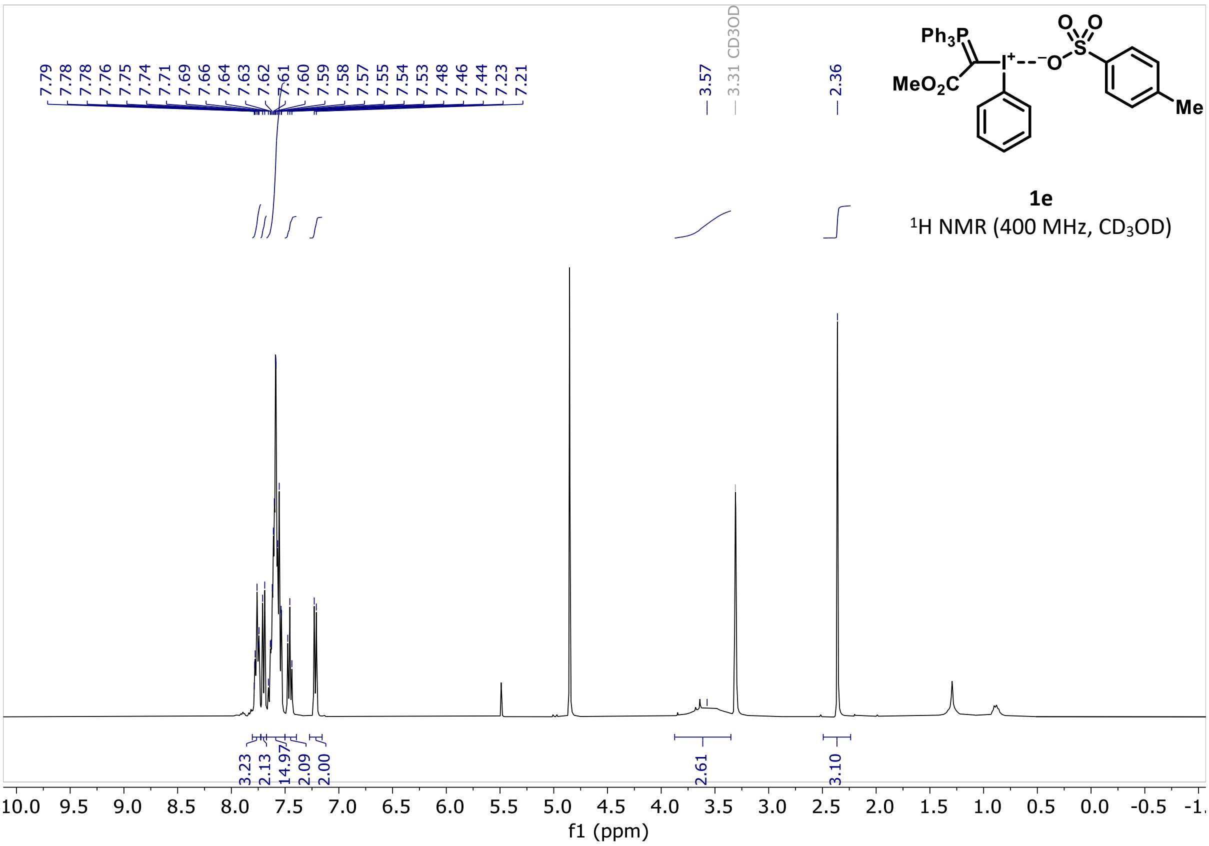

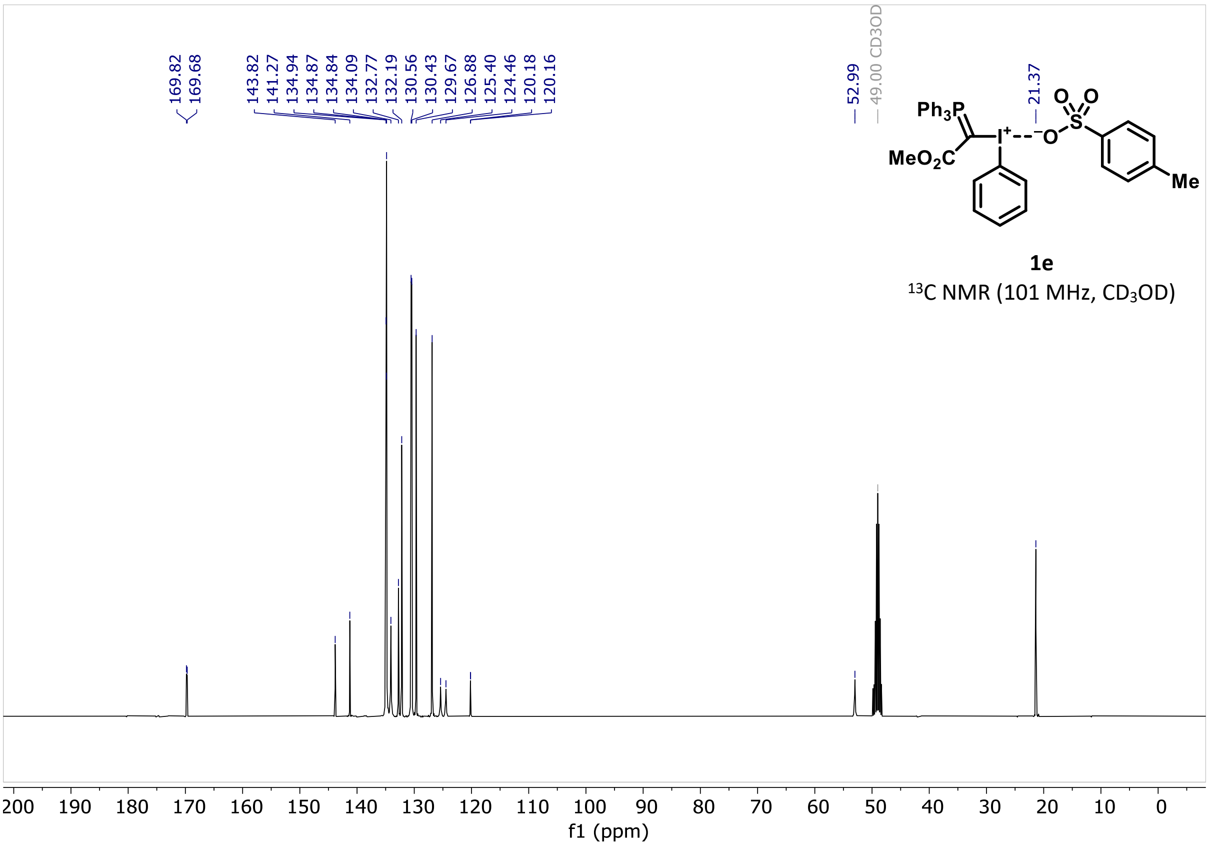
169.82
169.68
143.82
141.27
134.94
134.87
134.84
134.09
132.77
132.19
130.56
130.43
129.67
126.88
125.40
124.46
120.18
120.16
52.99
49.00 CD3OD
21.37
Ph3P
MeO2C
I+
O
S
O O
Me
1e
13C NMR (101 MHz, CD3OD)
200 190 180 170 160 150 140 130 120 110 100 90 80 70 60 50 40 30 20 10 0
f1 (ppm)

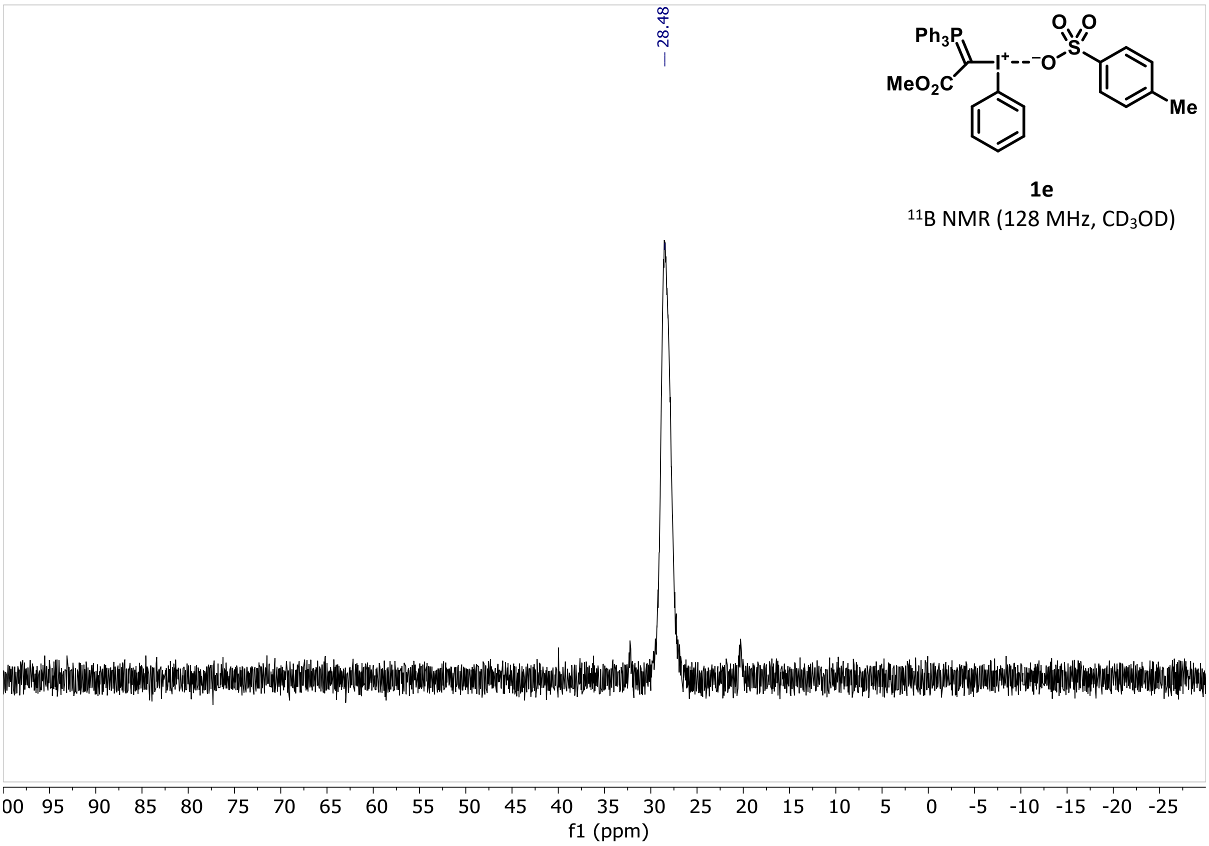
28.48
Ph3P
MeO2C
I+
O
S
O O
Me
1e
11B NMR (128 MHz, CD3OD)
00 95 90 85 80 75 70 65 60 55 50 45 40 35 30 25 20 15 10 5 0 -5 -10 -15 -20 -25
f1 (ppm)

1-(Hydroxy(tosyloxy)iodo)-4-methylbenzene (S1)

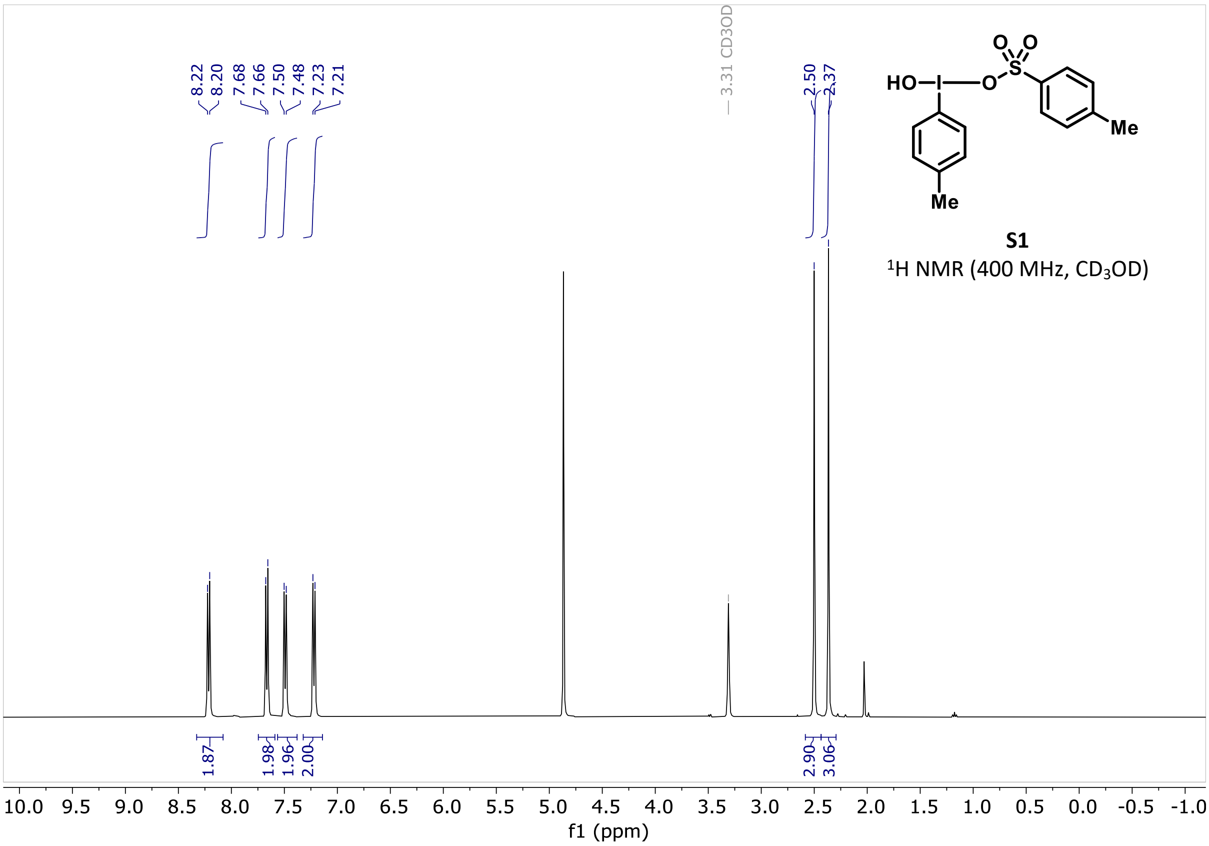

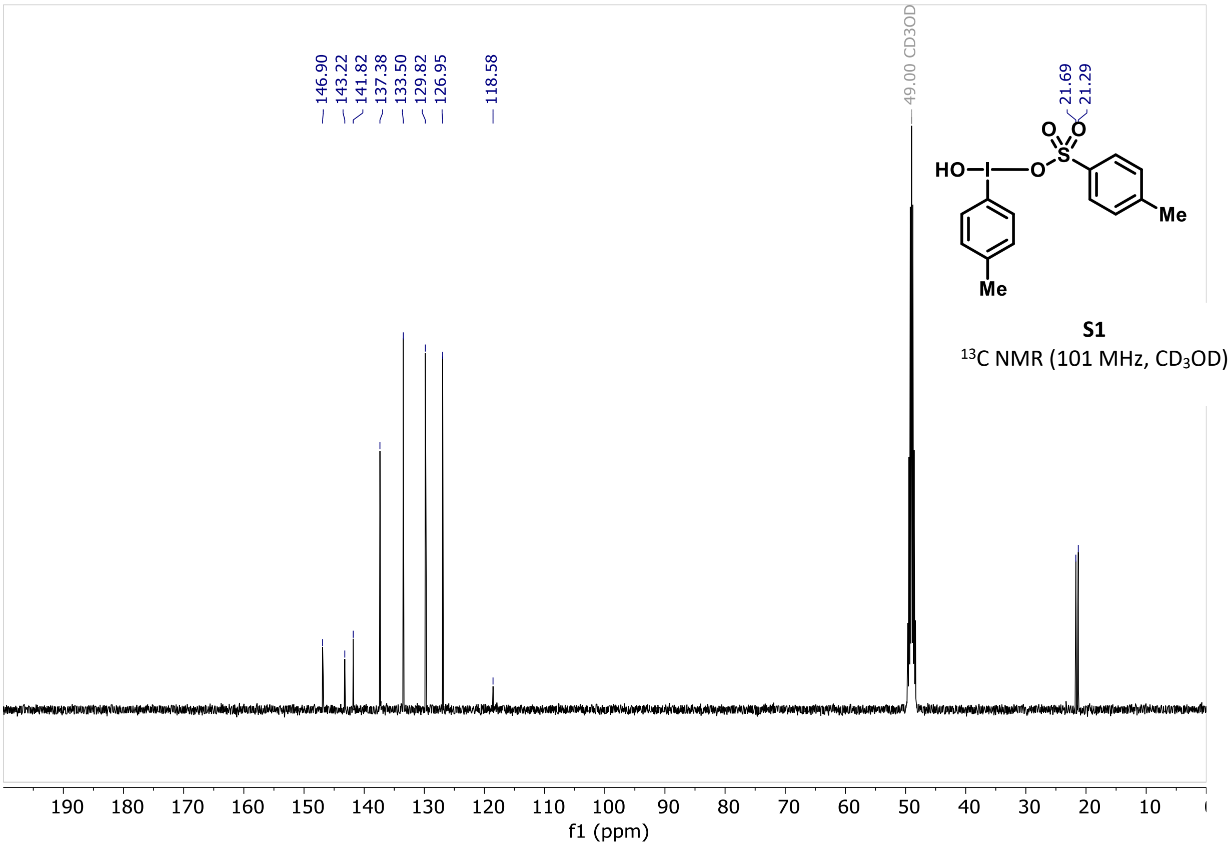

4-Methylphenyliodonium(methyl (triphenylphosphoranylidene)acetate) tosylate (1f)

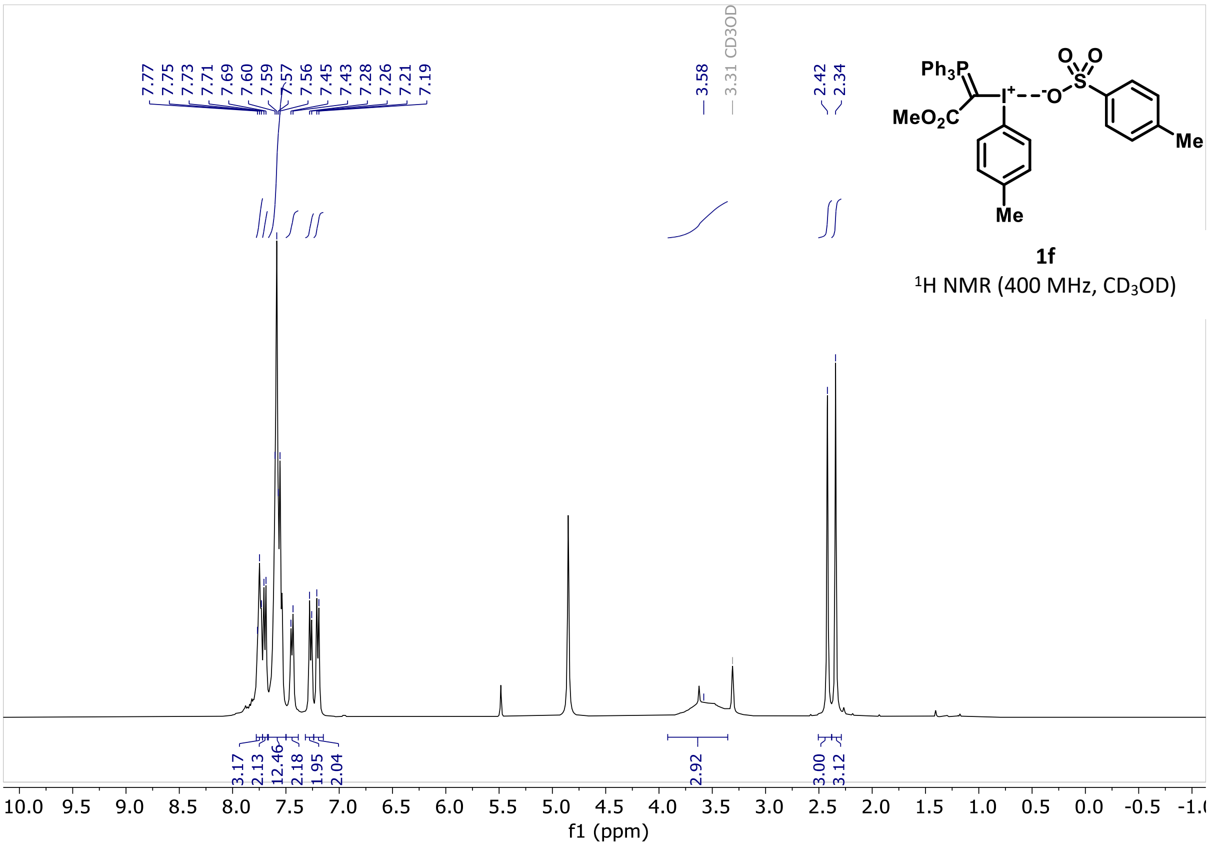

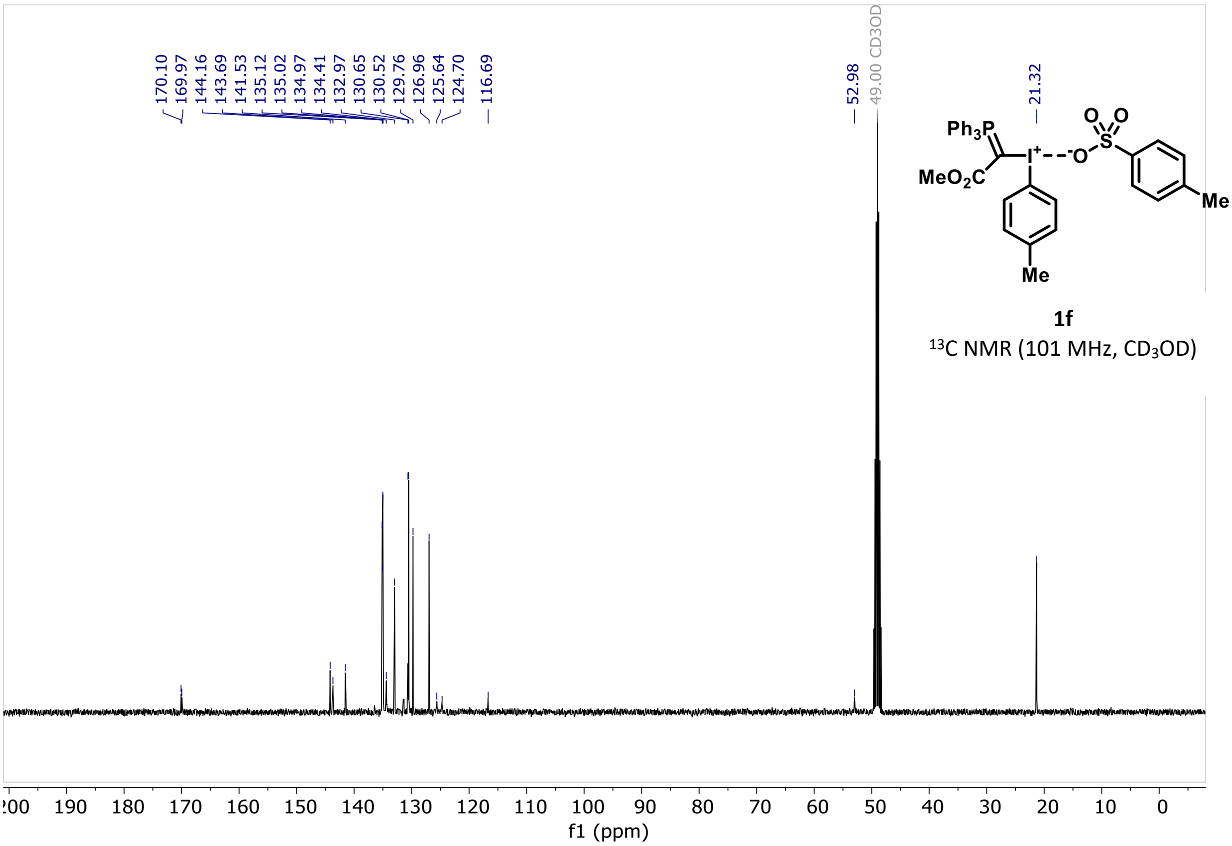

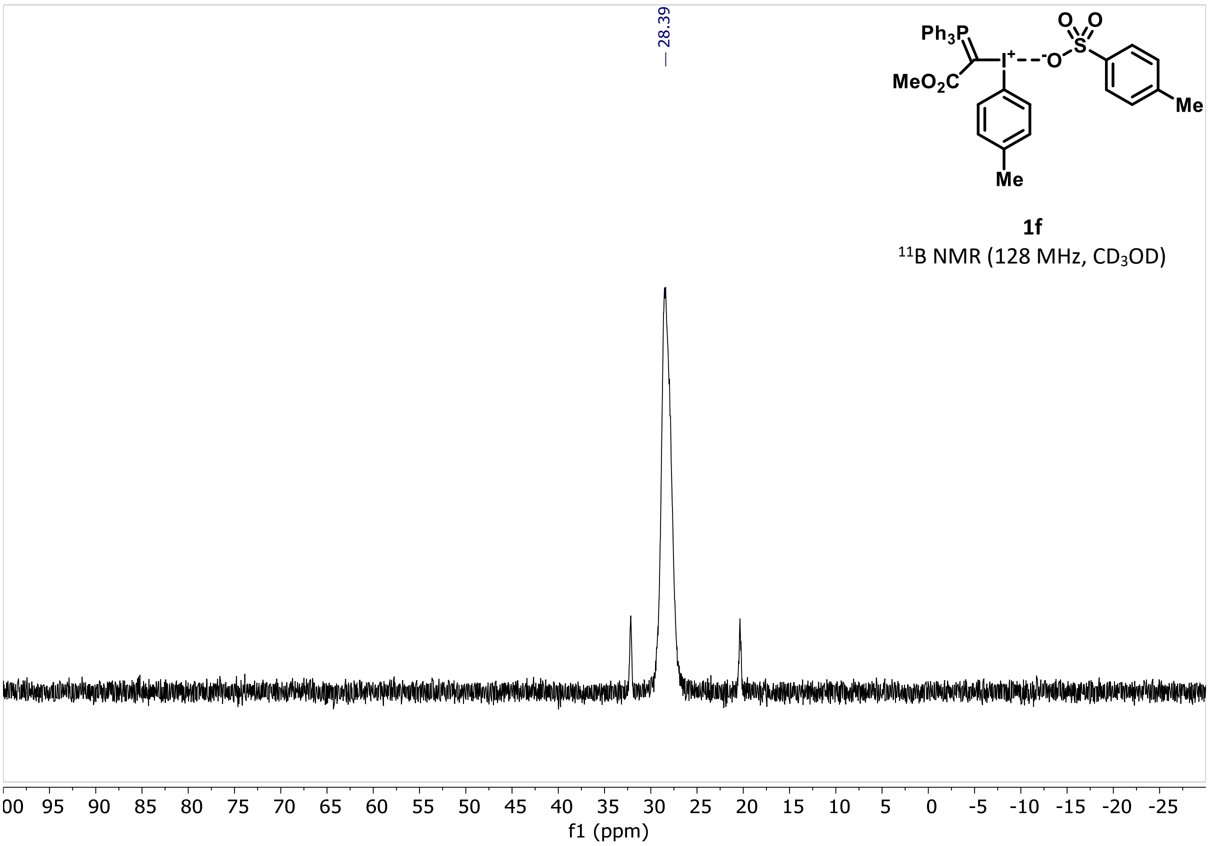

1f
^{11}B NMR (128 MHz, CD_3OD)

1-(Hydroxy(tosyloxy)iodo)-4-fluorobenzene (S2)

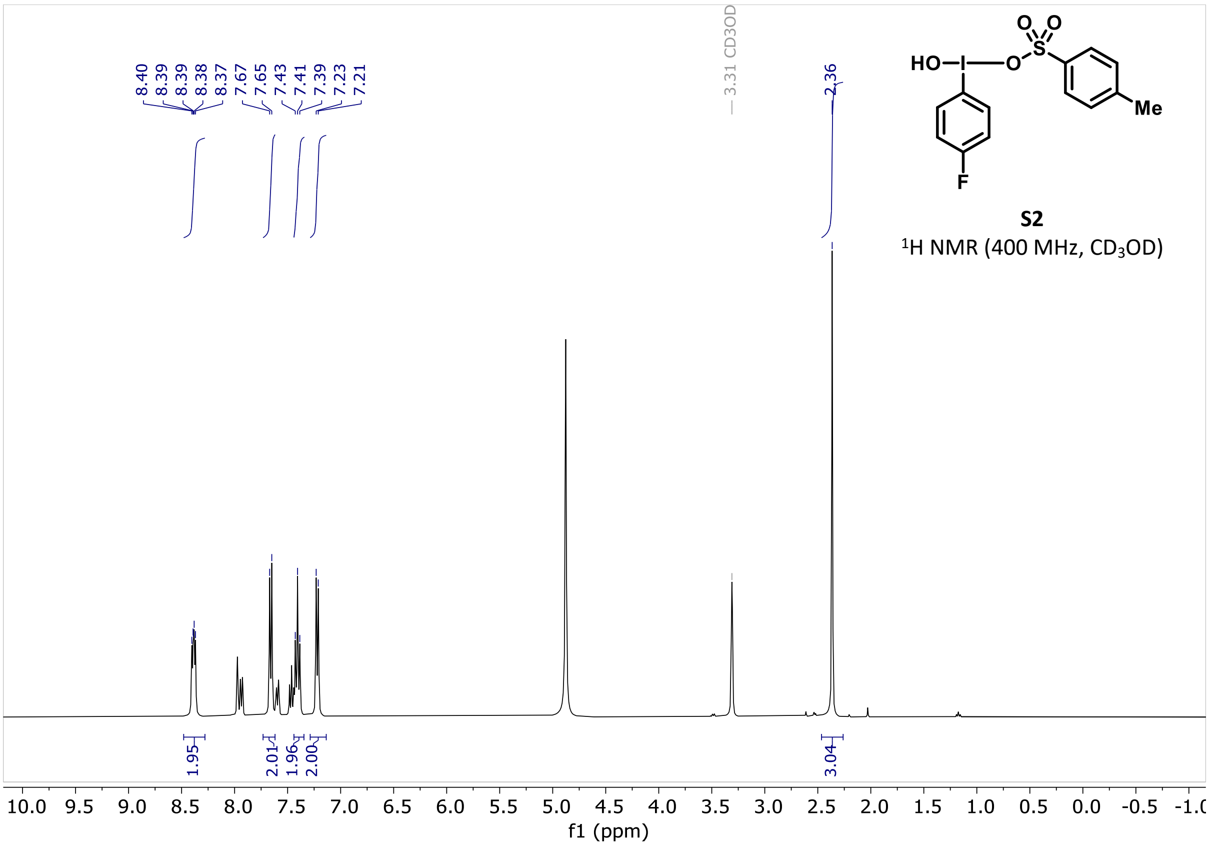

S2
^{1}H NMR (400 MHz, CD_3OD)

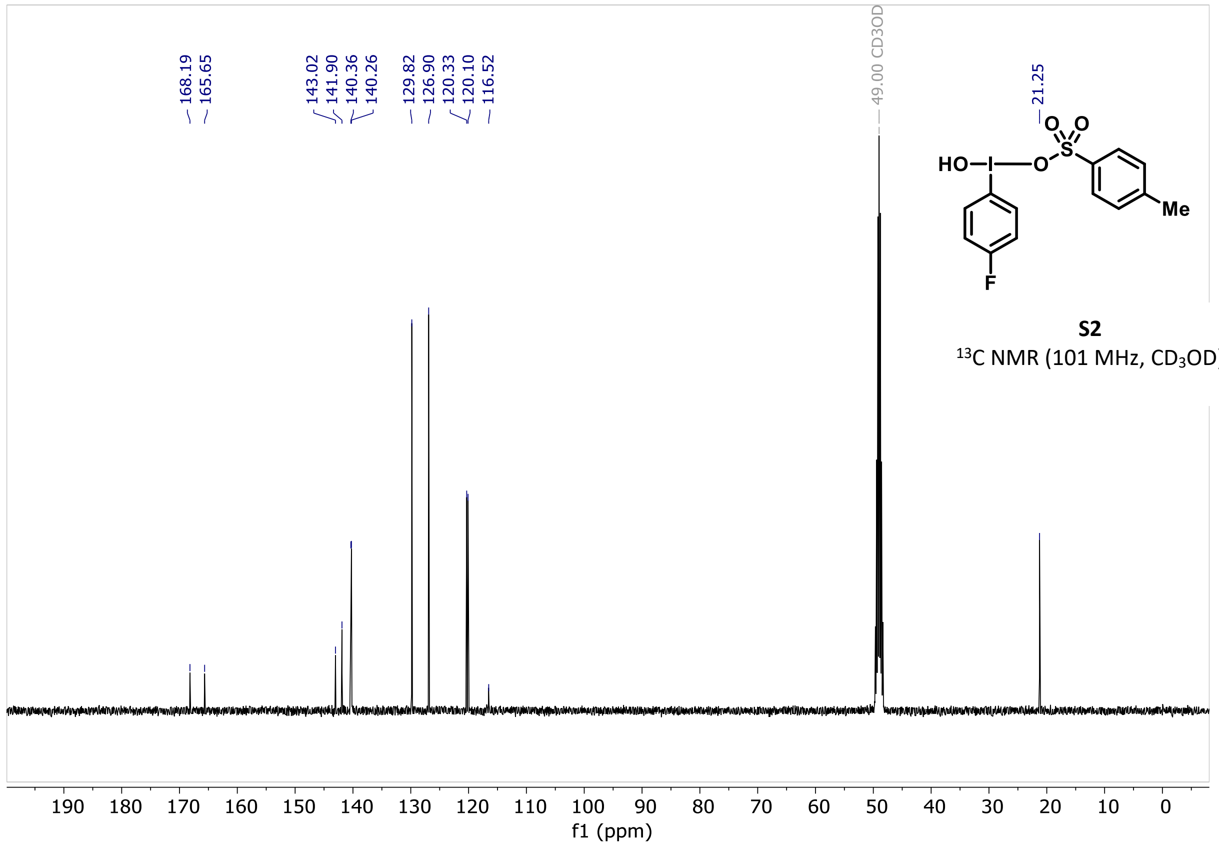

S2

^{13}C NMR (101 MHz, CD_3OD)

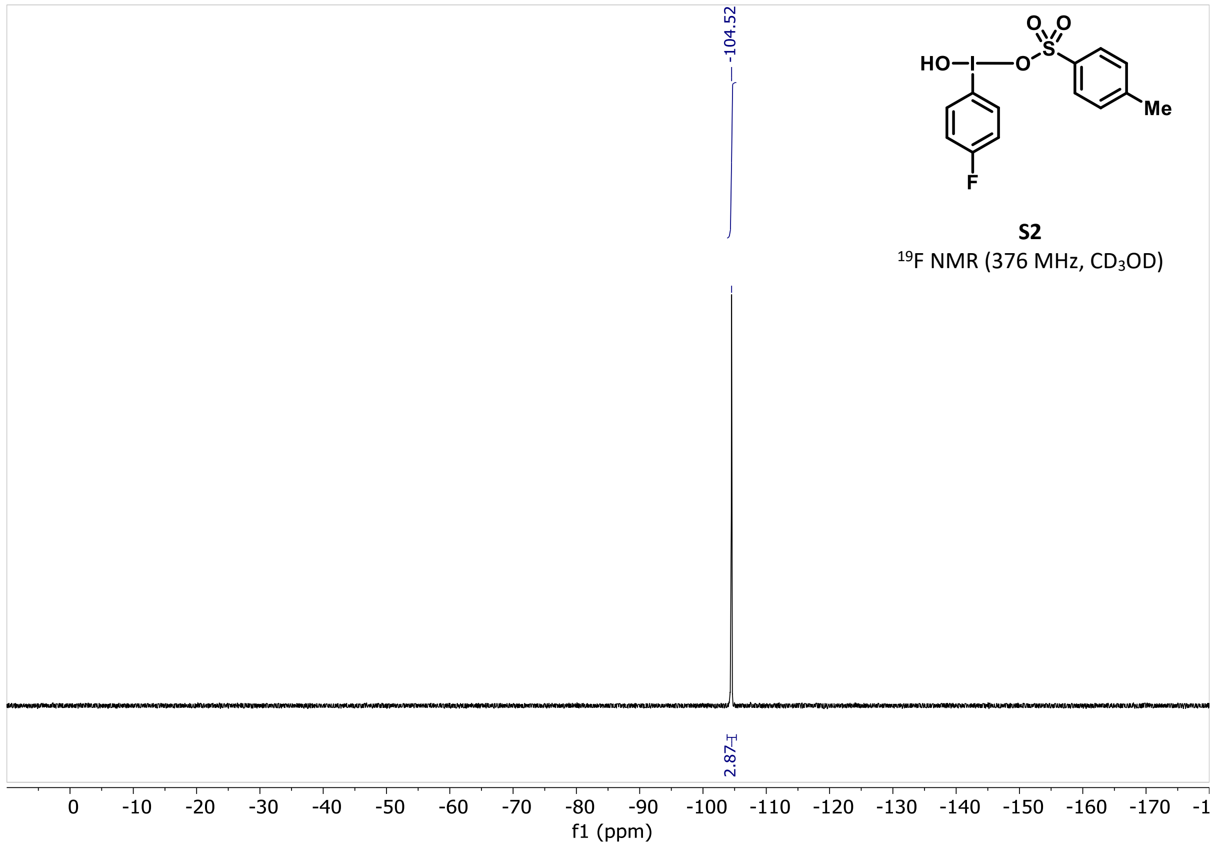

S2

^{19}F NMR (376 MHz, CD_3OD)

4-Fluoromethylphenyliodonium(methyl (triphenylphosphoranylidene)acetate) tosylate (1g)

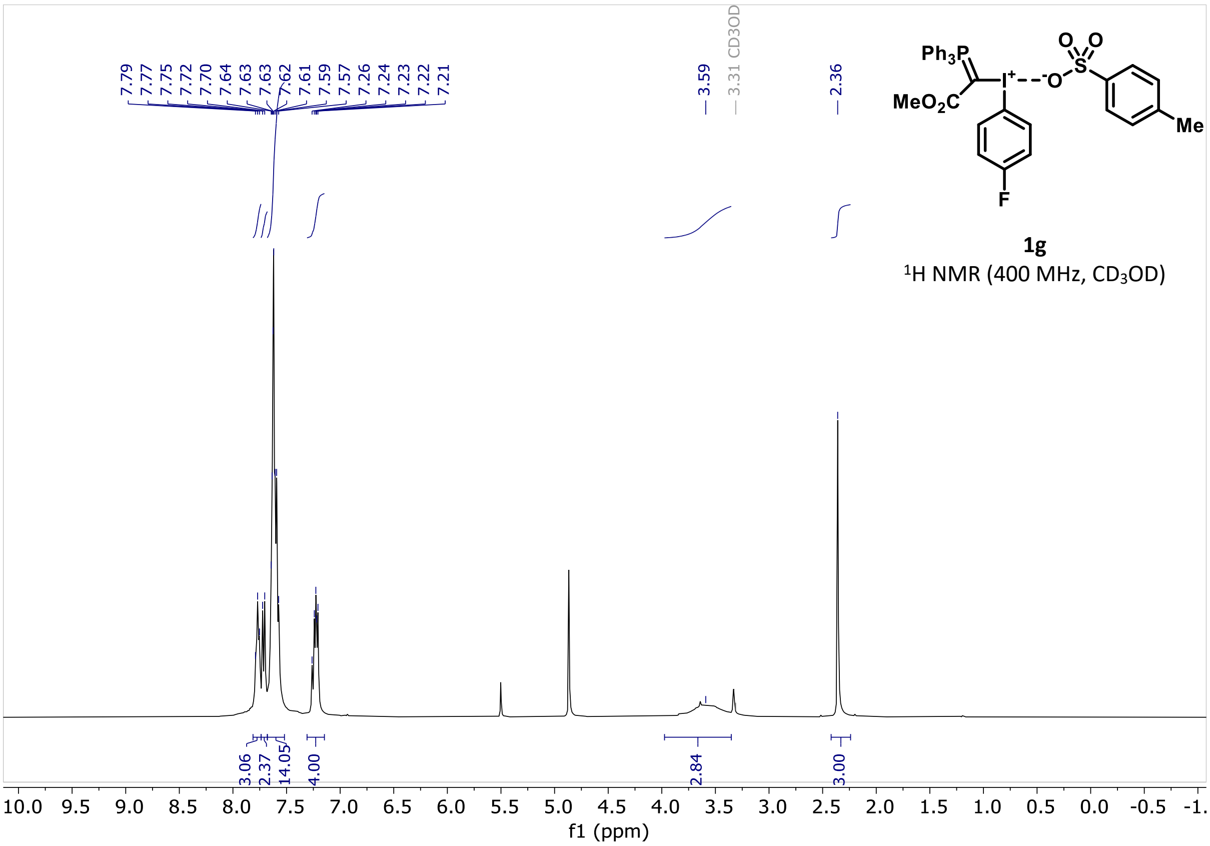

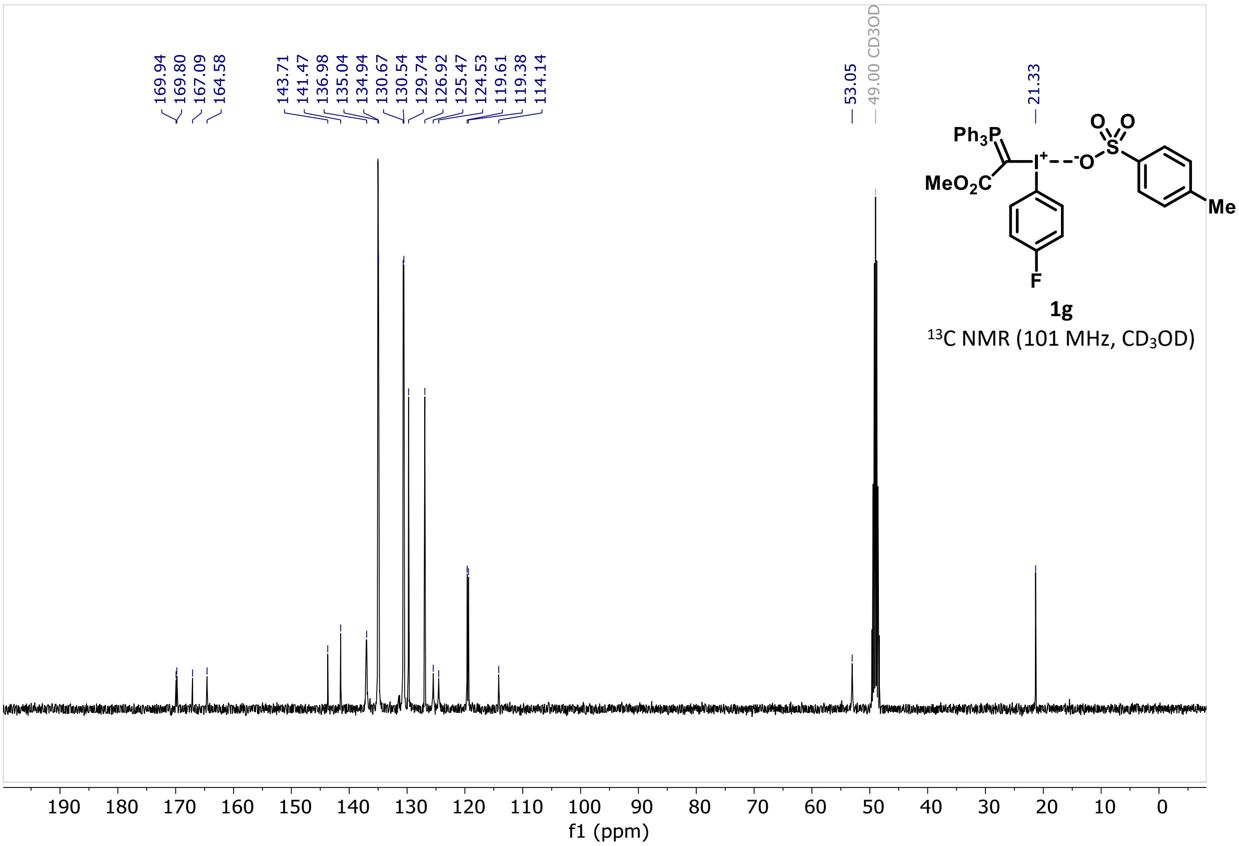

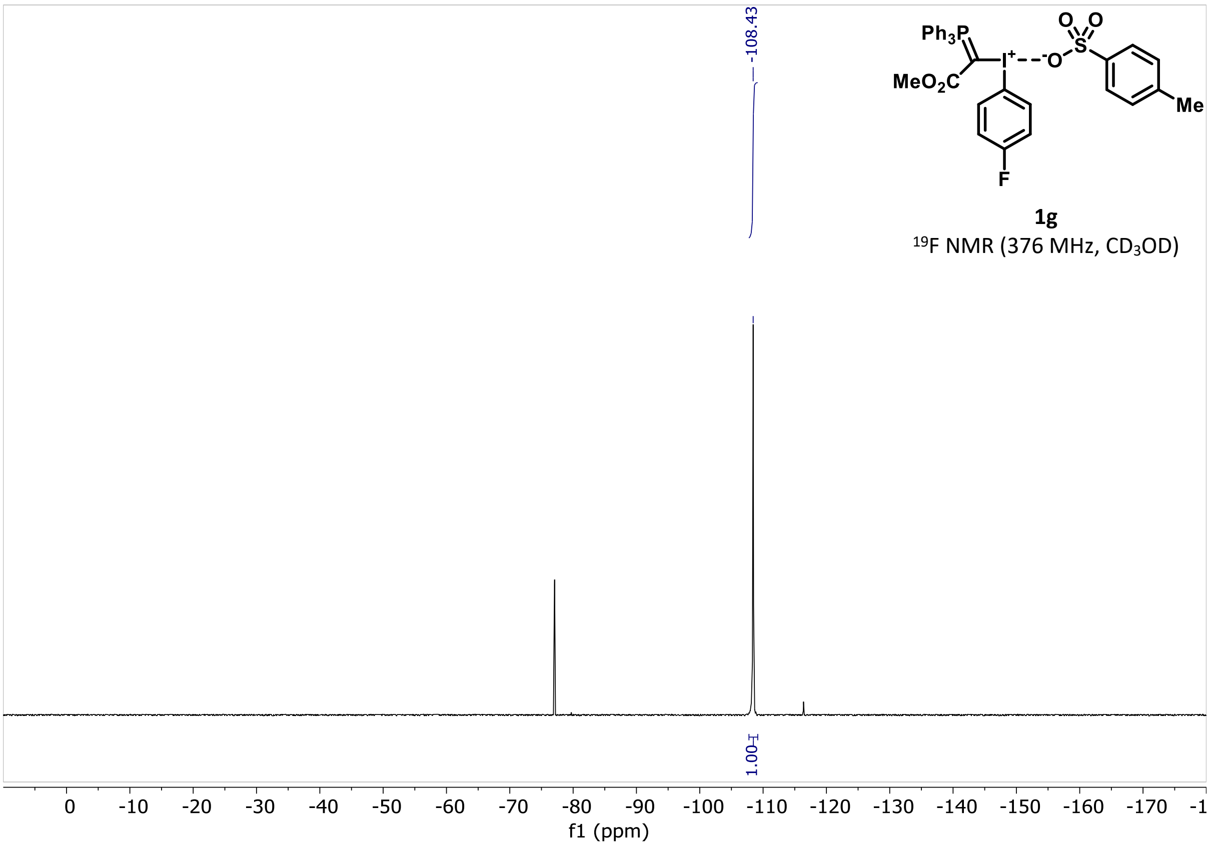

1g

^{19}F NMR (376 MHz, CD_3OD)

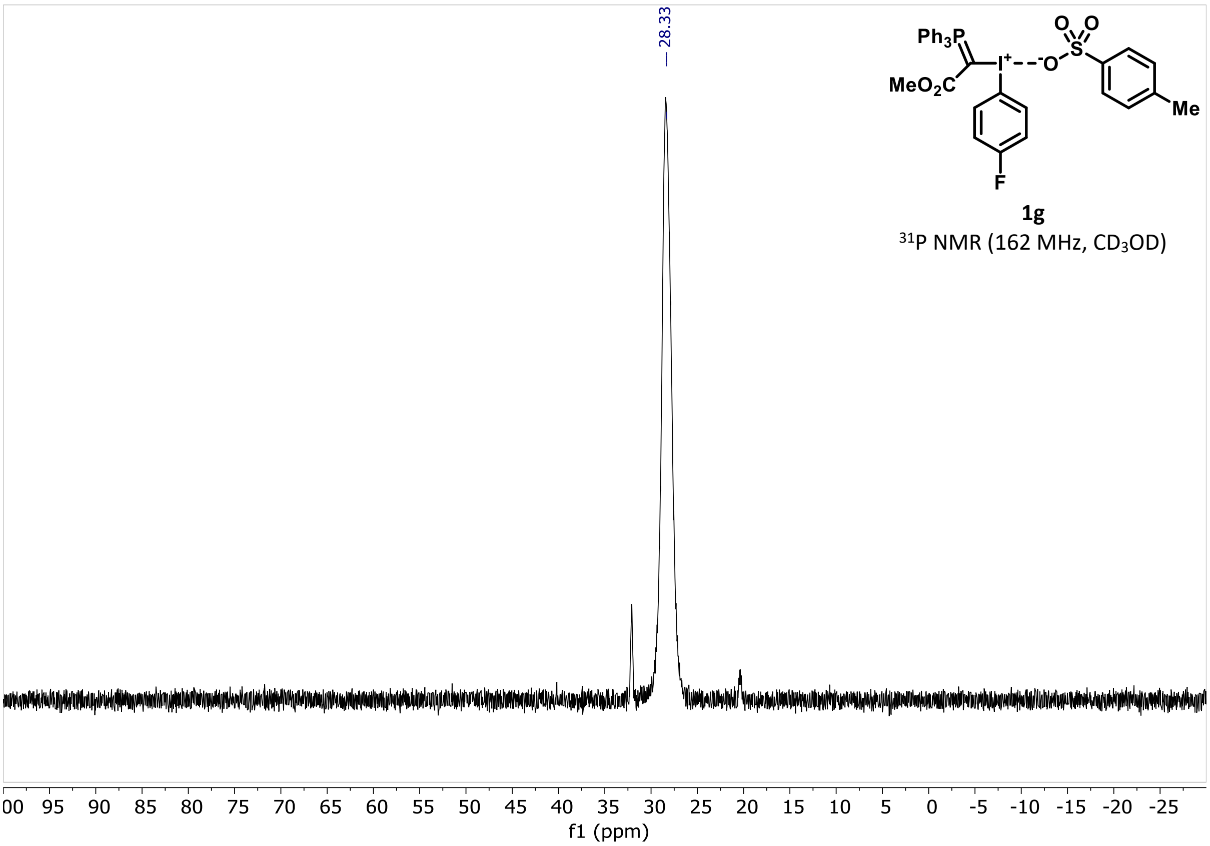

1g

^{31}P NMR (162 MHz, CD_3OD)

1-(Hydroxy(tosyloxy)iodo)-4-trifluoromethylbenzene (S3)

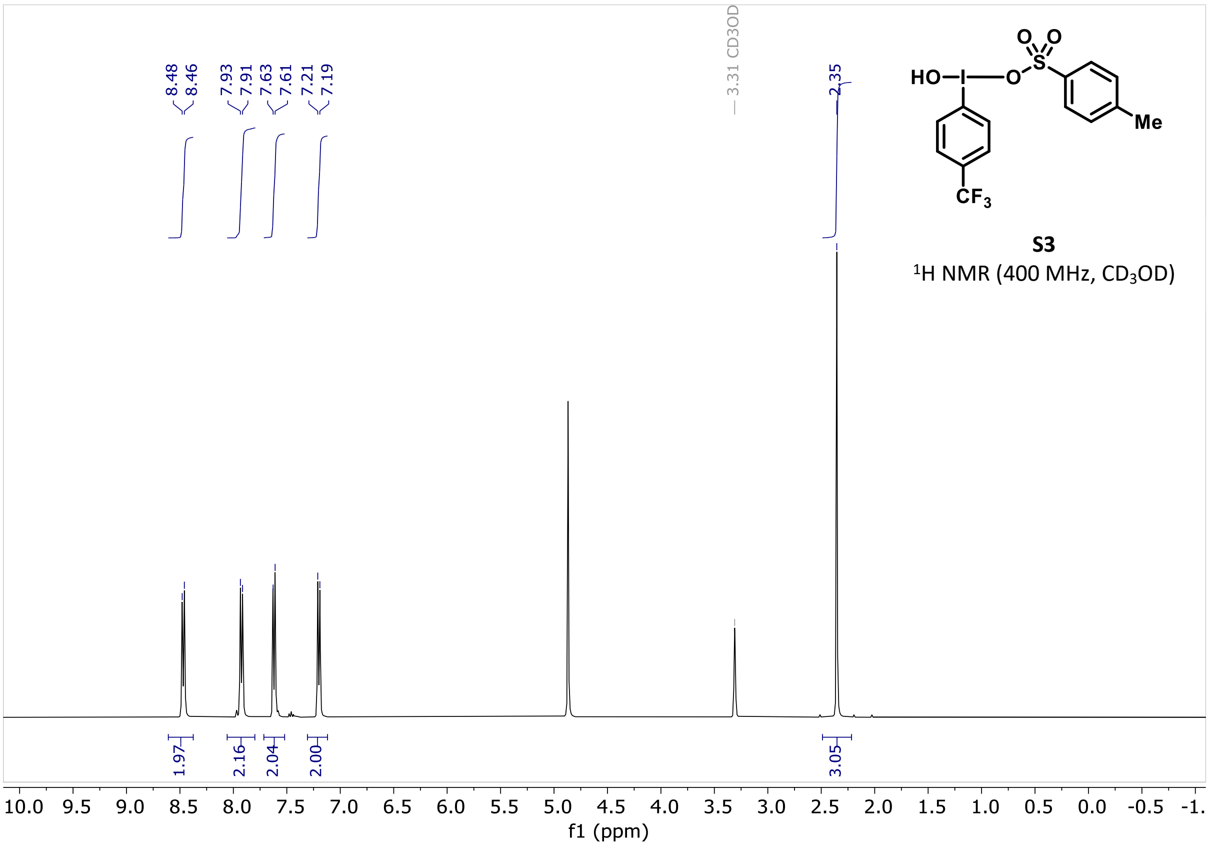

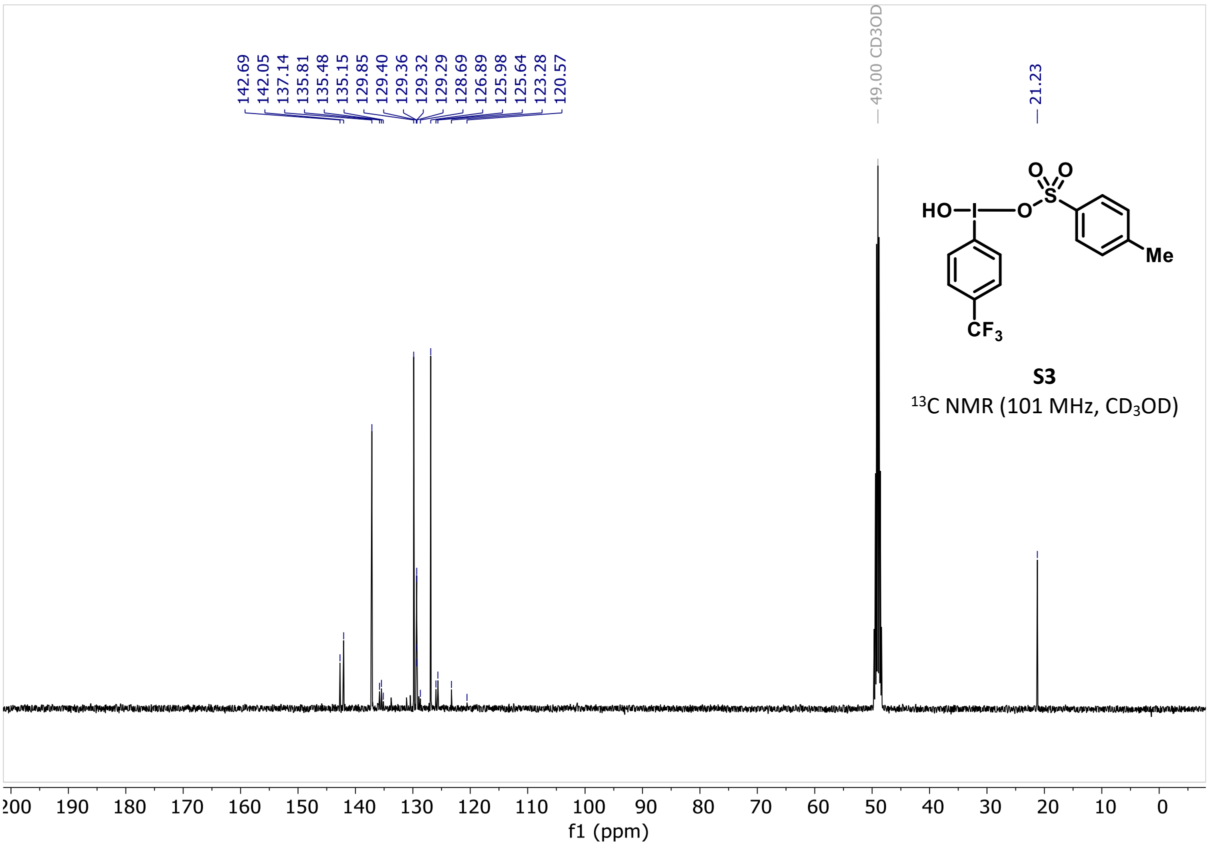

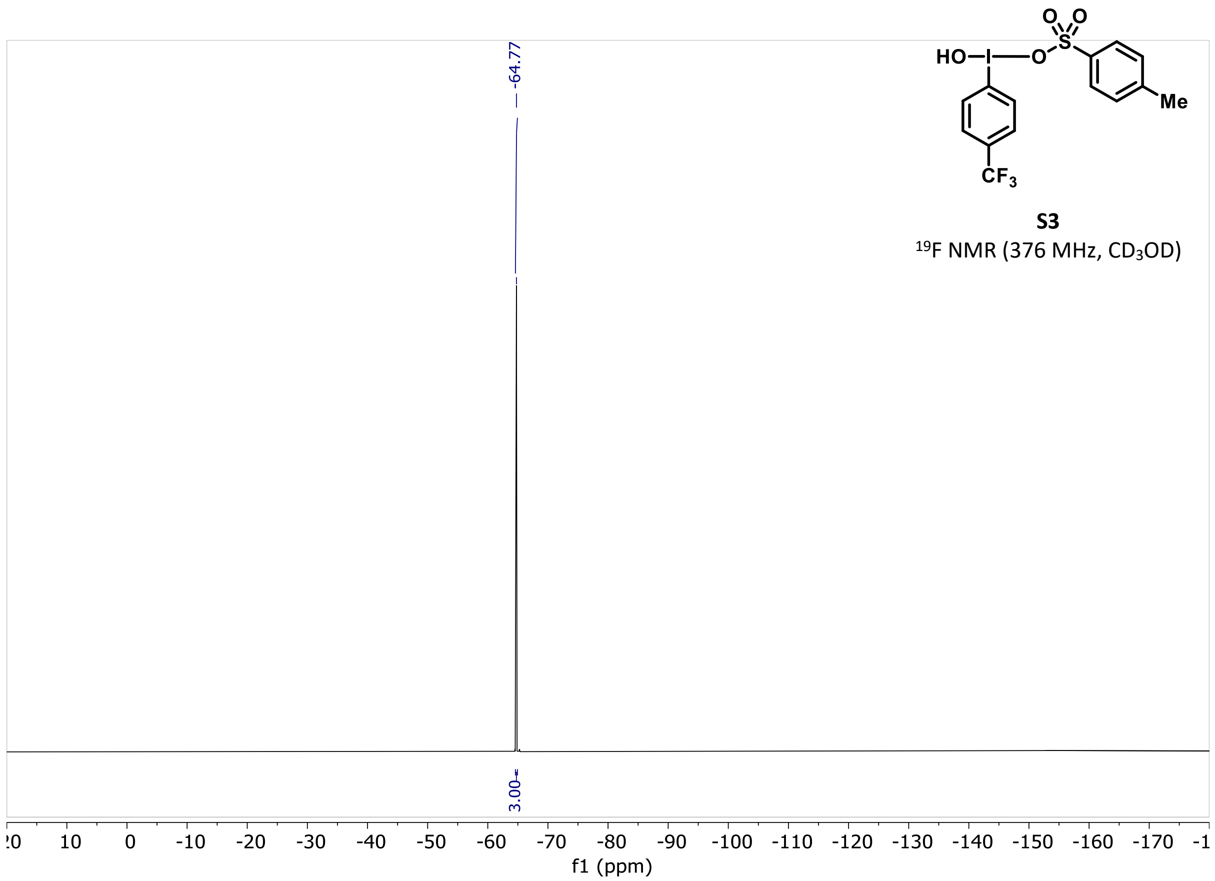

4-Trifluoromethylphenyliodonium(methyl (triphenylphosphoranylidene)acetate) tosylate (1h)

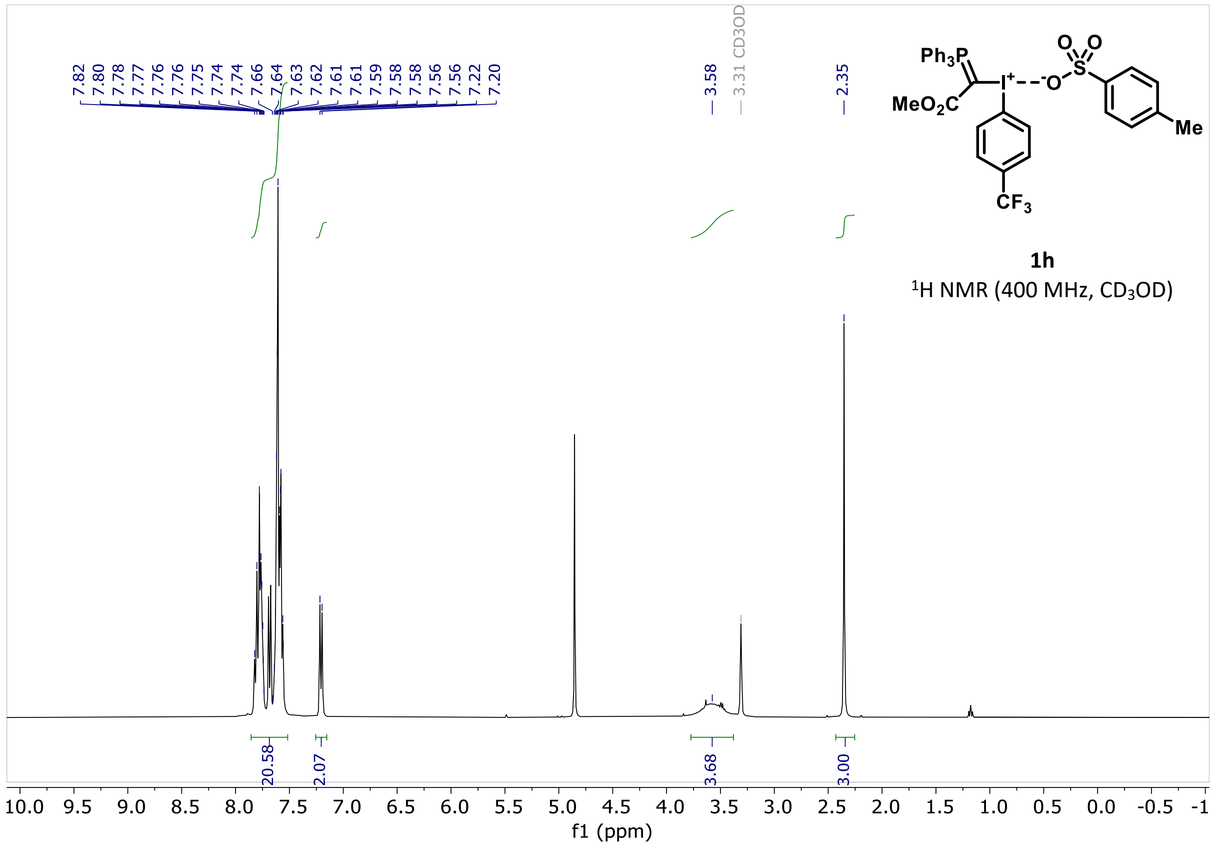

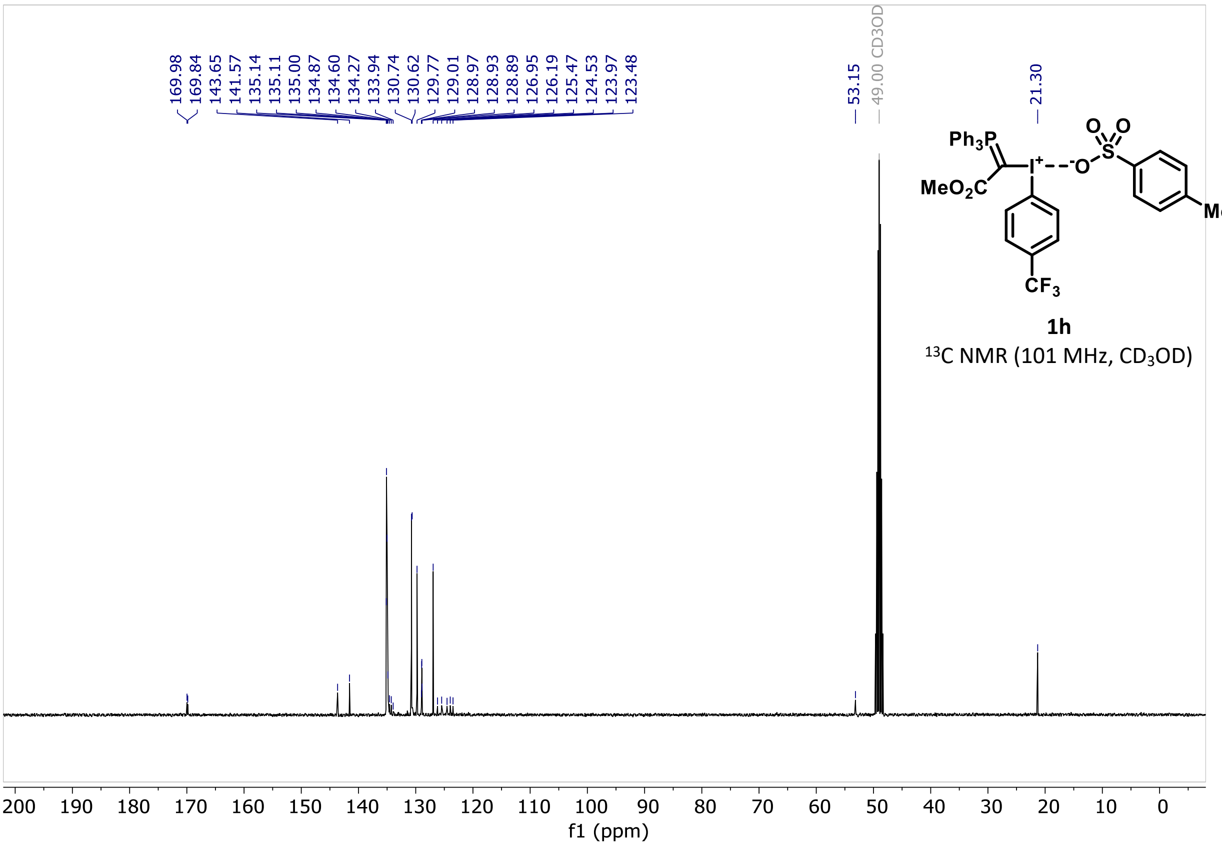
169.98
169.84
143.65
141.57
135.14
135.11
135.00
134.87
134.60
134.27
133.94
130.74
130.62
129.77
129.01
128.97
128.93
128.89
126.95
126.19
125.47
124.53
123.97
123.48
53.15
49.00 CD3OD
21.30
Ph3P
MeO2C
I+
O
S
O O
Me
CF3
1h
13C NMR (101 MHz, CD3OD)
200 190 180 170 160 150 140 130 120 110 100 90 80 70 60 50 40 30 20 10 0
f1 (ppm)

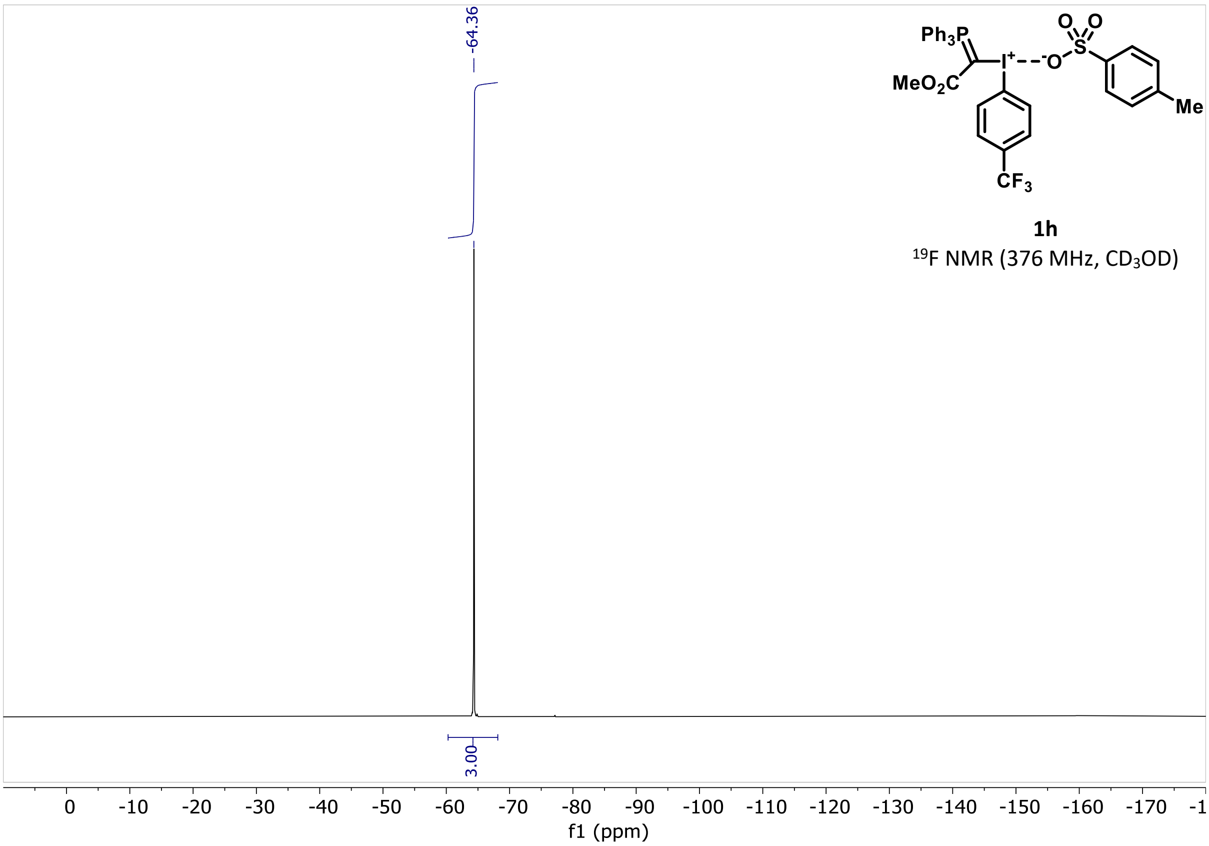
-64.36
Ph3P
MeO2C
I+
O
S
O O
Me
CF3
1h
19F NMR (376 MHz, CD3OD)
3.00
0 -10 -20 -30 -40 -50 -60 -70 -80 -90 -100 -110 -120 -130 -140 -150 -160 -170 -1
f1 (ppm)

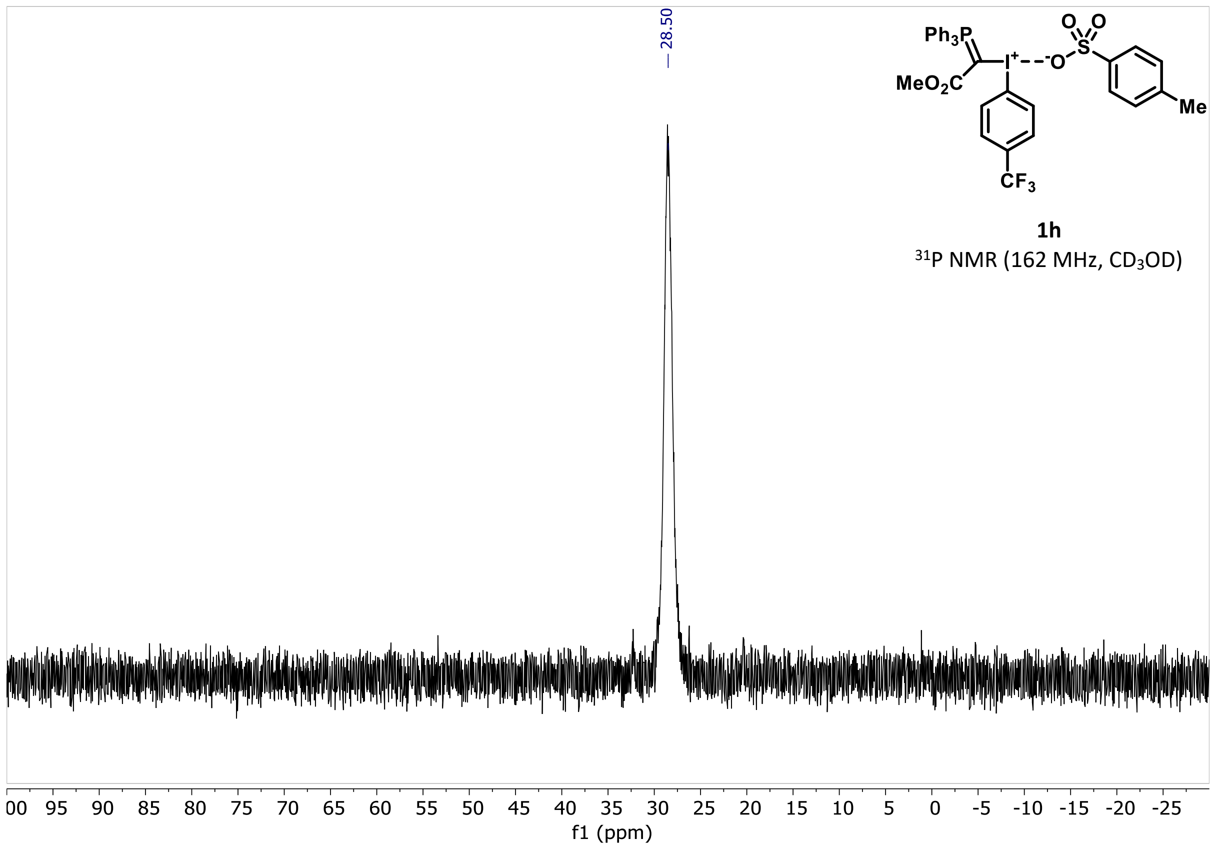

1-(Hydroxy(tosyloxy)iodo)-4-nitrobenzene (S4)

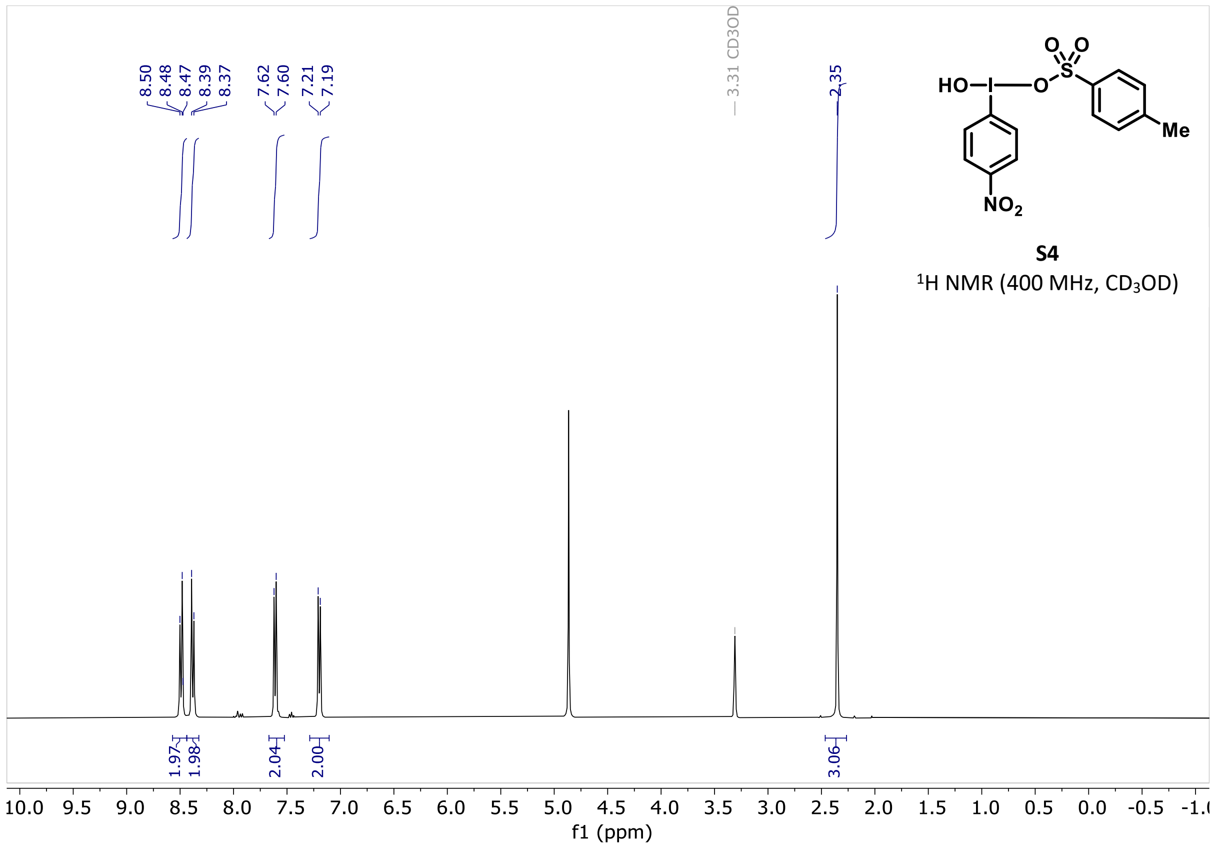

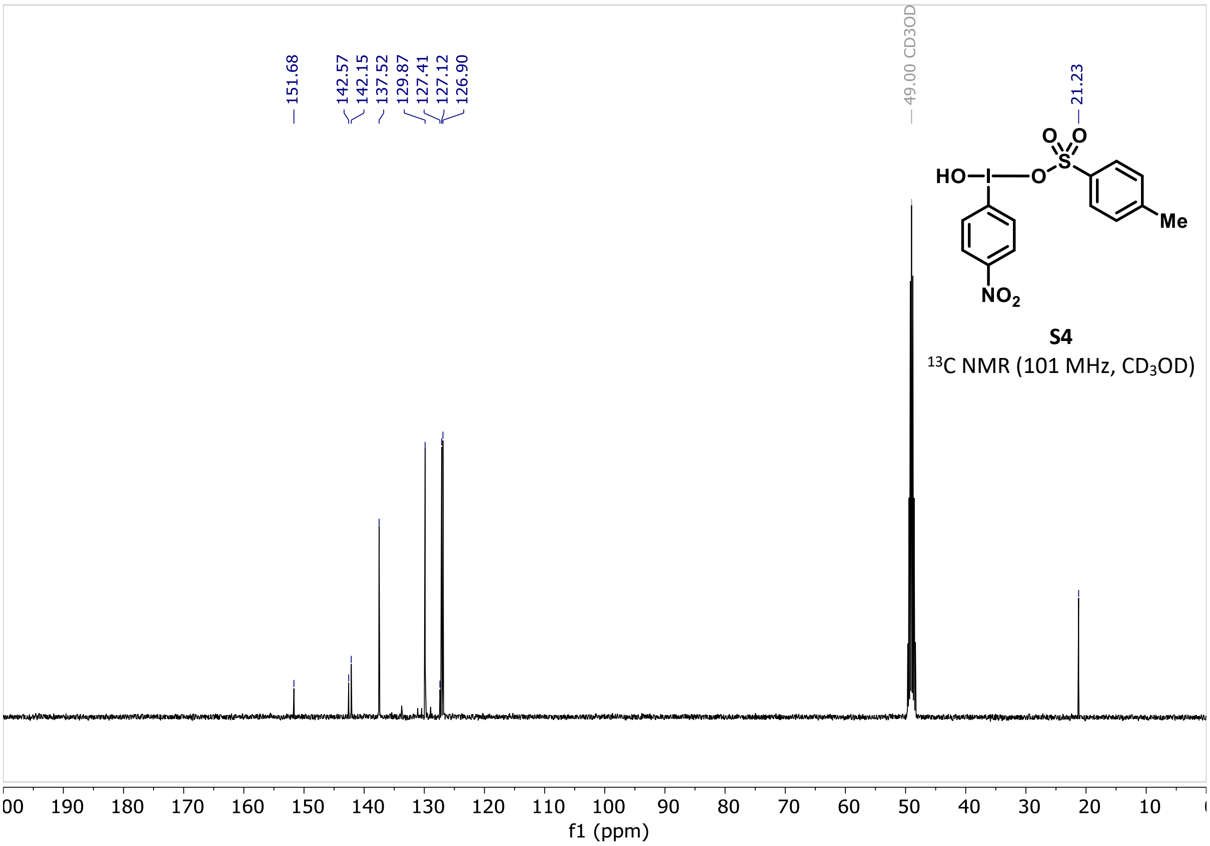

4-Nitrophenyliodonium(methyl (triphenylphosphoranylidene)acetate) tosylate (1i)

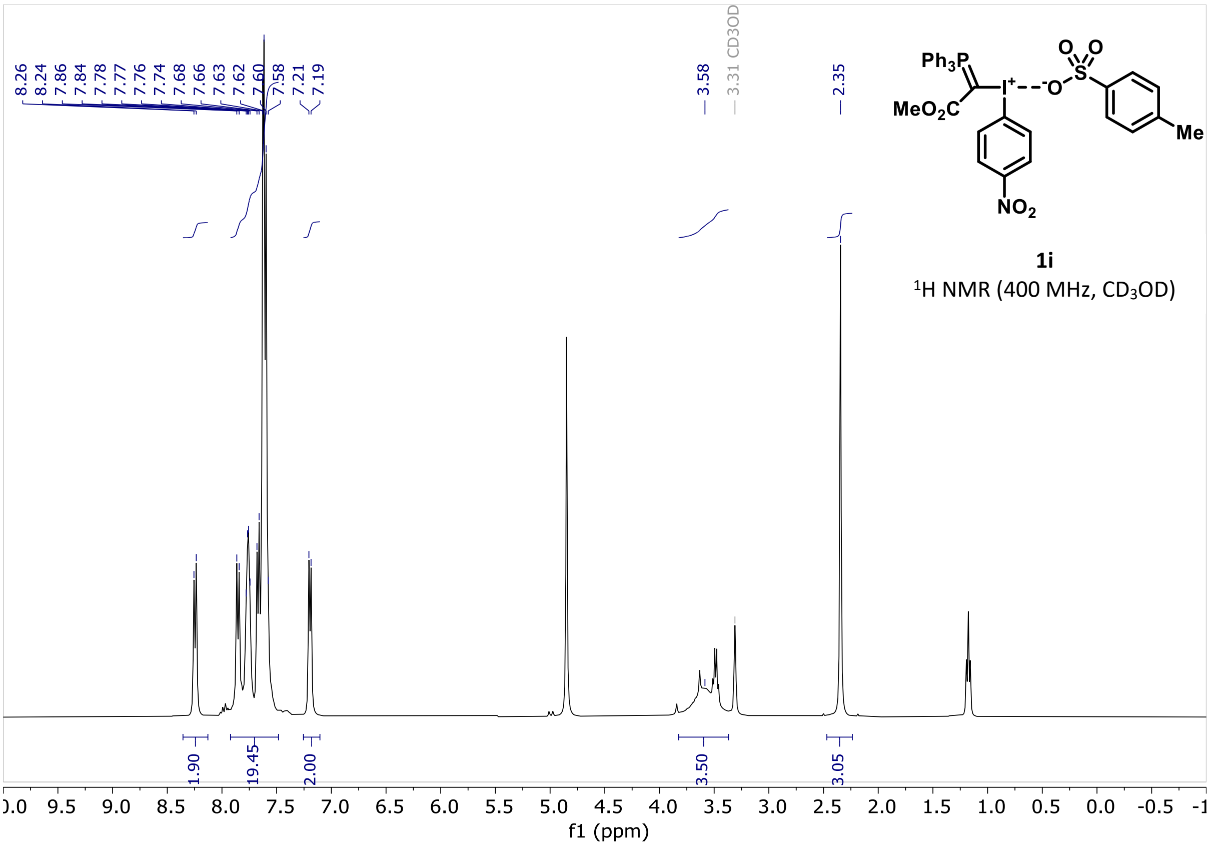

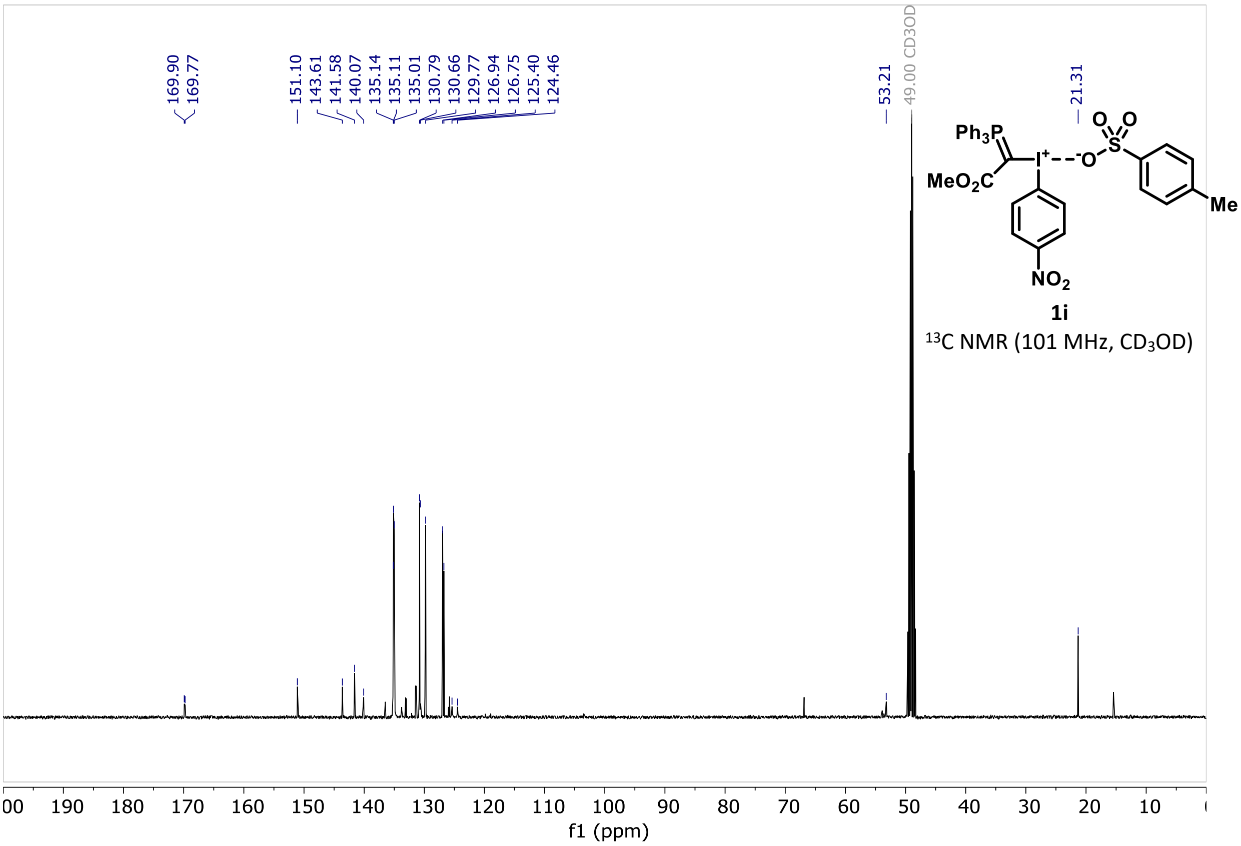

1i

^{13}C NMR (101 MHz, CD$_3$OD)

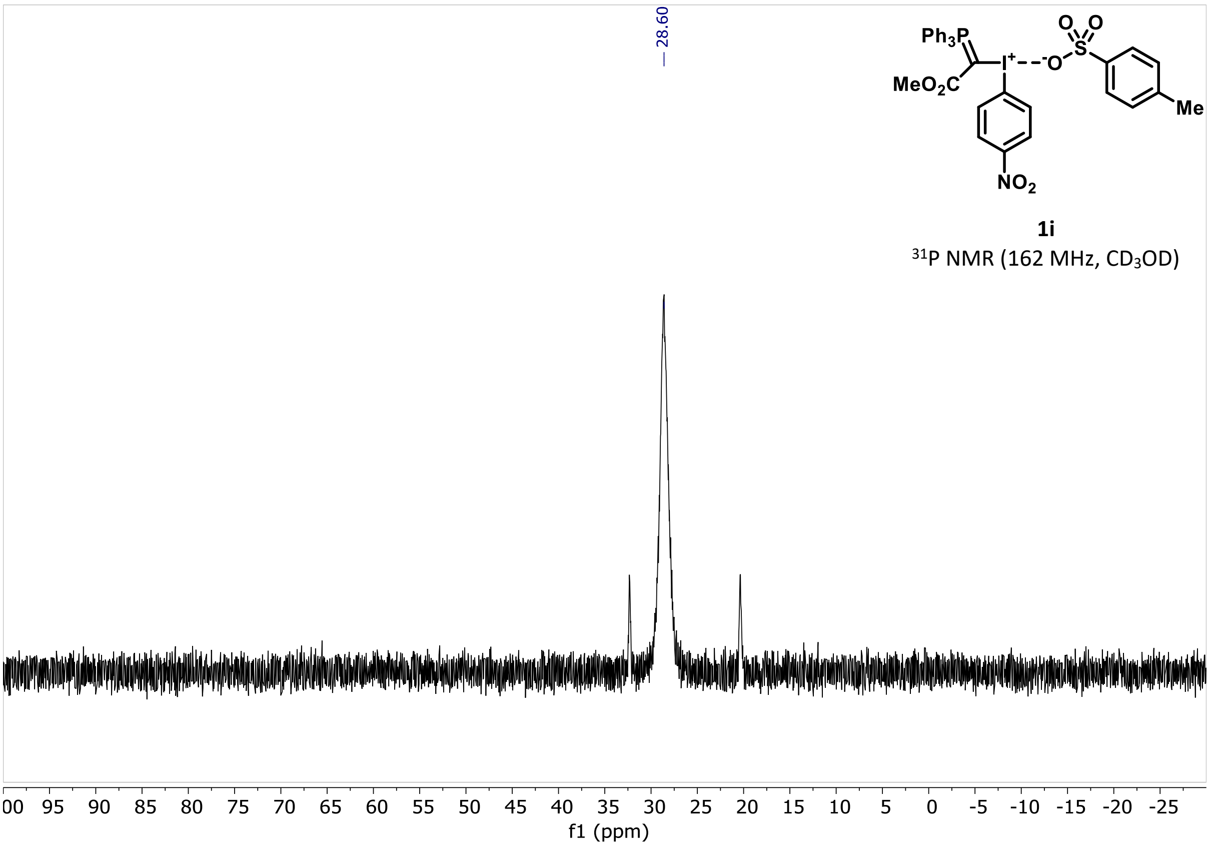

1i

^{31}P NMR (162 MHz, CD$_3$OD)

1-(Hydroxy(tosyloxy)iodo)-2-nitrobenzene (S5)

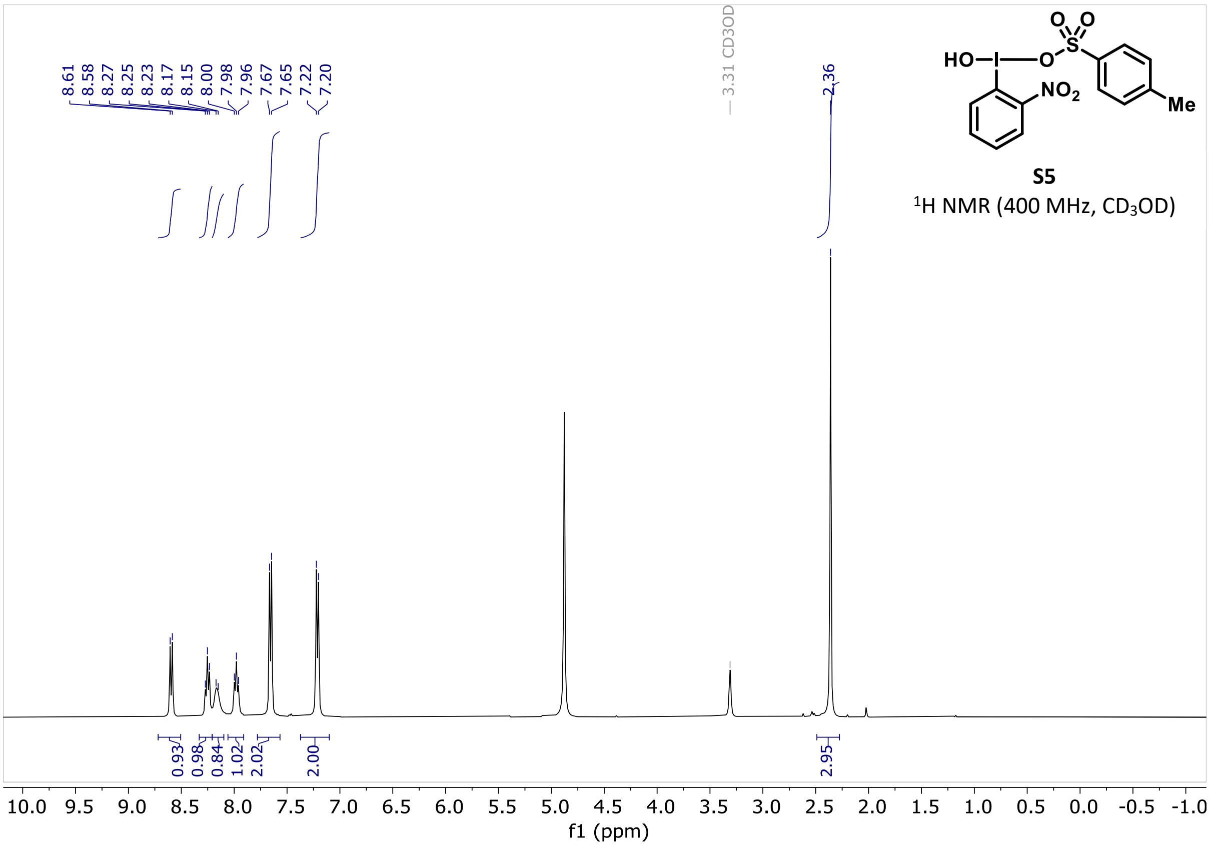

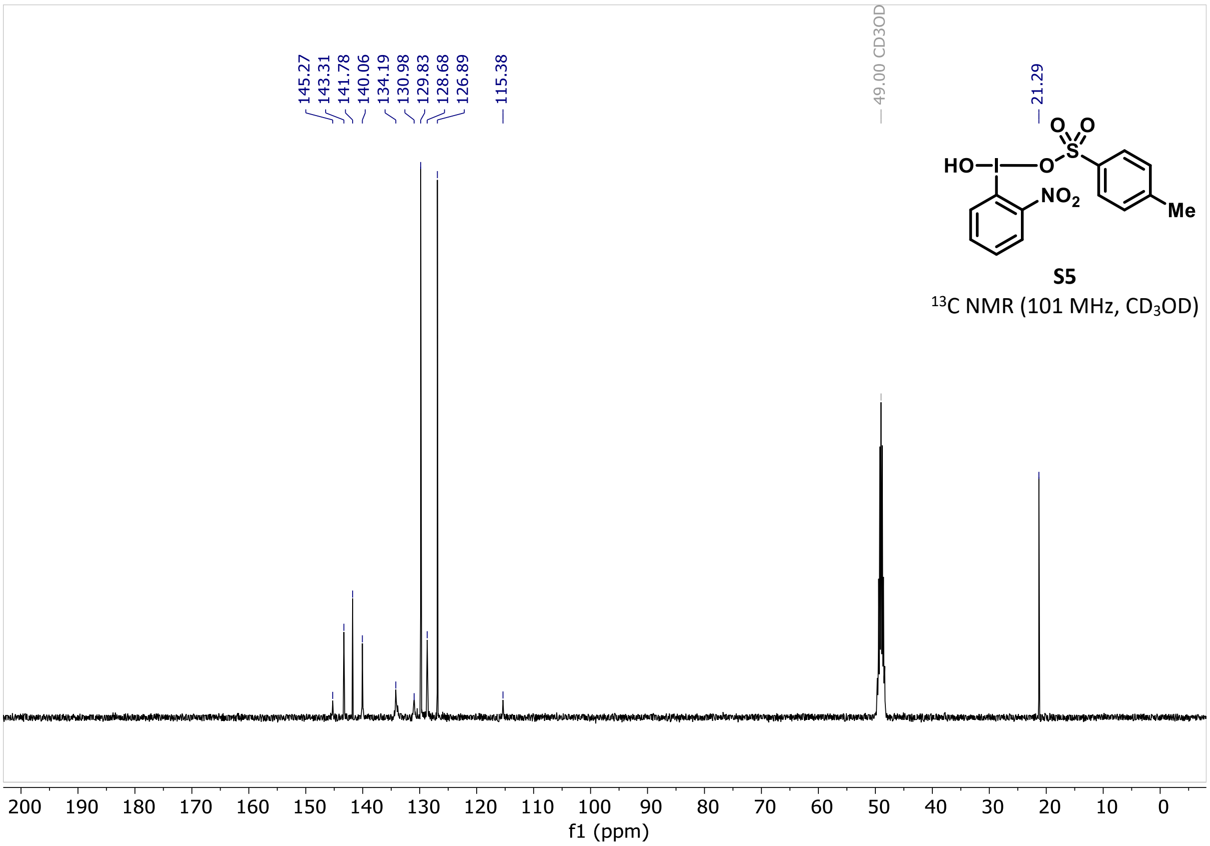

2-Nitrophenyliodonium(methyl (triphenylphosphoranylidene)acetate) tosylate (1j)

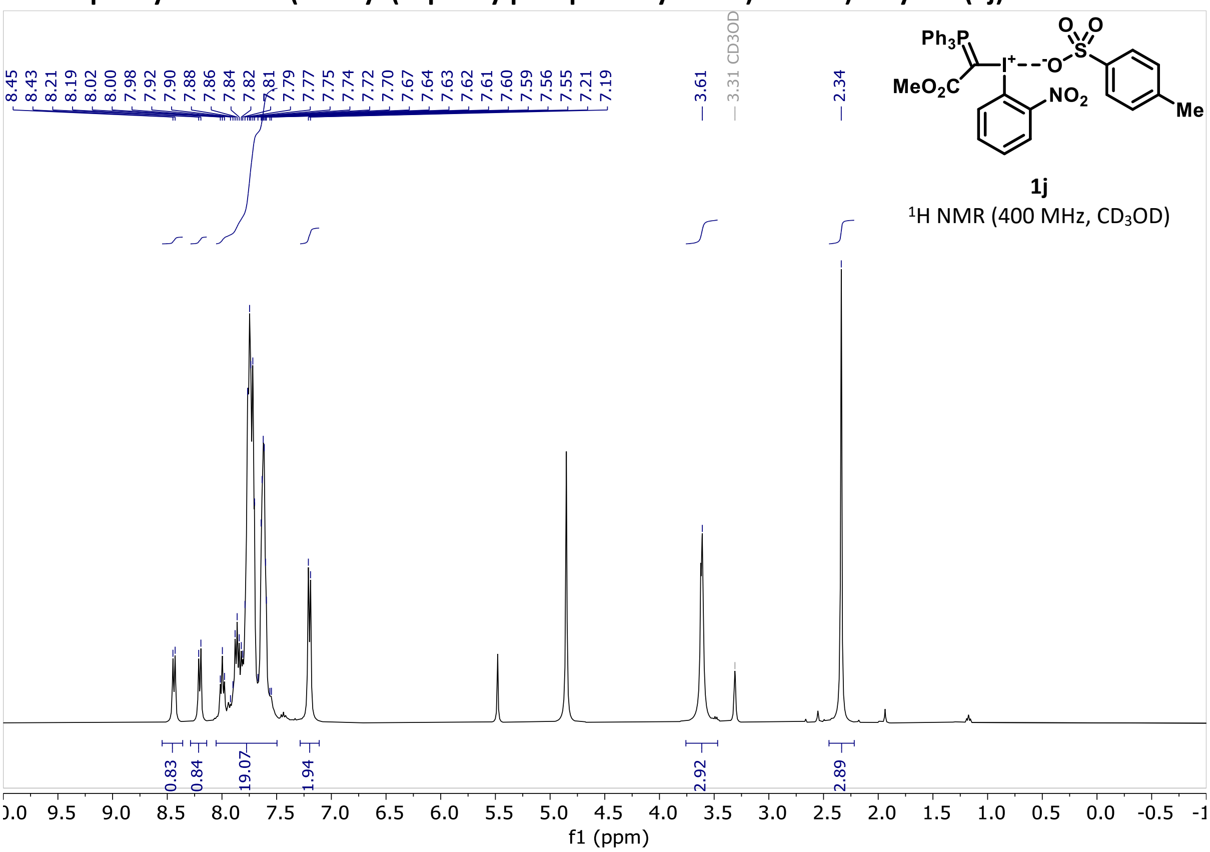

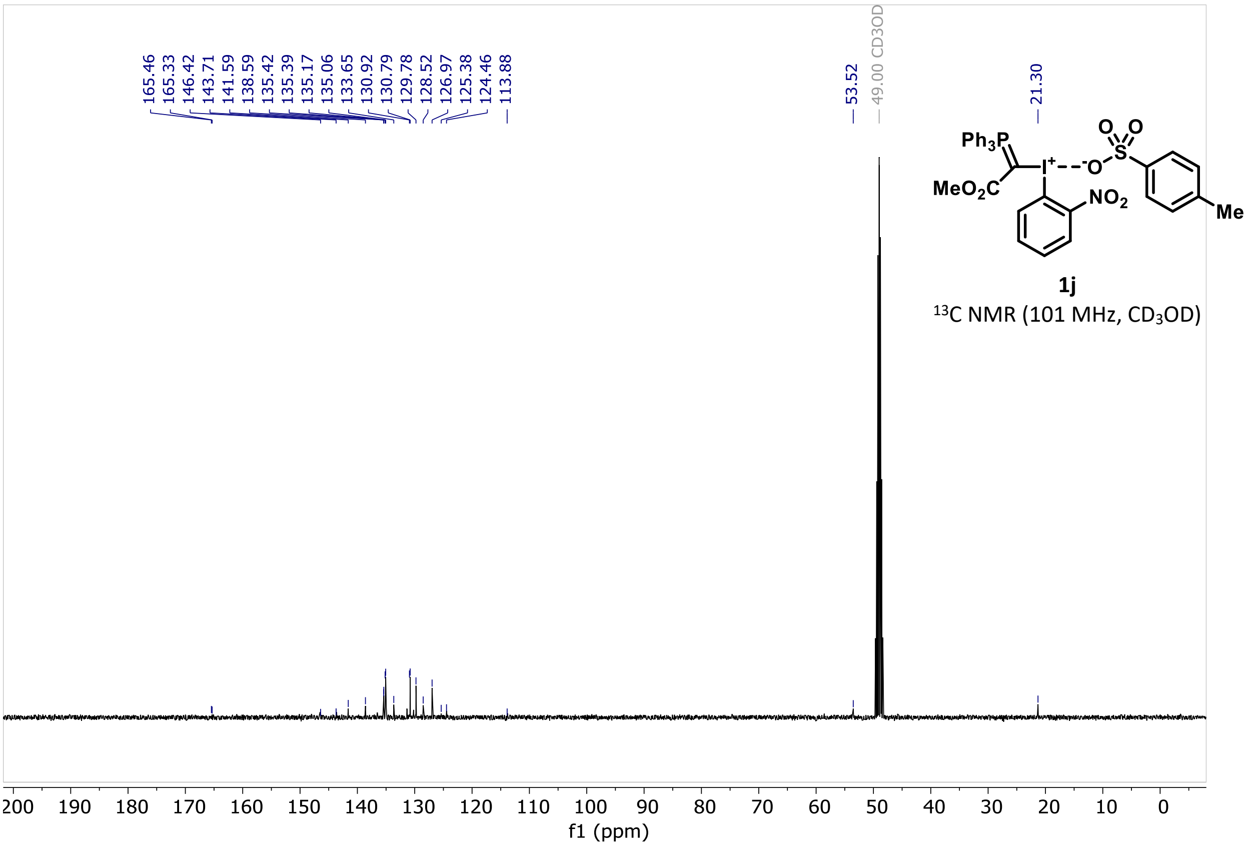

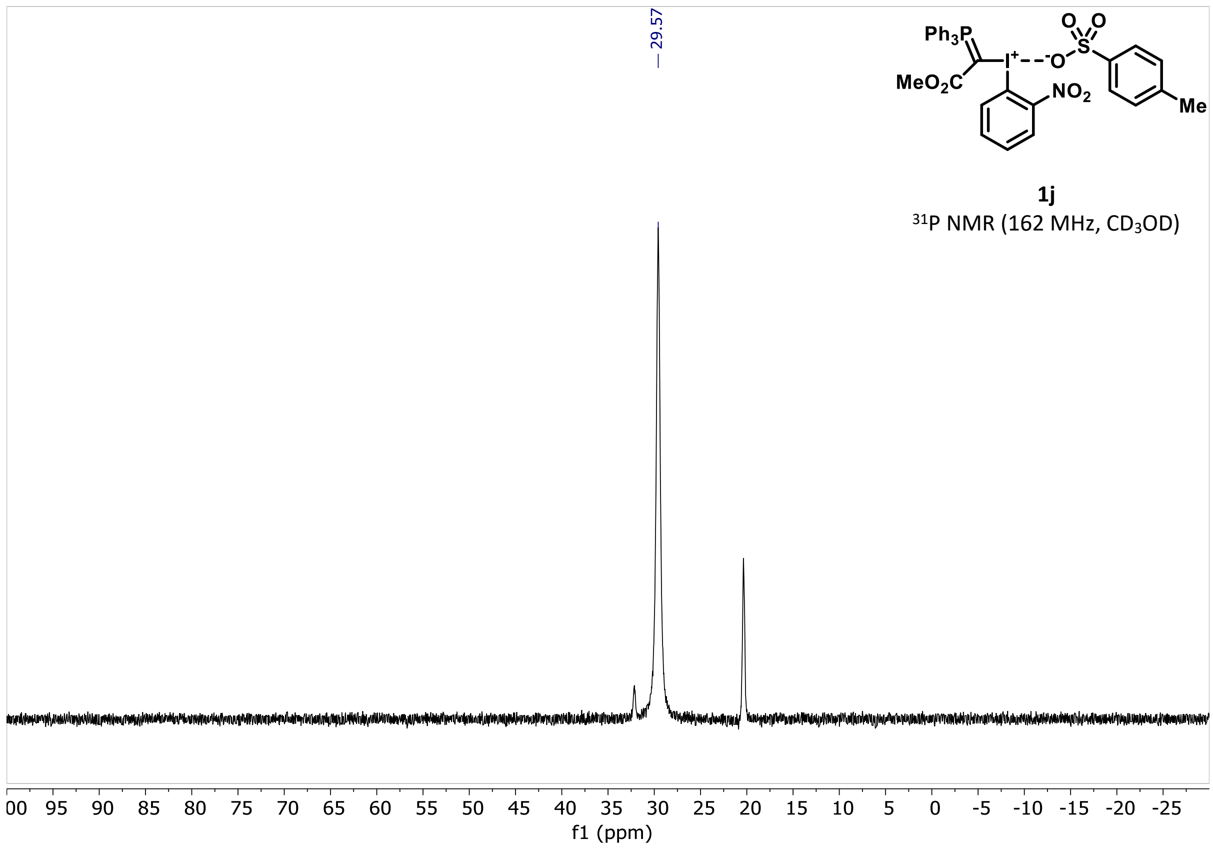

1-(Hydroxy(tosyloxy)iodo)-2,4,6-trimethylbenzene (S6)

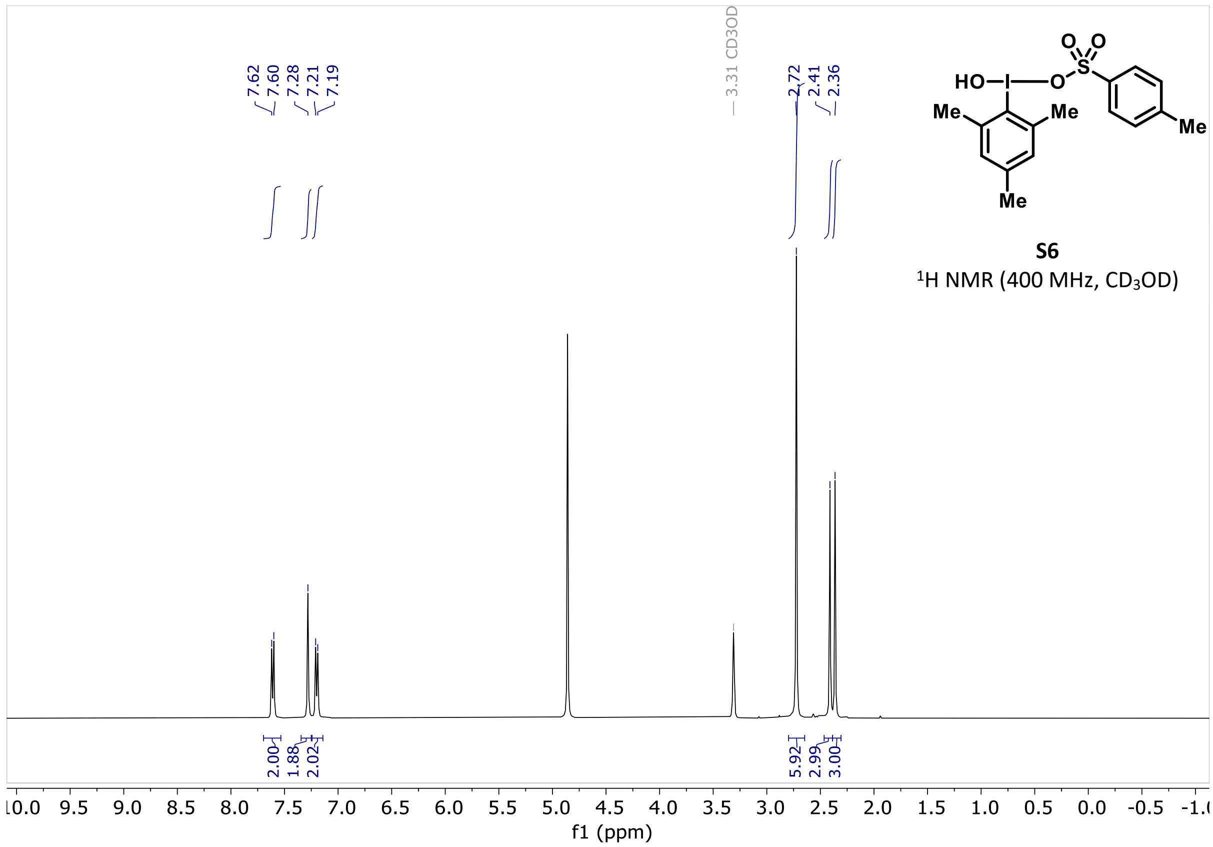

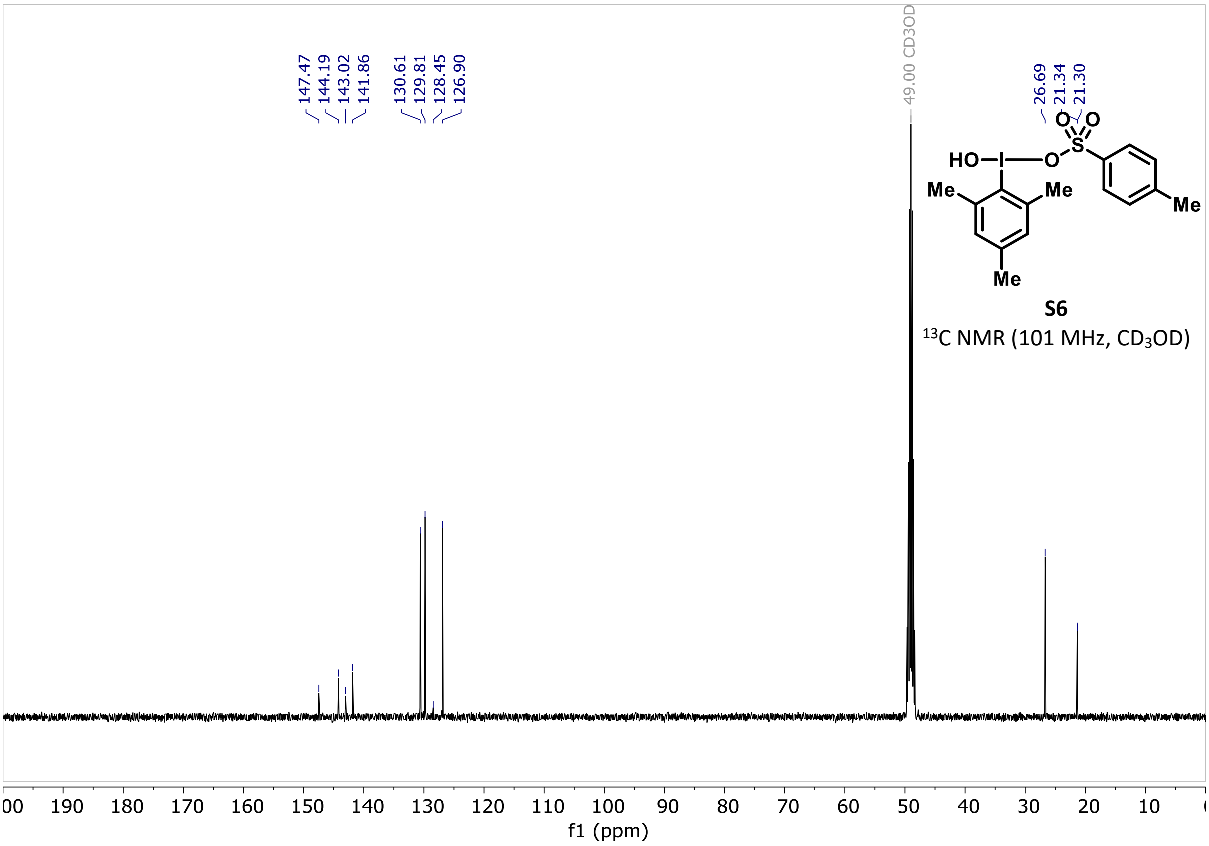

2,4,6-Trimethylphenyliodonium(methyl (triphenylphosphoranylidene)acetate) tosylate (1k)

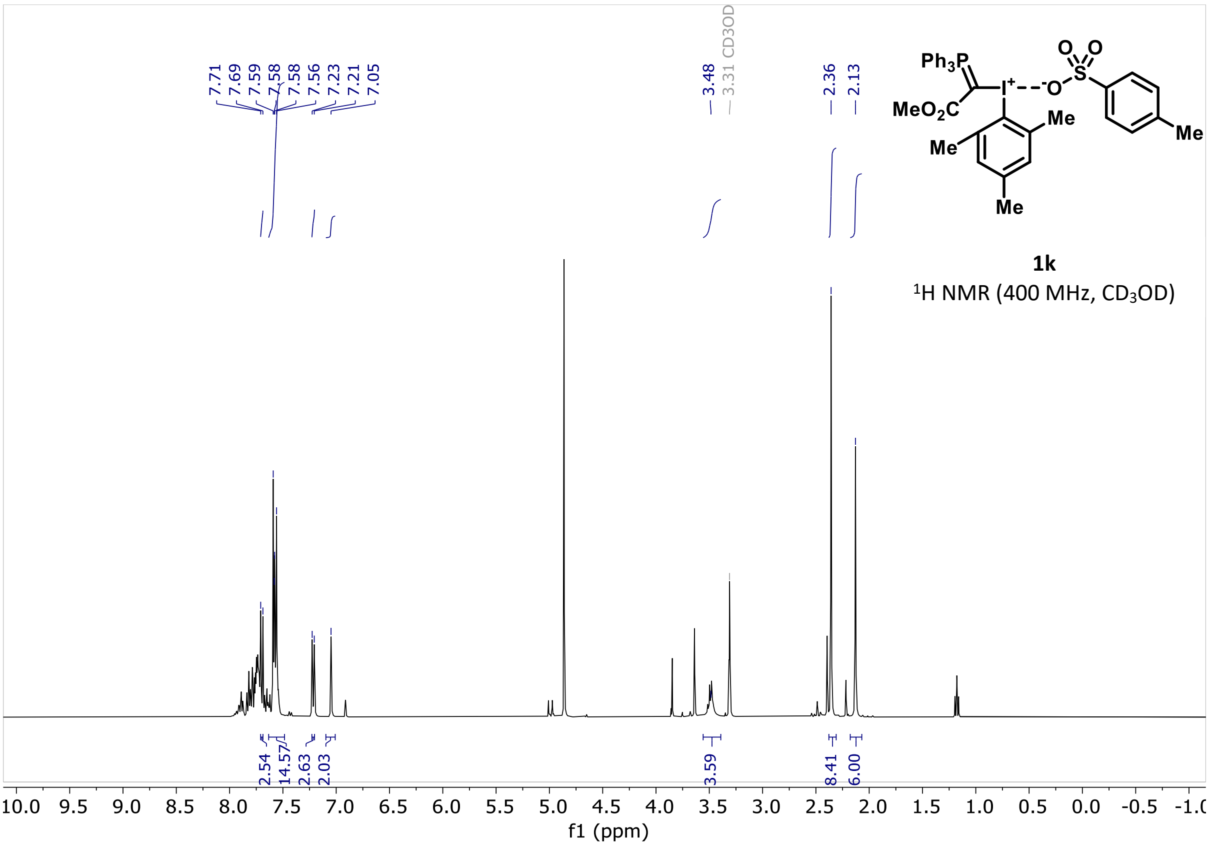

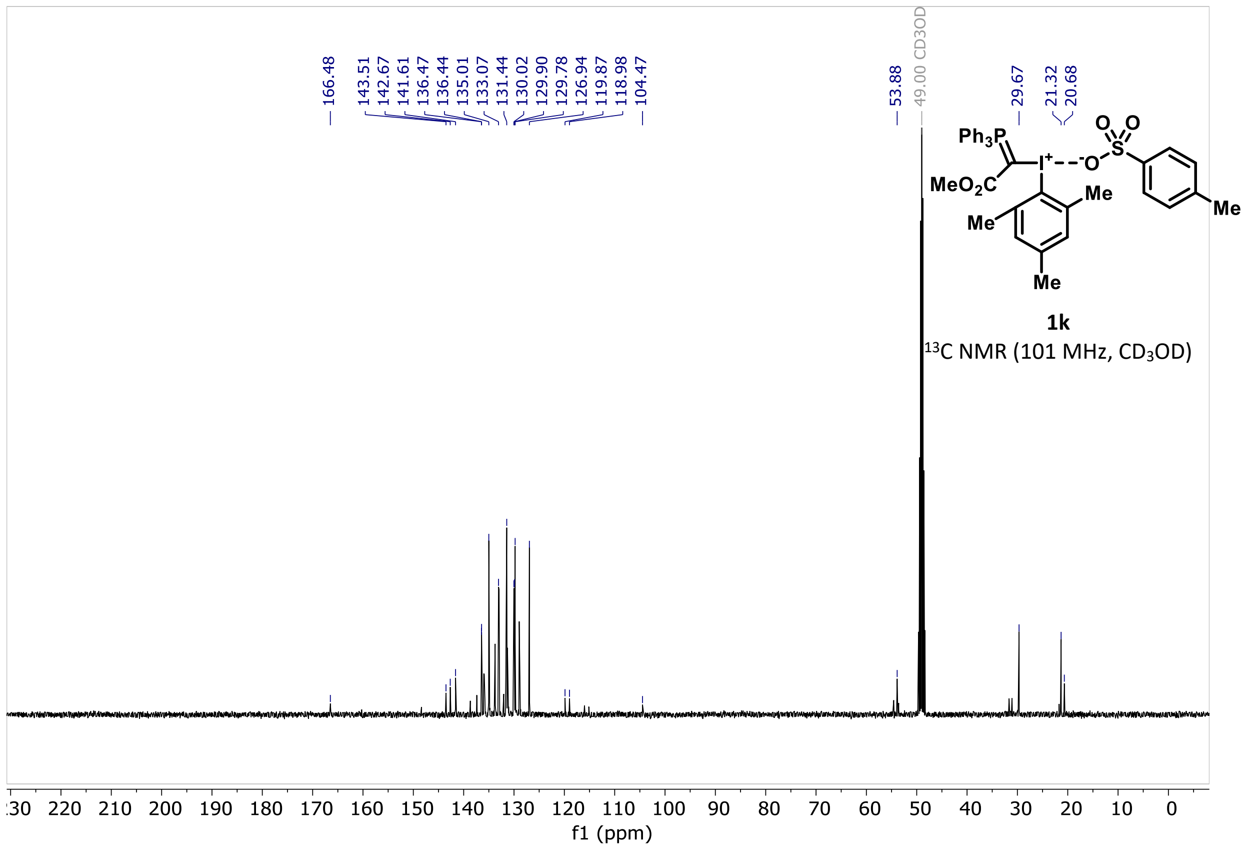
166.48
143.51
142.67
141.61
136.47
136.44
135.01
133.07
131.44
130.02
129.90
129.78
126.94
119.87
118.98
104.47
53.88
49.00 CD3OD
29.67
21.32
20.68
Ph3P
MeO2C
Me
Me
Me
Me
1k
13C NMR (101 MHz, CD3OD)
f1 (ppm)

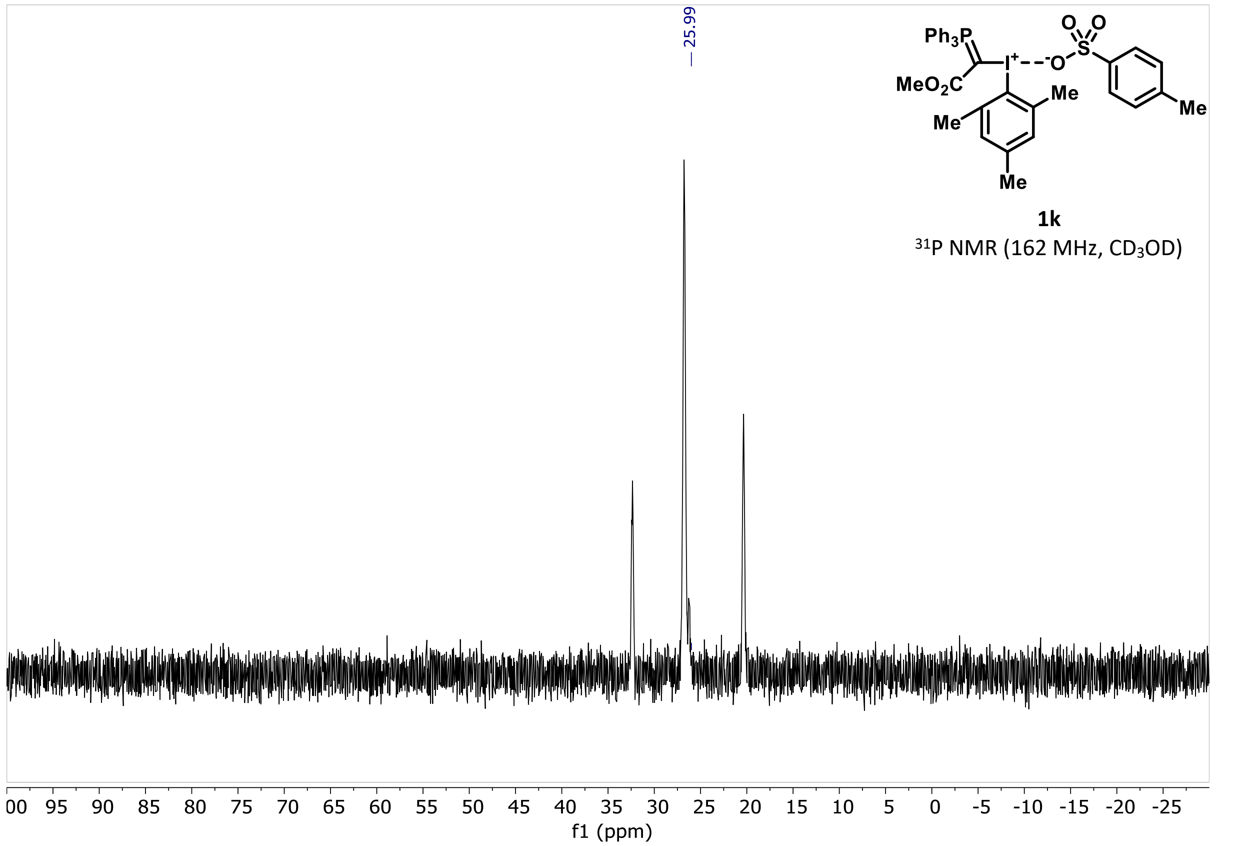
25.99
Ph3P
MeO2C
Me
Me
Me
Me
1k
31P NMR (162 MHz, CD3OD)
f1 (ppm)

Hydroxybenziodoxolone (S7)

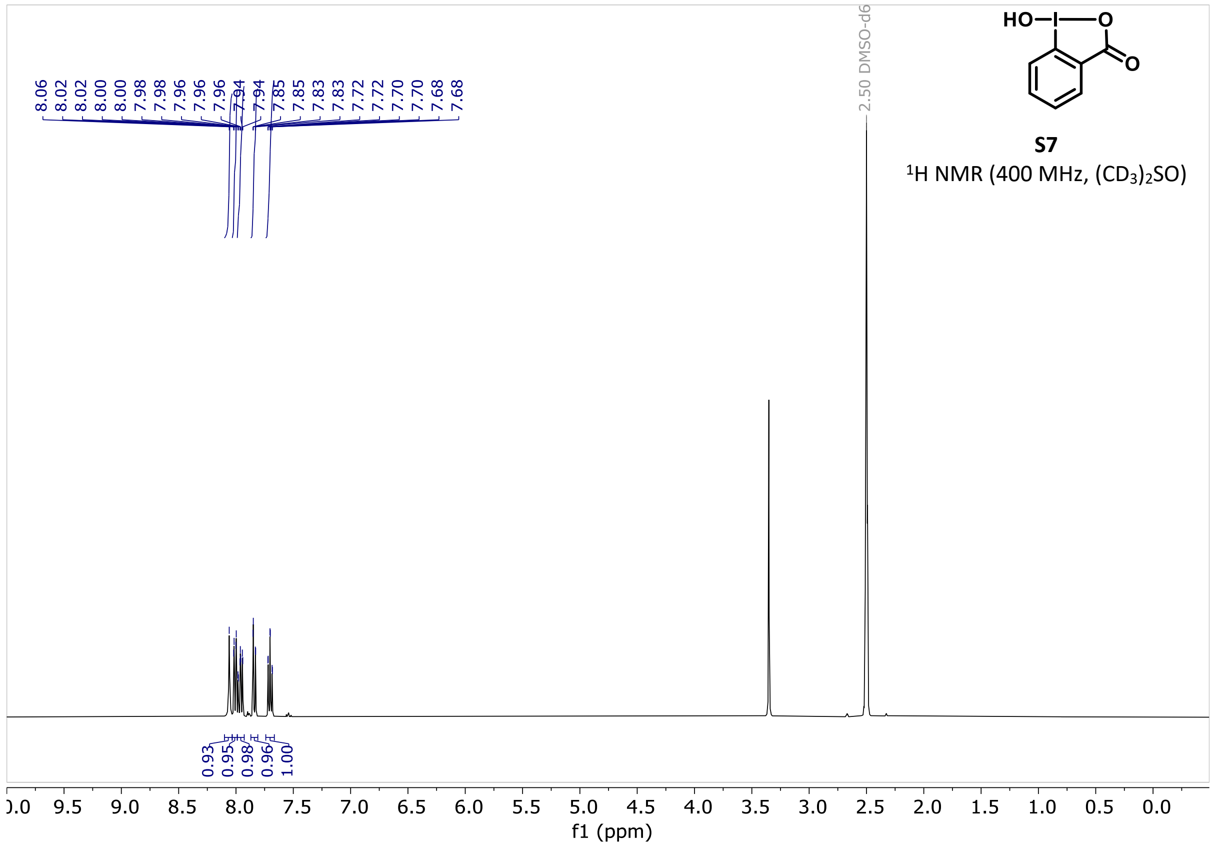

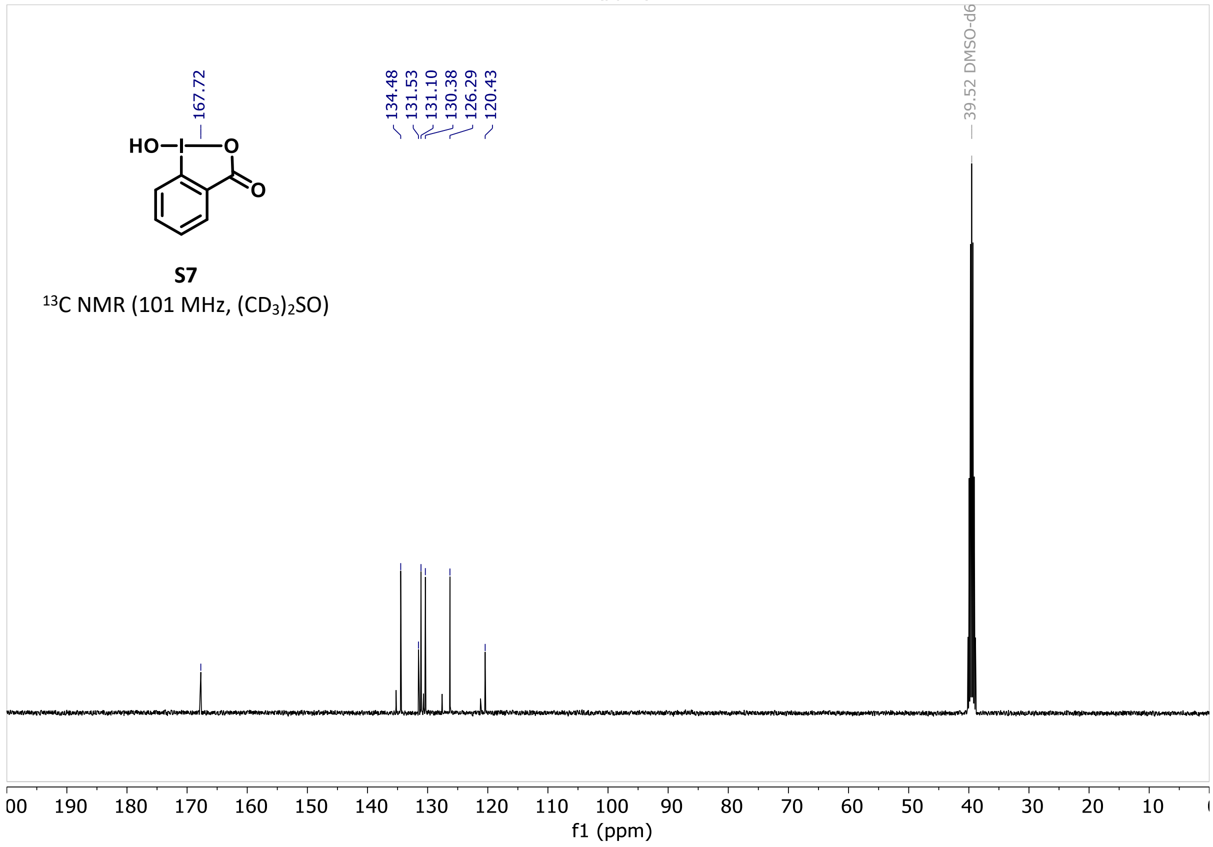

Acetoxybenziodoxolone (S8)

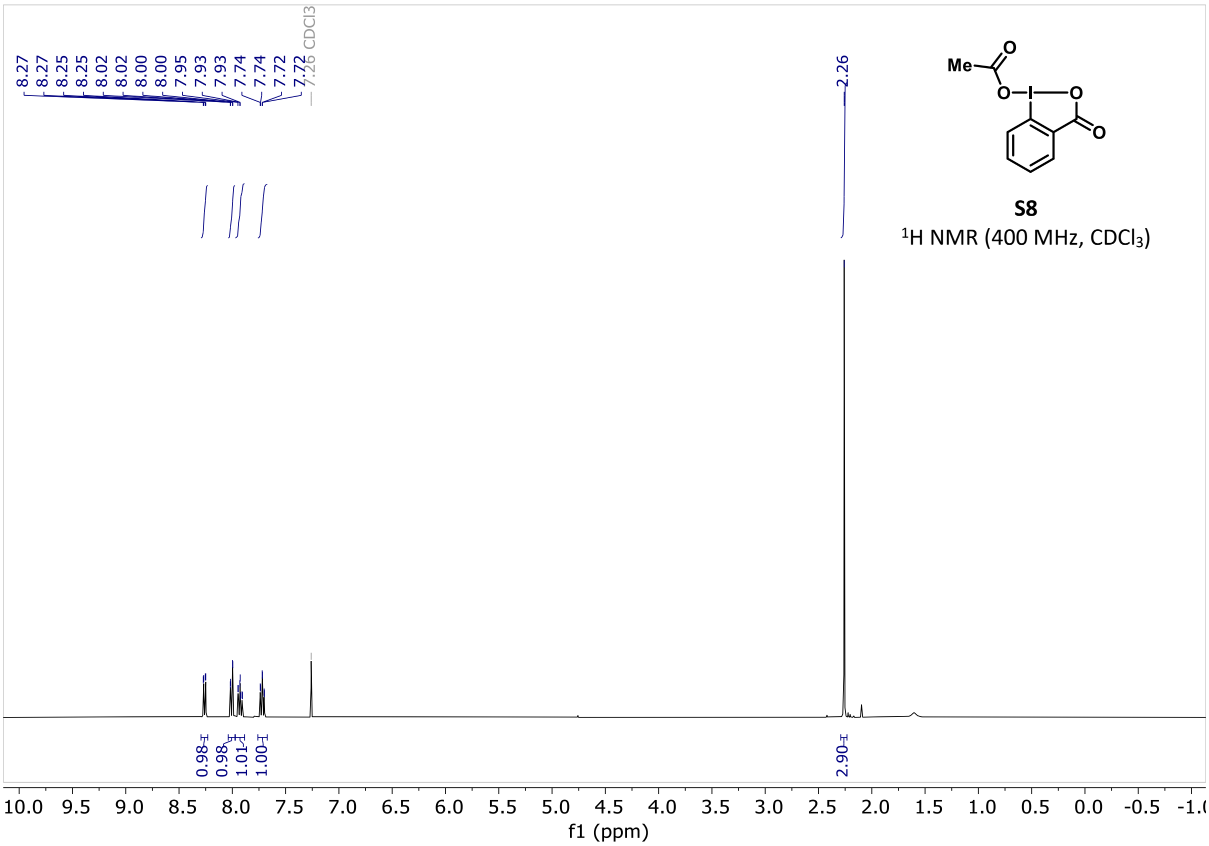

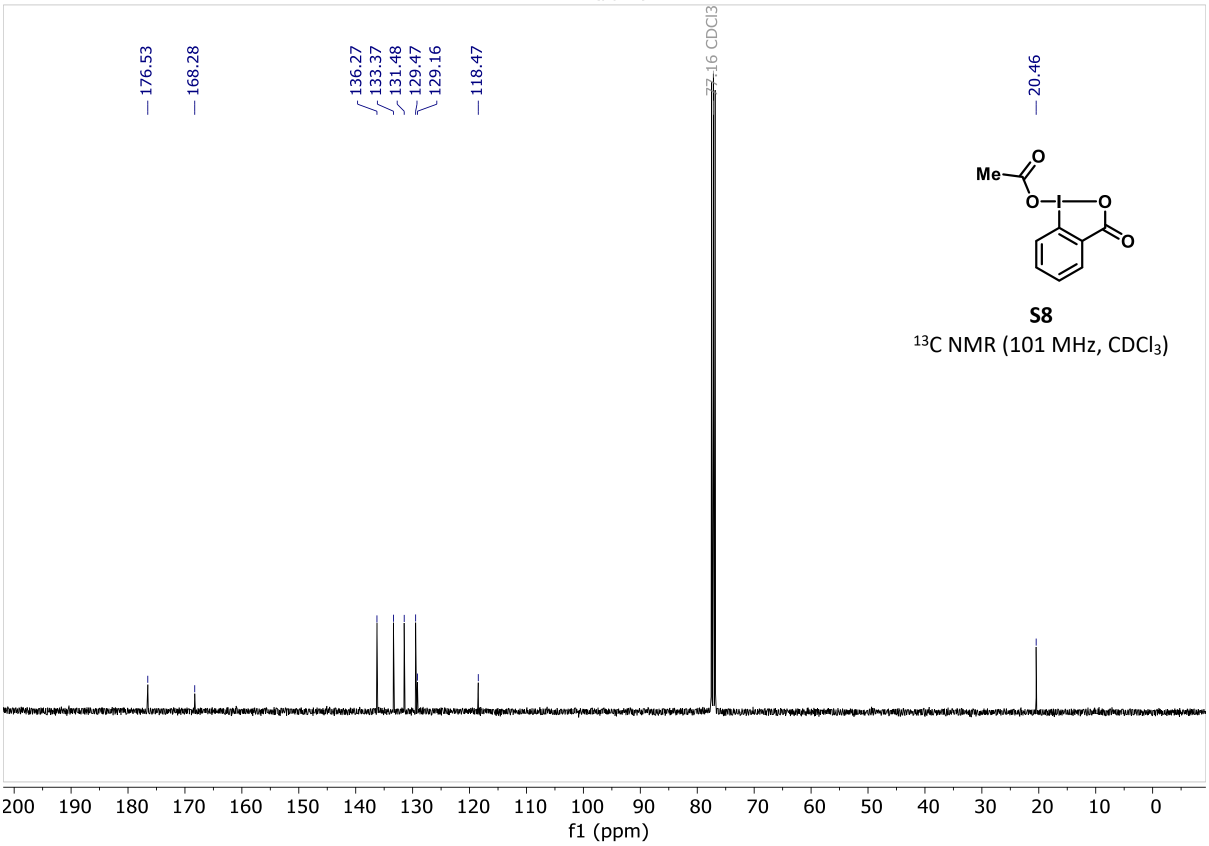

Methyl (triphenylphosphoranylidenenylbenziodoxolone)acetate (2)

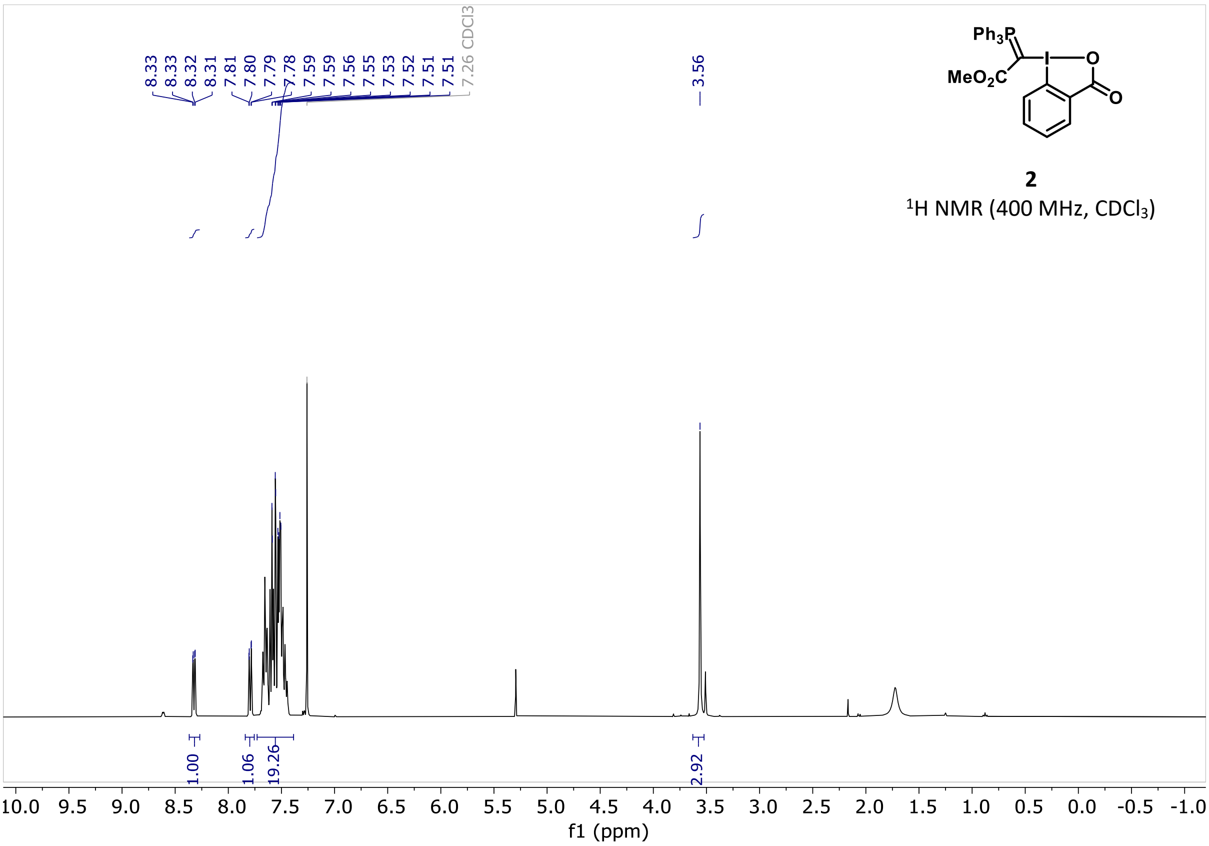

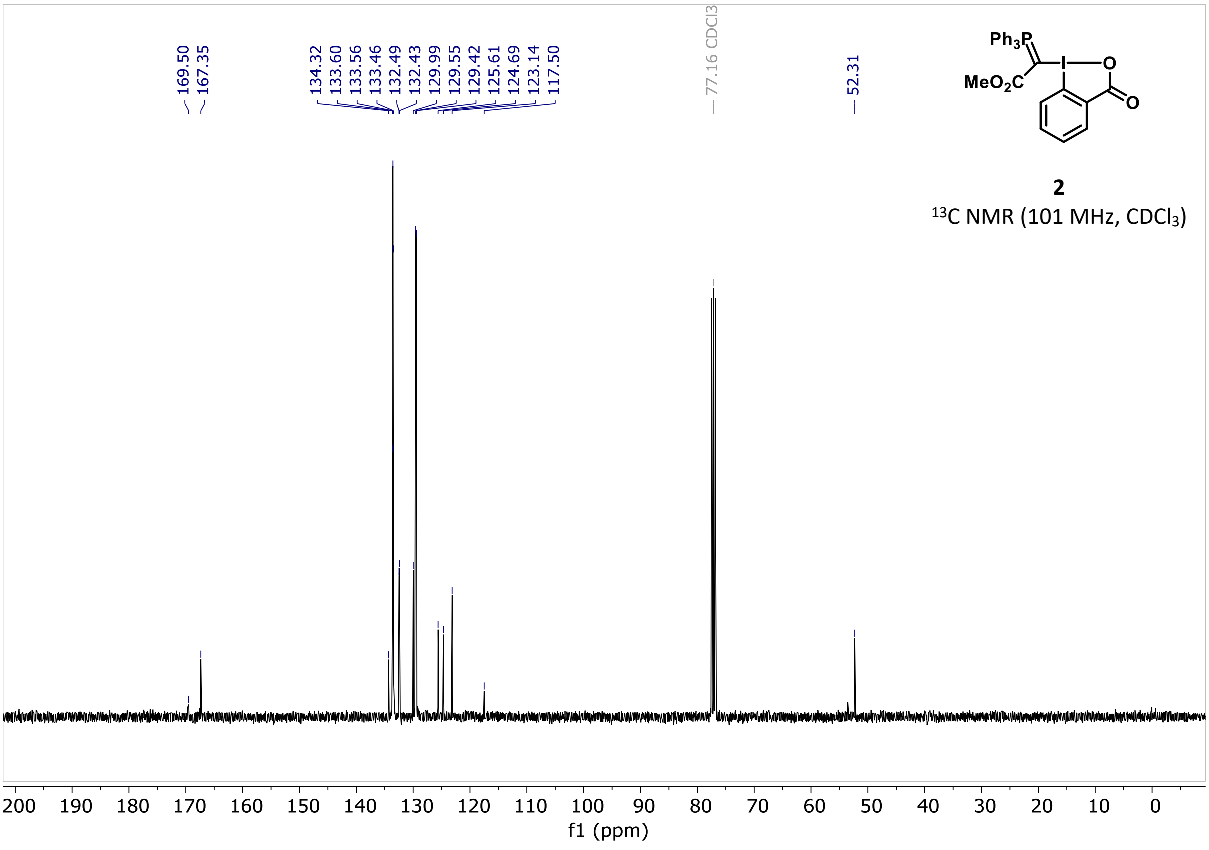

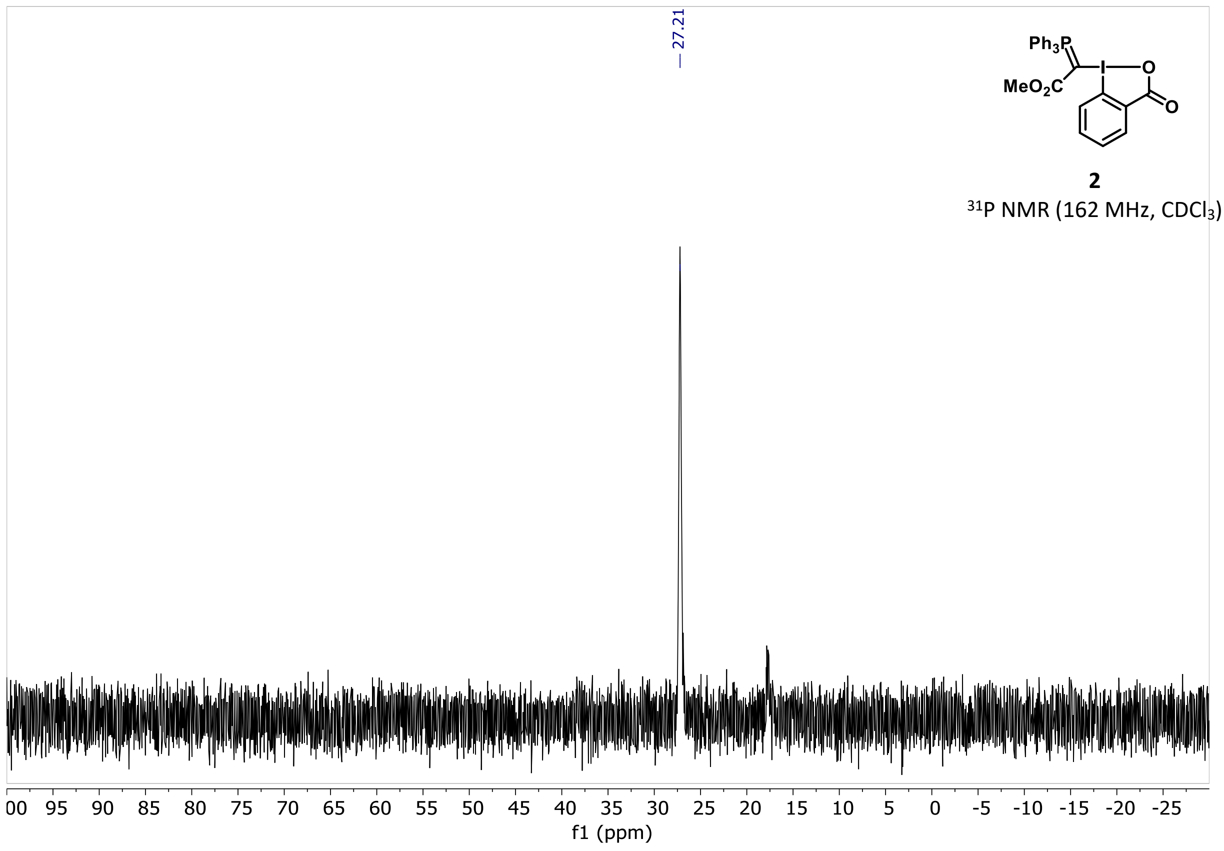

27.21
Ph3P
MeO2C
I
O
O
2
31P NMR (162 MHz, CDCl3)
00 95 90 85 80 75 70 65 60 55 50 45 40 35 30 25 20 15 10 5 0 -5 -10 -15 -20 -25
f1 (ppm)

6. References

[1] E. D. Matveeva, T. A. Podrugina, Y. K. Grishin, A. S. Pavlova, N. S. Zefirov, *Russ. J. Org. Chem.* **2007**, *43*, 201–206.

[2] E. D. Matveeva, T. A. Podrugina, M. A. Taranova, E. Y. Melikhova, R. Gleiter, N. S. Zefirov, *Tetrahedron* **2013**, *69*, 7395–7402.

[3] A. Nilova, P. A. Sibbald, E. J. Valente, G. A. González-Montiel, H. C. Richardson, K. S. Brown, P. H. Y. Cheong, D. R. Stuart, *Chem. – A Eur. J.* **2021**, *27*, 7168–7175.

[4] V. V. Zhdankin, O. Maydanovych, J. Herschbach, J. Bruno, E. D. Matveeva, N. S. Zefirov, *J. Org. Chem.* **2003**, *68*, 1018–1023.

[5] E. A. Merritt, V. M. T. Carneiro, L. F. Silva, B. Olofsson, *J. Org. Chem.* **2010**, *75*, 7416–7419.

[6] L. Kraszkiewicz, L. Skulski, *Arkivoc* **2003**, *6*, 120–125.

[7] T. Drennhaus, D. Leifert, J. Lammert, J. P. Drennhaus, K. Bergander, C. G. Daniliuc, A. Studer, *J. Am. Chem. Soc.* **2023**, *145*, 8665–8676.

[8] P. Eisenberger, S. Gischig, A. Togni, *Chem. - A Eur. Journal2* **2006**, *12*, 2579–2586.

[9] V. V. Zhdankin, O. Maydanovych, J. Herschbach, R. McDonald, R. R. Tykwinski, *J. Am. Chem. Soc.* **2002**, *124*, 11614–11615.

[10] C. Hansch, A. Leo, R. W. Taft, *Chem. Rev.* **1991**, *91*, 165–195.

[11] M. Ochiai, T. Sueda, K. Miyamoto, P. Kiprof, V. V. Zhdankin, *Angew. Chemie Int. Ed.* **2006**, *45*, 8203–8206.

[12] P. K. Sajith, C. H. Suresh, *Inorg. Chem.* **2012**, *51*, 967–977.

[13] S. Spange, R. Lungwitz, A. Schade, *J. Mol. Liq.* **2014**, *192*, 137–143.

[14] L. G. Bonnet, B. M. Kariuki, *Eur. J. Inorg. Chem.* **2006**, *2006*, 437–446.

[15] L. G. Sánchez, E. N. Castillo, H. Maldonado, D. Chávez, R. Somanathan, G. Aguirre, *Synth. Commun.* **2007**, *38*, 54–71.

[16] G. Reck, K. Lauritsen, L. Riesel, M. von Löwis, *Zeitschrift fur Naturforsch. - Sect. B J. Chem. Sci.* **1993**, *48*, 1760–1766.

[17] C. Xu, T. Li, P. Jiang, Y. J. Zhang, *Tetrahedron* **2020**, *76*, 131107.

[18] B. J. Cosier, A. M. Glazer, *J. Appl. Crystallogr.* **1986**, *19*, 105–107.

[19] U. Rigaku Oxford Diffraction (2018), CrysAlisPro Software system, version 1.171.40.45a, Rigaku Corporation, Oxford**.**

[20] O. V. Dolomanov, L. J. Bourhis, R. J. Gildea, J. A. K. Howard, H. Puschmann, *J. Appl. Cryst.* **2009**, *42*, 339–341.

[21] G. M. Sheldrick, *Acta Cryst. A* **2015**, *71*, 3–8.

[22] G. M. Sheldrick, *Acta Cryst. C* **2015**, *71*, 3–8.
